# Diretrizes Brasileiras de Medidas da Pressão Arterial Dentro e Fora do Consultório – 2023

**DOI:** 10.36660/abc.20240113

**Published:** 2024-02-23

**Authors:** Audes Diogenes de Magalhães Feitosa, Weimar Kunz Sebba Barroso, Decio Mion, Fernando Nobre, Marco Antonio Mota-Gomes, Paulo Cesar Brandão Veiga Jardim, Celso Amodeo, Adriana Camargo Oliveira, Alexandre Alessi, Ana Luiza Lima Sousa, Andréa Araujo Brandão, Andrea Pio-Abreu, Andrei C. Sposito, Angela Maria Geraldo Pierin, Annelise Machado Gomes de Paiva, Antonio Carlos de Souza Spinelli, Carlos Alberto Machado, Carlos Eduardo Poli-de-Figueiredo, Cibele Isaac Saad Rodrigues, Claudia Lucia de Moraes Forjaz, Diogo Pereira Santos Sampaio, Eduardo Costa Duarte Barbosa, Elizabete Viana de Freitas, Elizabeth do Espirito Santo Cestario, Elizabeth Silaid Muxfeldt, Emilton Lima, Erika Maria Gonçalves Campana, Fabiana Gomes Aragão Magalhães Feitosa, Fernanda Marciano Consolim-Colombo, Fernando Antônio de Almeida, Giovanio Vieira da Silva, Heitor Moreno, Helius Carlos Finimundi, Isabel Cristina Britto Guimarães, João Roberto Gemelli, José Augusto Soares Barreto-Filho, José Fernando Vilela-Martin, José Marcio Ribeiro, Juan Carlos Yugar-Toledo, Lucélia Batista Neves Cunha Magalhães, Luciano F. Drager, Luiz Aparecido Bortolotto, Marco Antonio de Melo Alves, Marcus Vinícius Bolívar Malachias, Mario Fritsch Toros Neves, Mayara Cedrim Santos, Nelson Dinamarco, Osni Moreira, Oswaldo Passarelli, Priscila Valverde de Oliveira Vitorino, Roberto Dischinger Miranda, Rodrigo Bezerra, Rodrigo Pinto Pedrosa, Rogerio Baumgratz de Paula, Rogério Toshiro Passos Okawa, Rui Manuel dos Santos Póvoa, Sandra C. Fuchs, Sandro Gonçalves de Lima, Sayuri Inuzuka, Sebastião Rodrigues Ferreira-Filho, Silvio Hock de Paffer Fillho, Thiago de Souza Veiga Jardim, Vanildo da Silva Guimarães, Vera Hermina Kalika Koch, Waléria Dantas Pereira Gusmão, Wille Oigman, Wilson Nadruz

**Affiliations:** 1 UFPE Recife PE Brasil Universidade Federal de Pernambuco (UFPE), Recife, PE – Brasil; 2 PROCAPE Recife PE Brasil Pronto Socorro Cardiológico de Pernambuco (PROCAPE), Recife, PE – Brasil; 3 IAPES Recife PE Brasil Instituto de Assistência, Pesquisa e Ensino em Saúde (IAPES), Recife, PE – Brasil; 4 Faculdade de Medicina UFG Goiânia GO Brasil Faculdade de Medicina da Universidade Federal de Goiás (UFG), Goiânia, GO – Brasil; 5 HCFMUSP São Paulo SP Brasil Hospital das Clínicas da Faculdade de Medicina da Universidade de São Paulo (HCFMUSP), São Paulo, SP – Brasil; 6 Faculdade de Medicina de Ribeirão Preto Universidade de São Paulo Ribeirão Preto SP Brasil Hospital das Clínicas da Faculdade de Medicina de Ribeirão Preto da Universidade de São Paulo, Ribeirão Preto, SP – Brasil; 7 Centro Universitário CESMAC Maceió AL Brasil Centro Universitário CESMAC, Maceió, AL – Brasil; 8 Hospital do Coração de Alagoas Maceió AL Brasil Hospital do Coração de Alagoas, Maceió, AL – Brasil; 9 Centro de Pesquisas Clínicas Dr. Marco Mota Maceió AL Brasil Centro de Pesquisas Clínicas Dr. Marco Mota, Maceió, AL – Brasil; 10 Hcor, Associação Beneficente Síria São Paulo SP Brasil Hcor, Associação Beneficente Síria, São Paulo, SP – Brasil; 11 Liga de Hipertensão Arterial Universidade Federal de Goiás Goiânia GO Brasil Liga de Hipertensão Arterial da Universidade Federal de Goiás (UFG), Goiânia, GO – Brasil; 12 Universidade Federal do Paraná Curitiba PR Brasil Universidade Federal do Paraná (UFPR), Curitiba, PR – Brasil; 13 Faculdade de Enfermagem Universidade Federal de Goiás GO Brasil Faculdade de Enfermagem da Universidade Federal de Goiás (UFG), Goiânia, GO – Brasil; 14 Universidade do Estado do Rio de Janeiro Rio de Janeiro RJ Brasil Universidade do Estado do Rio de Janeiro (UERJ), Rio de Janeiro, RJ – Brasil; 15 Universidade Estadual de Campinas Campinas São Paulo Brasil Universidade Estadual de Campinas (UNICAMP), Campinas, São Paulo – Brasil; 16 Universidade de São Paulo São Paulo SP Brasil Universidade de São Paulo (USP), São Paulo, SP – Brasil; 17 Cardiocentro Natal RN Brasil Cardiocentro, Natal, RN – Brasil; 18 Secretaria Municipal de Saúde de Campos do Jordão Campos do Jordão SP Brasil Secretaria Municipal de Saúde de Campos do Jordão, Campos do Jordão, SP – Brasil; 19 Pontifícia Universidade Católica do Rio Grande do Sul Porto Alegre RS Brasil Pontifícia Universidade Católica do Rio Grande do Sul, Porto Alegre, RS – Brasil; 20 Pontifícia Universidade Católica de São Paulo Faculdade de Ciências Médicas e da Saúde Sorocaba SP Brasil Pontifícia Universidade Católica de São Paulo, Faculdade de Ciências Médicas e da Saúde,Sorocaba, SP – Brasil; 21 Escola de Educação Física e Esporte Universidade de São Paulo São Paulo SP Brasil Escola de Educação Física e Esporte da Universidade de São Paulo (USP), São Paulo, SP – Brasil; 22 Hospital das Clínicas Universidade Federal de Goiás Goiânia GO Brasil Hospital das Clínicas da Universidade Federal de Goiás, Goiânia, GO – Brasil; 23 Universidade FEEVALE Porto Alegre RS Brasil Universidade FEEVALE, Porto Alegre, RS – Brasil; 24 Sociedade Brasileira de Geriatria e Gerontologia Rio de Janeiro RJ Brasil Sociedade Brasileira de Geriatria e Gerontologia, Rio de Janeiro, RJ – Brasil; 25 Centro Universitário de Votuporanga UNIFEV Votuporanga SP Brasil Centro Universitário de Votuporanga (UNIFEV), Votuporanga, SP – Brasil; 26 Universidade Federal do Rio de Janeiro Hospital Universitário Clementino Fraga Filho Programa de Hipertensão Arterial Resistente Rio de Janeiro RJ Brasil Universidade Federal do Rio de Janeiro (UFRJ), Hospital Universitário Clementino Fraga Filho – Programa de Hipertensão Arterial Resistente (ProHArt), Rio de Janeiro, RJ – Brasil; 27 IDOMED Universidade Estácio de Sá Rio de Janeiro RJ Brasil Instituto de Educação Médica (IDOMED) - Universidade Estácio de Sá, Rio de Janeiro, RJ – Brasil; 28 Pontifícia Universidade Católica do Paraná Curitiba PR Brasil Pontifícia Universidade Católica do Paraná, Curitiba, PR – Brasil; 29 Instituto de Medicina Integral Prof. Fernando Figueira Recife PE Brasil Instituto de Medicina Integral Prof. Fernando Figueira (IMIP), Recife, PE – Brasil; 30 Instituto do Coração Faculdade de Medicina Universidade de São Paulo São Paulo SP Brasil Instituto do Coração da Faculdade de Medicina da Universidade de São Paulo (Incor/FMUSP), São Paulo, SP – Brasil; 31 Universidade de Caxias do Sul Caxias do Sul RS Brasil Universidade de Caxias do Sul (UCS), Caxias do Sul, RS – Brasil; 32 Universidade Federal da Bahia Salvador BA Brasil Universidade Federal da Bahia (UFBA), Salvador, BA – Brasil; 33 Governo do Estado de Rondônia Porto Velho RO Brasil Governo do Estado de Rondônia, Porto Velho, RO – Brasil; 34 Universidade Federal de Sergipe São Cristóvão SE Brasil Universidade Federal de Sergipe, São Cristóvão, SE – Brasil; 35 Faculdade de Medicina de São José do Rio Preto São José do Rio Preto SP Brasil Faculdade de Medicina de São José do Rio Preto (FAMERP), São José do Rio Preto, SP – Brasil; 36 Faculdade Ciências Médicas de Minas Gerais Belo Horizonte MG Brasil Faculdade Ciências Médicas de Minas Gerais, Belo Horizonte, MG – Brasil; 37 Hospital Felício Rocho Belo Horizonte MG Brasil Hospital Felício Rocho, Belo Horizonte, MG – Brasil; 38 Faculdade Zarns Salvador BA Brasil Faculdade Zarns, Salvador, BA – Brasil; 39 Hospital Esperança Recife Recife PE Brasil Hospital Esperança Recife, Ilha do Leite, Recife, PE – Brasil; 40 Fundação Educacional Lucas Machado Belo Horizonte MG Brasil Fundação Educacional Lucas Machado (FELUMA), Belo Horizonte, MG – Brasil; 41 Colegiado de Medicina Universidade Estadual de Santa Cruz Ilhéus BA Brasil Colegiado de Medicina – Universidade Estadual de Santa Cruz (UESC), Ilhéus, BA – Brasil; 42 Clínica Gapski Moreira Curitiba PR Brasil Clínica Gapski Moreira, Curitiba, PR – Brasil; 43 Instituto Dante Pazzanese de Cardiologia São Paulo SP Brasil Instituto Dante Pazzanese de Cardiologia, São Paulo, SP – Brasil; 44 Pontifícia Universidade Católica de Goiás Goiânia GO Brasil Pontifícia Universidade Católica de Goiás, Goiânia, GO – Brasil; 45 Escola Paulista de Medicina Universidade Federal de São Paulo São Paulo SP Brasil Escola Paulista de Medicina, Universidade Federal de São Paulo (UNIFESP), São Paulo, SP – Brasil; 46 Laboratório de Imunopatologia Keizo Asami Universidade Federal de Pernambuco Recife PE Brasil Laboratório de Imunopatologia Keizo Asami da Universidade Federal de Pernambuco, Recife, PE – Brasil; 47 Universidade Federal de Juiz de Fora Juiz de Fora MG Brasil Universidade Federal de Juiz de Fora, Juiz de Fora, MG – Brasil; 48 Universidade Estadual de Maringá Maringá PR Brasil Universidade Estadual de Maringá, Maringá, PR – Brasil; 49 Universidade Federal do Rio Grande do Sul Porto Alegre RS Brasil Universidade Federal do Rio Grande do Sul (UFRGS), Porto Alegre, RS – Brasil; 50 Unidade de Hipertensão Arterial NIPEE UFG Goiânia GO Brasil Unidade de Hipertensão Arterial – NIPEE – LHA/UFG, Goiânia, GO – Brasil; 51 Nefroclinica de Uberlândia Uberlândia MG Brasil Nefroclinica de Uberlândia, Uberlândia, MG – Brasil; 52 Faculdade de Medicina de Olinda Olinda PE Brasil Faculdade de Medicina de Olinda, Olinda, PE – Brasil; 53 Hospital do Coração de Goiás Goiânia GO Brasil Hospital do Coração de Goiás, Goiânia, GO – Brasil; 54 Diagnóstico Cardíaco Recife PE Brasil Diagnóstico Cardíaco, Recife, PE – Brasil; 55 Hospital das Clínicas Faculdade de Medicina Universidade de São Paulo São Paulo SP Brasil Instituto da Criança e do adolescente do Hospital das Clínicas da Faculdade de Medicina da Universidade de São Paulo (HCFMUSP), São Paulo, SP – Brasil; 56 Universidade Estadual de Ciências da Saúde de Alagoas Maceió AL Brasil Universidade Estadual de Ciências da Saúde de Alagoas (UNCISAL), Maceió, AL – Brasil

**Table:** 

Diretrizes Brasileiras de Medidas da Pressão Arterial Dentro e Fora do Consultório – 2023	
O relatório abaixo lista as declarações de interesse conforme relatadas à SBC pelos especialistas durante o período de desenvolvimento deste posicionamento, 2021-2023.	
Especialista	Tipo de relacionamento com a indústria
Adriana Camargo Oliveira	Declaração financeira A - Pagamento de qualquer espécie e desde que economicamente apreciáveis, feitos a (i) você, (ii) ao seu cônjuge/ companheiro ou a qualquer outro membro que resida com você, (iii) a qualquer pessoa jurídica em que qualquer destes seja controlador, sócio, acionista ou participante, de forma direta ou indireta, recebimento por palestras, aulas, atuação como proctor de treinamentos, remunerações, honorários pagos por participações em conselhos consultivos, de investigadores, ou outros comitês, etc. Provenientes da indústria farmacêutica, de órteses, próteses, equipamentos e implantes, brasileiras ou estrangeiras: - Brace Pharma; EMS; Servier; Biolab. Outros relacionamentos Financiamento de atividades de educação médica continuada, incluindo viagens, hospedagens e inscrições para congressos e cursos, provenientes da indústria farmacêutica, de órteses, próteses, equipamentos e implantes, brasileiras ou estrangeiras: - Biolab: Bivolet; EMS: Xakilis.
Alexandre Alessi	Nada a ser declarado
Ana Luiza Lima Sousa	Nada a ser declarado
Andréa Araujo Brandão	Nada a ser declarado
Andrea Pio-Abreu	Nada a ser declarado
Andrei C. Sposito	Outros relacionamentos Financiamento de atividades de educação médica continuada, incluindo viagens, hospedagens e inscrições para congressos e cursos, provenientes da indústria farmacêutica, de órteses, próteses, equipamentos e implantes, brasileiras ou estrangeiras: - Novo Nordisk; Novartis.
Angela Maria Geraldo Pierin	Nada a ser declarado
Annelise Machado Gomes de Paiva	Nada a ser declarado
Antonio Carlos de Souza Spinelli	Declaração financeira A - Pagamento de qualquer espécie e desde que economicamente apreciáveis, feitos a (i) você, (ii) ao seu cônjuge/ companheiro ou a qualquer outro membro que resida com você, (iii) a qualquer pessoa jurídica em que qualquer destes seja controlador, sócio, acionista ou participante, de forma direta ou indireta, recebimento por palestras, aulas, atuação como proctor de treinamentos, remunerações, honorários pagos por participações em conselhos consultivos, de investigadores, ou outros comitês, etc. Provenientes da indústria farmacêutica, de órteses, próteses, equipamentos e implantes, brasileiras ou estrangeiras: - Novonordisk: Ozempic; Daiichi Sankyo: Benicar Triplo; Torrent: Rosucor; Boehringer: Jardiance. Outros relacionamentos Financiamento de atividades de educação médica continuada, incluindo viagens, hospedagens e inscrições para congressos e cursos, provenientes da indústria farmacêutica, de órteses, próteses, equipamentos e implantes, brasileiras ou estrangeiras: - Novonordisk: Ozempic; Daiichi Sankyo: Benicar Triplo; Torrent: Rosucor; Boehringer: Jardiance.
Audes Diogenes de Magalhães Feitosa	Declaração financeira A - Pagamento de qualquer espécie e desde que economicamente apreciáveis, feitos a (i) você, (ii) ao seu cônjuge/ companheiro ou a qualquer outro membro que resida com você, (iii) a qualquer pessoa jurídica em que qualquer destes seja controlador, sócio, acionista ou participante, de forma direta ou indireta, recebimento por palestras, aulas, atuação como proctor de treinamentos, remunerações, honorários pagos por participações em conselhos consultivos, de investigadores, ou outros comitês, etc. Provenientes da indústria farmacêutica, de órteses, próteses, equipamentos e implantes, brasileiras ou estrangeiras: - Consultor da Omron; aulas para Omron, Micromed e Cardios. Outros relacionamentos Financiamento de atividades de educação médica continuada, incluindo viagens, hospedagens e inscrições para congressos e cursos, provenientes da indústria farmacêutica, de órteses, próteses, equipamentos e implantes, brasileiras ou estrangeiras: - Omron.
Carlos Alberto Machado	Nada a ser declarado
Carlos Eduardo Poli-de-Figueiredo	Declaração financeira A - Pagamento de qualquer espécie e desde que economicamente apreciáveis, feitos a (i) você, (ii) ao seu cônjuge/ companheiro ou a qualquer outro membro que resida com você, (iii) a qualquer pessoa jurídica em que qualquer destes seja controlador, sócio, acionista ou participante, de forma direta ou indireta, recebimento por palestras, aulas, atuação como proctor de treinamentos, remunerações, honorários pagos por participações em conselhos consultivos, de investigadores, ou outros comitês, etc. Provenientes da indústria farmacêutica, de órteses, próteses, equipamentos e implantes, brasileiras ou estrangeiras: - AstraZeneca/Alexion: SHUA e Doença renal crônica; Bayer: Doença renal crônica; Fresenius, Life e Baxter: Diálise. Organização do evento MAYOPUCRS 2023 e de outros congressos de Sociedades Médicas com apoio de empresas. Sem conflitos na área de hipertensão arterial. B - Financiamento de pesquisas sob sua responsabilidade direta/pessoal (direcionado ao departamento ou instituição) provenientes da indústria farmacêutica, de órteses, próteses, equipamentos e implantes, brasileiras ou estrangeiras: - Pesquisador do Centro de Pesquisa Clínica da PUCRS. Sem estudos no momento. Outros relacionamentos Participação societária de qualquer natureza e qualquer valor economicamente apreciável de empresas na área de saúde, de ensino ou em empresas concorrentes ou fornecedoras da SBC: - Consultório médico (Nefromonitoriza). Participação em comitês de compras de materiais ou fármacos em instituições de saúde ou funções assemelhadas: - Chefe de Serviço de Nefrologia do HSL/PUCRS até 2023. Participação em órgãos governamentais de regulação, ou de defesa de direitos na área de cardiologia: - Participação em estudos financiados pelo Ministério da Saúde, CNPq e CAPES.
Celso Amodeo	Nada a ser declarado
Cibele Isaac Saad Rodrigues	Nada a ser declarado
Claudia Lucia de Moraes Forjaz	Nada a ser declarado
Décio Mion Junior	Nada a ser declarado
Diogo Pereira Santos Sampaio	Nada a ser declarado
Eduardo Costa Duarte Barbosa	Declaração financeira A - Pagamento de qualquer espécie e desde que economicamente apreciáveis, feitos a (i) você, (ii) ao seu cônjuge/ companheiro ou a qualquer outro membro que resida com você, (iii) a qualquer pessoa jurídica em que qualquer destes seja controlador, sócio, acionista ou participante, de forma direta ou indireta, recebimento por palestras, aulas, atuação como proctor de treinamentos, remunerações, honorários pagos por participações em conselhos consultivos, de investigadores, ou outros comitês, etc. Provenientes da indústria farmacêutica, de órteses, próteses, equipamentos e implantes, brasileiras ou estrangeiras: - Cardios: Arteris; Brace Pharma: Olmy Anlo; Servier: Triplixam; EMS: Bramicar. Outros relacionamentos Financiamento de atividades de educação médica continuada, incluindo viagens, hospedagens e inscrições para congressos e cursos, provenientes da indústria farmacêutica, de órteses, próteses, equipamentos e implantes, brasileiras ou estrangeiras: - Servier: Triplixam.
Elizabete Viana de Freitas	Nada a ser declarado
Elizabeth do Espírito Santo Cestario	Declaração financeira A - Pagamento de qualquer espécie e desde que economicamente apreciáveis, feitos a (i) você, (ii) ao seu cônjuge/ companheiro ou a qualquer outro membro que resida com você, (iii) a qualquer pessoa jurídica em que qualquer destes seja controlador, sócio, acionista ou participante, de forma direta ou indireta, recebimento por palestras, aulas, atuação como proctor de treinamentos, remunerações, honorários pagos por participações em conselhos consultivos, de investigadores, ou outros comitês, etc. Provenientes da indústria farmacêutica, de órteses, próteses, equipamentos e implantes, brasileiras ou estrangeiras: - Servier: Triplixam; Torrent: Rosucor. B - Financiamento de pesquisas sob sua responsabilidade direta/pessoal (direcionado ao departamento ou instituição) provenientes da indústria farmacêutica, de órteses, próteses, equipamentos e implantes, brasileiras ou estrangeiras: - Servier: Vastarel; Libbs: Hipertensão. Outros relacionamentos Financiamento de atividades de educação médica continuada, incluindo viagens, hospedagens e inscrições para congressos e cursos, provenientes da indústria farmacêutica, de órteses, próteses, equipamentos e implantes, brasileiras ou estrangeiras: - Servier; Torrent.
Elizabeth Silaid Muxfeldt	Nada a ser declarado
Emilton Lima Júnior	Declaração financeira A - Pagamento de qualquer espécie e desde que economicamente apreciáveis, feitos a (i) você, (ii) ao seu cônjuge/ companheiro ou a qualquer outro membro que resida com você, (iii) a qualquer pessoa jurídica em que qualquer destes seja controlador, sócio, acionista ou participante, de forma direta ou indireta, recebimento por palestras, aulas, atuação como proctor de treinamentos, remunerações, honorários pagos por participações em conselhos consultivos, de investigadores, ou outros comitês, etc. Provenientes da indústria farmacêutica, de órteses, próteses, equipamentos e implantes, brasileiras ou estrangeiras: - Servier: Hipertensão; Novo Nordisk: Diabetes/Obesidade; Daiichi Sankyo: Liliana; Chiesi: DPOC; Novartis: Dislipidemia. Outros relacionamentos Financiamento de atividades de educação médica continuada, incluindo viagens, hospedagens e inscrições para congressos e cursos, provenientes da indústria farmacêutica, de órteses, próteses, equipamentos e implantes, brasileiras ou estrangeiras: - Servier: Hipertensão; Novo Nordisk: Diabetes/Obesidade; Daiichi Sankyo: Liliana; Chiesi: DPOC; Novartis: Dislipidemia.
Erika Maria Gonçalves Campana	Declaração financeira A - Pagamento de qualquer espécie e desde que economicamente apreciáveis, feitos a (i) você, (ii) ao seu cônjuge/ companheiro ou a qualquer outro membro que resida com você, (iii) a qualquer pessoa jurídica em que qualquer destes seja controlador, sócio, acionista ou participante, de forma direta ou indireta, recebimento por palestras, aulas, atuação como proctor de treinamentos, remunerações, honorários pagos por participações em conselhos consultivos, de investigadores, ou outros comitês, etc. Provenientes da indústria farmacêutica, de órteses, próteses, equipamentos e implantes, brasileiras ou estrangeiras: - Servier: Perindopril/Hipertensão; Biolab: Pressplus/Hipertensão. Outros relacionamentos Financiamento de atividades de educação médica continuada, incluindo viagens, hospedagens e inscrições para congressos e cursos, provenientes da indústria farmacêutica, de órteses, próteses, equipamentos e implantes, brasileiras ou estrangeiras: - Servier: Perindopril/Hipertensão.
Fabiana Gomes Aragão Magalhães Feitosa	Nada a ser declarado
Fernanda M. Consolim-Colombo	Declaração financeira A - Pagamento de qualquer espécie e desde que economicamente apreciáveis, feitos a (i) você, (ii) ao seu cônjuge/ companheiro ou a qualquer outro membro que resida com você, (iii) a qualquer pessoa jurídica em que qualquer destes seja controlador, sócio, acionista ou participante, de forma direta ou indireta, recebimento por palestras, aulas, atuação como proctor de treinamentos, remunerações, honorários pagos por participações em conselhos consultivos, de investigadores, ou outros comitês, etc. Provenientes da indústria farmacêutica, de órteses, próteses, equipamentos e implantes, brasileiras ou estrangeiras: - Daiichi Sankyo; Merck; Servier; AstraZeneca. Outros relacionamentos Financiamento de atividades de educação médica continuada, incluindo viagens, hospedagens e inscrições para congressos e cursos, provenientes da indústria farmacêutica, de órteses, próteses, equipamentos e implantes, brasileiras ou estrangeiras: - Daiichi Sankyo; Servier.
Fernando Antônio de Almeida	Nada a ser declarado
Fernando Nobre	Nada a ser declarado
Giovanio Vieira da Silva	Nada a ser declarado
Heitor Moreno Júnior	Nada a ser declarado
Helius Carlos Finimundi	Nada a ser declarado
Isabel Cristina Britto Guimarães	Nada a ser declarado
João Roberto Gemelli	Declaração financeira A - Pagamento de qualquer espécie e desde que economicamente apreciáveis, feitos a (i) você, (ii) ao seu cônjuge/ companheiro ou a qualquer outro membro que resida com você, (iii) a qualquer pessoa jurídica em que qualquer destes seja controlador, sócio, acionista ou participante, de forma direta ou indireta, recebimento por palestras, aulas, atuação como proctor de treinamentos, remunerações, honorários pagos por participações em conselhos consultivos, de investigadores, ou outros comitês, etc. Provenientes da indústria farmacêutica, de órteses, próteses, equipamentos e implantes, brasileiras ou estrangeiras: - Servier; Boerinhger; AstraZeneca; Libbs.
José Augusto Soares Barreto Filho	Nada a ser declarado
José Fernando Vilela-Martin	Nada a ser declarado
José Marcio Ribeiro	Nada a ser declarado
Juan Carlos Yugar-Toledo	Nada a ser declarado
Lucélia Batista Neves Cunha Magalhães	Outros relacionamentos Financiamento de atividades de educação médica continuada, incluindo viagens, hospedagens e inscrições para congressos e cursos, provenientes da indústria farmacêutica, de órteses, próteses, equipamentos e implantes, brasileiras ou estrangeiras: - AstraZeneca: Selozok; Farmoquímica: Exforge.
Luciano Ferreira Drager	Nada a ser declarado
Luiz Aparecido Bortolotto	Declaração financeira A - Pagamento de qualquer espécie e desde que economicamente apreciáveis, feitos a (i) você, (ii) ao seu cônjuge/ companheiro ou a qualquer outro membro que resida com você, (iii) a qualquer pessoa jurídica em que qualquer destes seja controlador, sócio, acionista ou participante, de forma direta ou indireta, recebimento por palestras, aulas, atuação como proctor de treinamentos, remunerações, honorários pagos por participações em conselhos consultivos, de investigadores, ou outros comitês, etc. Provenientes da indústria farmacêutica, de órteses, próteses, equipamentos e implantes, brasileiras ou estrangeiras: - GSK: Shingrix; Servier: Triplicam.
Marco Antonio de Melo Alves	Outros relacionamentos Financiamento de atividades de educação médica continuada, incluindo viagens, hospedagens e inscrições para congressos e cursos, provenientes da indústria farmacêutica, de órteses, próteses, equipamentos e implantes, brasileiras ou estrangeiras: - Torrent: Dislipidemia/Hipertensão.
Marco Antonio Mota-Gomes	Declaração financeira A - Pagamento de qualquer espécie e desde que economicamente apreciáveis, feitos a (i) você, (ii) ao seu cônjuge/ companheiro ou a qualquer outro membro que resida com você, (iii) a qualquer pessoa jurídica em que qualquer destes seja controlador, sócio, acionista ou participante, de forma direta ou indireta, recebimento por palestras, aulas, atuação como proctor de treinamentos, remunerações, honorários pagos por participações em conselhos consultivos, de investigadores, ou outros comitês, etc. Provenientes da indústria farmacêutica, de órteses, próteses, equipamentos e implantes, brasileiras ou estrangeiras: - Omron: Equipamentos; Biolab: Press Plus; Daiichi Sankyo: Benicar Triplo; AstraZeneca: Forxiga; Ache: Edistride. B - Financiamento de pesquisas sob sua responsabilidade direta/pessoal (direcionado ao departamento ou instituição) provenientes da indústria farmacêutica, de órteses, próteses, equipamentos e implantes, brasileiras ou estrangeiras: - Novartis: Inclisiran; Libbs: Associação Tripla; Servier: Associação Quádrupla.
Marcus Vinícius Bolívar Malachias	Declaração financeira A - Pagamento de qualquer espécie e desde que economicamente apreciáveis, feitos a (i) você, (ii) ao seu cônjuge/ companheiro ou a qualquer outro membro que resida com você, (iii) a qualquer pessoa jurídica em que qualquer destes seja controlador, sócio, acionista ou participante, de forma direta ou indireta, recebimento por palestras, aulas, atuação como proctor de treinamentos, remunerações, honorários pagos por participações em conselhos consultivos, de investigadores, ou outros comitês, etc. Provenientes da indústria farmacêutica, de órteses, próteses, equipamentos e implantes, brasileiras ou estrangeiras: - Boehringer Ingelheim/Lilly: diabetes; Daiichi Sankyo: Hipertensão; Libbs: Hipertensão, Roche: Biomarcadores; Novo Nordisk: Diabetes; Bayer: Diabetes; Novartis: Insuficiência Cardíaca; Viatris: Insuficiência Cardíaca; Servier: Insuficiência Cardíaca. Outros relacionamentos Financiamento de atividades de educação médica continuada, incluindo viagens, hospedagens e inscrições para congressos e cursos, provenientes da indústria farmacêutica, de órteses, próteses, equipamentos e implantes, brasileiras ou estrangeiras: - Boehringer Ingelheim/Lilly: Diabetes; Daiichi Sankyo: Hipertensão; Libbs: Hipertensão, Novo Nordisk: Diabetes; Bayer: Diabetes. Participação societária de qualquer natureza e qualquer valor economicamente apreciável de empresas na área de saúde, de ensino ou em empresas concorrentes ou fornecedoras da SBC: - Instituto de Hipertensão Arterial de Minas Gerais: serviços na área da saúde; Cardio Check Up: serviços na área da saúde.
Mario Fritsch Toros Neves	Nada a ser declarado
Mayara Cedrim Santos	Nada a ser declarado
Nelson Dinamarco	Nada a ser declarado
Osni Moreira Filho	Declaração financeira A - Pagamento de qualquer espécie e desde que economicamente apreciáveis, feitos a (i) você, (ii) ao seu cônjuge/ companheiro ou a qualquer outro membro que resida com você, (iii) a qualquer pessoa jurídica em que qualquer destes seja controlador, sócio, acionista ou participante, de forma direta ou indireta, recebimento por palestras, aulas, atuação como proctor de treinamentos, remunerações, honorários pagos por participações em conselhos consultivos, de investigadores, ou outros comitês, etc. Provenientes da indústria farmacêutica, de órteses, próteses, equipamentos e implantes, brasileiras ou estrangeiras: - Daiichi Sankyo: Benicar Triplo; Servier: Triplixam. Outros relacionamentos Financiamento de atividades de educação médica continuada, incluindo viagens, hospedagens e inscrições para congressos e cursos, provenientes da indústria farmacêutica, de órteses, próteses, equipamentos e implantes, brasileiras ou estrangeiras: - Torrent: Hipertensão Arterial; Servier: Hipertensão Arterial.
Oswaldo Passarelli Júnior	Nada a ser declarado
Paulo Cesar Brandão Veiga Jardim	Nada a ser declarado
Priscila Valverde de Oliveira Vitorino	Nada a ser declarado
Roberto Dischinger Miranda	Outros relacionamentos Financiamento de atividades de educação médica continuada, incluindo viagens, hospedagens e inscrições para congressos e cursos, provenientes da indústria farmacêutica, de órteses, próteses, equipamentos e implantes, brasileiras ou estrangeiras: - Novo Nordisk: Ozempic; EMS: Hipertensão Arterial e Fibrilação Atrial.
Rodrigo Bezerra	Nada a ser declarado
Rodrigo Pinto Pedrosa	Nada a ser declarado
Rogério Baumgratz de Paula	Nada a ser declarado
Rogério Toshiro Passos Okawa	Declaração financeira A - Pagamento de qualquer espécie e desde que economicamente apreciáveis, feitos a (i) você, (ii) ao seu cônjuge/ companheiro ou a qualquer outro membro que resida com você, (iii) a qualquer pessoa jurídica em que qualquer destes seja controlador, sócio, acionista ou participante, de forma direta ou indireta, recebimento por palestras, aulas, atuação como proctor de treinamentos, remunerações, honorários pagos por participações em conselhos consultivos, de investigadores, ou outros comitês, etc. Provenientes da indústria farmacêutica, de órteses, próteses, equipamentos e implantes, brasileiras ou estrangeiras: - EMS: Hipertensão. Outros relacionamentos Financiamento de atividades de educação médica continuada, incluindo viagens, hospedagens e inscrições para congressos e cursos, provenientes da indústria farmacêutica, de órteses, próteses, equipamentos e implantes, brasileiras ou estrangeiras: - Novo Nordisk: Obesidade.
Rui Manuel dos Santos Povoa	Nada a ser declarado
Sandra C. Fuchs	Nada a ser declarado
Sandro Gonçalves de Lima	Nada a ser declarado
Sayuri Inuzuka	Outros relacionamentos Financiamento de atividades de educação médica continuada, incluindo viagens, hospedagens e inscrições para congressos e cursos, provenientes da indústria farmacêutica, de órteses, próteses, equipamentos e implantes, brasileiras ou estrangeiras: - Servier; EMS; Daichi Sankyo.
Sebastião Rodrigues Ferreira-Filho	Nada a ser declarado
Silvio Hock de Paffer Fillho	Nada a ser declarado
Thiago de Souza Veiga Jardim	Nada a ser declarado
Vanildo da Silva Guimarães Neto	Nada a ser declarado
Vera Hermina Kalika Koch	Nada a ser declarado
Waléria Dantas Pereira Gusmão	Nada a ser declarado
Weimar Kunz Sebba Barroso	Declaração financeira A - Pagamento de qualquer espécie e desde que economicamente apreciáveis, feitos a (i) você, (ii) ao seu cônjuge/ companheiro ou a qualquer outro membro que resida com você, (iii) a qualquer pessoa jurídica em que qualquer destes seja controlador, sócio, acionista ou participante, de forma direta ou indireta, recebimento por palestras, aulas, atuação como proctor de treinamentos, remunerações, honorários pagos por participações em conselhos consultivos, de investigadores, ou outros comitês, etc. Provenientes da indústria farmacêutica, de órteses, próteses, equipamentos e implantes, brasileiras ou estrangeiras: - EMS; Brace Pharma; Servier; Biolab; Omron; Cardios. B - Financiamento de pesquisas sob sua responsabilidade direta/pessoal (direcionado ao departamento ou instituição) provenientes da indústria farmacêutica, de órteses, próteses, equipamentos e implantes, brasileiras ou estrangeiras: - Ministério de Saúde; PROADI SUS; Novartis: Entresto. C - Financiamento de pesquisa (pessoal), cujas receitas tenham sido provenientes da indústria farmacêutica, de órteses, próteses, equipamentos e implantes, brasileiras ou estrangeiras: - EMS: Estudo RITMO; Brace Pharma: Estudo BRACE 23. Outros relacionamentos Financiamento de atividades de educação médica continuada, incluindo viagens, hospedagens e inscrições para congressos e cursos, provenientes da indústria farmacêutica, de órteses, próteses, equipamentos e implantes, brasileiras ou estrangeiras: - Servier; Congresso Europeu de Hipertensão Arterial.
Wille Oigman	Nada a ser declarado
Wilson Nadruz Junior	Nada a ser declarado

## Sumário

Atualizações e Mudanças nas Diretrizes Brasileiras de Medidas da Pressão Arterial Dentro e Fora do Consultório 10

Parte 1 – Medidas da Pressão Arterial 10

1. Introdução 10

2. Medida da PA no Consultório 11

2.1. Medida da PA com Técnica Auscultatória 11

2.2. Medida da PA com Técnica Oscilométrica e Equipamentos Automáticos ou Semiautomáticos 11

2.3. Etapas para Medida da PA em Consultório 11

2.4. Medida da PA no Punho e no Dedo 12

2.5. Medida da PA no Idoso 13

2.6. Classificação da PA de Acordo com a Medida de Consultório 13

3. Medidas da PA Fora do Consultório 14

3.1. Automedida da Pressão Arterial (AMPA) 14

3.2. Medidas da PA em Farmácias 14

3.3. Medidas da PA em Espaços Públicos 14

3.4 Monitoração Ambulatorial da Pressão Arterial (MAPA) 15

3.5. Monitoração Residencial da Pressão Arterial (MRPA) 15

4. Medida Central da PA e seus Parâmetros Derivados 15

5. Medida da PA Durante o Exercício Físico 15

Parte 2 – Aspectos e Conceitos Comuns à MAPA e a MRPA 15

1. Aspectos Indispensáveis para a Constituição de um Serviço de MAPA e MRPA 15

2. Valores de Anormalidade para PA no Consultório e Fora do Consultório 16

2.1. Normotensão e Hipertensão Controlada 16

2.2. Hipertensão Sustentada e Hipertensão Sustentada Não Controlada 16

2.3. Hipertensão do Avental Branco e Hipertensão do Avental Branco Não Controlada 16

**2.3.1. Efeito do Avental Branco** 16

**2.3.2. Investigação** 16

**2.3.3. Prognóstico** 16

**2.3.4. Seguimento e Tratamento** 16

2.4. Hipertensão Mascarada e Hipertensão Mascarada Não Controlada 16

**2.4.1. Efeito de Mascaramento** 17

**2.4.2. Investigação** 17

**2.4.3. Prognóstico** 17

**2.4.4. Tratamento** 18

2.5 Hipertensão Resistente 18

3. Indicações, Vantagens e Desvantagens da MAPA e MRPA 18

Parte 3 – Monitoração Ambulatorial da Pressão Arterial (MAPA) 18

1. Definição 18

2. Protocolos para Realização da MAPA 19

2.1. Reprodutibilidade da MAPA 19

3. Orientações aos Pacientes e Profissionais de Saúde 20

3.1. Diário de Atividades Durante o Exame 20

3.2. Orientações Gerais 20

4. Interpretação dos Resultados 20

4.1. Critérios para Interpretação do Exame 20

4.2. Valores de Anormalidade 20

4.3. Comportamento da Pressão Arterial entre os Períodos de Vigília e Sono 20

4.4. Ascensão Matinal da Pressão Arterial 20

4.5. Cargas de Pressão, Áreas sob as Curvas, Variabilidade da Pressão Arterial e Frequência Cardíaca 20

**4.5.1. Cargas de Pressão** 20

**4.5.2. Áreas sob as Curvas** 21

**4.5.3. Variabilidade da Pressão Arterial** 21

**4.5.4. Frequência Cardíaca** 23

5. Interpretação da MAPA e Emissão de Laudo 23

5.1. Emissão de Laudo 23

5.2. Comportamento da Pressão Arterial Sistólica e Diastólica nas 24 Horas, Vigília e Sono 23

5.3. Comportamento da Pressão Arterial Sistólica e Diastólica entre os Períodos de Vigília e Sono 23

5.4. Pico de Pressão e Hipotensão 24

5.5. Correlação entre Atividades, Medicamentos Utilizados e Sintomas 24

5.6. Conclusão 24

6. Aplicações Clínicas da MAPA 25

6.1. Para Avaliação do Prognóstico de Pacientes com Hipertensão Arterial 25

6.2. Para Avaliação da Eficácia Terapêutica Anti-hipertensiva 25

7. MAPA em Situações Especiais 25

7.1. Crianças e Adolescentes 25

7.2. Gestantes 26

7.3. Idosos 26

7.4. Diabetes Melito 26

7.5. Doença Renal Crônica 26

7.6. Síndrome da Apneia Obstrutiva do Sono 27

7.7 Insuficiência Cardíaca 27

7.8. Exercício Físico 27

8. Custo-efetividade 27

9. Perspectivas 28

Parte 4 – Monitoração Residencial da Pressão Arterial 28

1. Introdução 28

2. Orientações ao Paciente 28

3. Protocolo para Realização do Exame 28

4. Valores Referenciais de Anormalidade 29

5. Emissão de Laudo e Interpretação dos Resultados 29

6. Aplicações da Monitoração Residencial da Pressão Arterial 29

6.1. Para o estabelecimento do comportamento da pressão arterial no consultório e fora dele 29

6.2. Para a Avaliação do Prognóstico 31

6.3. Para Avaliação da Terapêutica Anti-hipertensiva 31

6.4. Em Situações e Populações Especiais 32

**6.4.1. Crianças e Adolescentes** 32

**6.4.2. Gestantes** 32

**6.4.3. Idosos** 32

**6.4.4. Diabetes Melito** 32

**6.4.5. Doença Renal Crônica** 32

**6.4.6. Obesidade** 32

**6.4.7. Nas Arritmias** 33

7. Custo-efetividade 33

8. Perspectivas 33

Parte 5 – Pressão Central, Velocidade da Onda de Pulso e *Augmentation Index* 33

1. Introdução 33

2. Definições 33

2.1. Velocidade de Onda de Pulso 33

2.2. Augmentation Index 33

2.3. Pressão Central 34

3. Indicações 34

4. Vantagens da Medida da Pressão Central, *Augmentation Index* e Velocidade da Onda de Pulso 34

5. Limitações da Medida da Pressão Central, *Augmentation Index* e Velocidade da Onda de Pulso 34

6. Técnicas Disponíveis para Verificação dos Parâmetros Centrais e de Rigidez Arterial 34

6.1. Métodos para Medida Indireta da Pressão Arterial Central 34

7. Protocolos para Medidas de PAC, VOP e AIx 34

7.1. Protocolo para Realização das Medidas de Parâmetros Centrais pelo Método Tonométrico 34

7.2. Protocolo para Realização do Triplo Tiro (Medida do Consultório) pelo Método Oscilométrico 35

7.3. Protocolo para Realização das Medidas de parâmetros Centrais da PA de 24 Horas pelo Método Oscilométrico 35

8. Valores de Referência 35

8.1 Valores de Referência para Velocidade de Onda de Pulso (VOP) 35

8.2. Valores de Referência para a Pressão Sistólica Central (PSc) 36

8.3. Valores de Referência para VOP, PAC e AIx Utilizando o Método Oscilométrico na População Brasileira 36

9. Valor Prognóstico das Medidas Derivadas dos Parâmetros Centrais 38

10. Parâmetros Centrais de 24 Horas 38

11. Perpectivas 39

Referências 40

## Atualizações e Mudanças nas Diretrizes Brasileiras de Medidas da Pressão Arterial Dentro e Fora do Consultório

Estas diretrizes, revisando e atualizando as anteriores de Monitorização Ambulatorial da Pressão Arterial (MAPA) e Monitorização Residencial da Pressão Arterial (MRPA), introduzem uma série de modificações relevantes, destacando aspectos fundamentais na avaliação da medida e monitorização da Pressão Arterial (PA) tanto no ambiente de consultório quanto fora dele, incluindo MAPA, MRPA, Automedida da Pressão Arterial (AMPA) e Avaliação da Pressão Arterial Central. Algumas das principais alterações incluem:

1. A atualização da 6ª Diretriz Brasileira de MAPA e 4ª Diretriz Brasileira de MRPA, publicada em 2018, passou a ser denominadas “Diretrizes Brasileiras de Medidas da Pressão Arterial Dentro e Fora do Consultório”. Essa nova nomenclatura reflete a abrangência e atualidade das diretrizes, destacando seu impacto na prática clínica.
2. Ênfase na precisão e qualidade das medidas de PA em consultório.
3. Novas orientações sobre a medida da PA desacompanhada no consultório.
4. Avaliação rigorosa da hipotensão postural.
5. Uso expandido das medidas de PA em farmácias e espaços públicos para rastreio da hipertensão.
6. Atualização das indicações clínicas para medidas de PA, tanto em consultório como fora dele.
7. Reconhecimento da importância das medidas de PA durante o exercício físico.
8. Certificação e validação necessárias para monitores (Consultar www.stridebp.org e INMETRO).
9. Valores atualizados dos critérios de normalidade da PA na MRPA.
10. Novos comportamentos identificados em indivíduos sob tratamento medicamentoso.
11. Fluxograma revisado para avaliação e conduta em hipertensão mascarada e do avental branco.
12. Atualização das indicações, limitações, vantagens e desvantagens da MAPA e MRPA.
13. Nova classificação de valores de normalidade da PA obtida pela MAPA em crianças e adolescentes.
14. Atualização do protocolo para realização de MRPA.
15. Atualização dos valores referentes aos efeitos do avental branco e mascaramento pela MRPA.
16. Novo protocolo de MRPA para pacientes em hemodiálise.
17. Introdução de um Capítulo sobre Pressão Arterial Central (PAC), Velocidade de Onda de Pulso (VOP) e Augmentation Index (AIx), o qual inclui possíveis indicações, definição de protocolos específicos e valores de referência para as medidas de VOP, PAC e Aix.

Essas modificações reforçam o compromisso com a precisão diagnóstica e aprimoramento do cuidado relacionado à pressão arterial, promovendo práticas clínicas embasadas e alinhadas com os mais recentes avanços na área.

## Parte 1 – Medidas da Pressão Arterial

## 1. Introdução

A hipertensão arterial (HA) é um dos principais fatores de risco modificáveis para morbidade e mortalidade em todo o mundo, sendo um dos maiores fatores de risco para doença arterial coronária, acidente vascular cerebral (AVC) e insuficiência renal. Além disso, é altamente prevalente e atinge mais de um terço da população mundial.^1 - 9^

A medida da PA é procedimento OBRIGATÓRIO em qualquer atendimento médico ou realizado por diferentes profissionais de saúde. Contudo, ainda é comumente realizada sem os cuidados técnicos necessários.^1 - 9^Como o diagnóstico se baseia na medida da PA, fica claro o cuidado que deve haver com as técnicas, os métodos e os equipamentos utilizados na sua realização.^1 - 9^

Deve-se reforçar que, feito o diagnóstico, toda a investigação e os tratamentos de curto, médio e longo prazos são feitos com base nos resultados da medida da PA. Assim, técnicas e/ou equipamentos inadequados podem levar a diagnósticos incorretos, tanto subestimando quanto superestimando valores e levando a condutas inadequadas e grandes prejuízos à saúde e à economia das pessoas e das nações.^1 - 11^

Uma vez feito o diagnóstico correto, na medida em que avança o conhecimento da importância do tratamento adequado, com a adoção de valores de normalidade mais detalhados e com objetivos de tratamento mais cuidadosos no sentido do alcance de metas de PA mais rigorosas, fica também reforçada a importância da precisão na medida da PA.^1 - 11^

A medida da PA (descrita a seguir) é habitualmente feita pelo método tradicional, a assim chamada medida casual ou de consultório. Ao longo do tempo, foram agregadas alternativas a ela, mediante o uso de equipamentos semiautomáticos ou automáticos pelo próprio paciente, nas salas de espera ou fora do consultório, em sua própria residência ou em espaços públicos. Um passo adiante foi dado com o uso de equipamentos semiautomáticos providos de memória que permitem medidas sequenciais fora do consultório (AMPA; ou MRPA) e outros automáticos que permitem medidas programadas por períodos mais prolongados (MAPA).^1 , 2 , 4 , 12 , 13^

Alguns aspectos na medida da PA podem interferir na obtenção de resultados fidedignos e, consequentemente, causar prejuízo nas condutas a serem tomadas. Entre eles, estão: a importância de serem utilizados valores médios, a variação da PA durante o dia e a variabilidade a curto prazo. Esses aspectos têm estimulado a realização de maior número de medidas em diversas situações, e as diferentes diretrizes têm preconizado o uso de equipamentos que favoreçam essas ações. Ganham cada vez mais espaço os equipamentos que realizam MRPA ou MAPA, que, além de permitirem maior precisão, se empregados em conjunto, detectam a HA do avental branco (HAB), HA mascarada (HM), alterações da PA no sono e HA resistente (HAR) (definidos no Capítulo 2 desta diretriz).^1 - 9 , 14 - 20^

Resguardados esses detalhes, devemos ressaltar que as informações relacionadas a diagnóstico, classificação e estabelecimento de metas ainda são baseadas na medida da PA de consultório e, por esse motivo, toda a atenção deve ser dada à realização desse procedimento.^15 , 16 , 21^

## 2. Medida da PA no Consultório

### 2.1. Medida da PA com Técnica Auscultatória 2 , 12 , 22 - 25

- O esfigmomanômetro de mercúrio é considerado o dispositivo padrão de referência e pode ser útil para validar dispositivos oscilométricos e aneroides, embora tenha sido abolido da área da saúde por lei do Ministério do Trabalho – (NR 15 125.001-9/I4).
- Em muitos locais, os manômetros aneroides substituíram os dispositivos de mercúrio. Porém, são facilmente danificados e requerem recalibração frequente, ao menos a cada 12 meses, para garantir sua precisão.
- A medida da PA com técnica auscultatória ainda é amplamente realizada em nosso meio com esfigmomanômetro aneroide e estetoscópio.
- O esfigmomanômetro é formado pelo conjunto do manguito, a bolsa de borracha inflável revestida por tecido não distensível; manômetro para registro da pressão; e sistema de válvulas, tubos e pera de borracha que permite a inflação e a deflação.
- A inflação do manguito sobre a artéria braquial interrompe o fluxo sanguíneo. Com a deflação, há redução da pressão do manguito; quando a pressão gerada pela contração do ventrículo esquerdo impulsiona o sangue pela artéria, há a produção de sons característicos que são auscultados pelo estetoscópio. São os chamados sons de Korotkoff (Quadro 1).
- A técnica auscultatória requer boa audição coordenada com a visualização dos valores na escala do aparelho, para promover a identificação correta dos sons que determinam as pressões arteriais sistólica e diastólica.

### 2.2. Medida da PA com Técnica Oscilométrica e Equipamentos Automáticos ou Semiautomáticos 4 , 13 , 24 - 27

- A medida da PA com aparelhos automáticos ou semiautomáticos, com técnica oscilométrica, apresenta vantagens em relação à técnica auscultatória, principalmente por afastar ou diminuir os erros sistemáticos de aproximação de valores e da influência da presença do observador, além da realização incorreta do procedimento (Quadro 2).
- Outra utilidade do uso desses equipamentos é a realização da medida da PA pelo paciente sem a presença do profissional de saúde, denominada medida da PA desacompanhada no consultório (Quadro 3). Essa estratégia incrementa a reprodutibilidade da medida, além de minimizar o efeito do avental branco.
- Os equipamentos automáticos ou semiautomáticos de medida da PA devem ser validados por protocolos específicos, e a lista de aparelhos validados pode ser encontrada nos seguintes *links* : *British and Irish Hypertension Society* (www.bihsoc.org/bp-monitors); *European Society of Hypertension – International Society of Hypertension – World Hypertension League* (www.stridebp.org); e *Hypertension Canada* (www.hypertension.ca/bpdevices). A maioria dos equipamentos disponíveis no mercado não foi validada.
- Os equipamentos, mesmo validados para adultos, necessitam de validação em populações especiais: crianças, gestantes, idosos, pessoas com circunferência do braço acima de 42 cm e aqueles com arritmias.
- A avaliação da calibração dos equipamentos automáticos ou semiautomáticos deve ser realizada a cada 12 meses.
- Os manguitos de diferentes dimensões devem ser do mesmo fabricante e modelo do aparelho.

### 2.3. Etapas para Medida da PA em Consultório 1 , 10 , 11 , 16 , 28 , 29

Na medida da PA, seja com técnica auscultatória ou oscilométrica, cuidados importantes devem ser tomados visando ao adequado preparo do paciente e à realização do procedimento, conforme descrito no Quadro 4 .

**Quadro 4:** – Etapas para a medida da pressão arterial (PA) em consultório

Preparo do paciente
Repouso por 5 minutos, em ambiente calmo e confortável, e orientar para não falar ou se mover durante a medida
Verificar se o paciente NÃO: •Está com a bexiga cheia •Praticou exercícios físicos há, pelo menos, 90 minutos •Ingeriu bebidas alcoólicas, café, alimentos ou fumou 30 minutos antes
Sentar o paciente, com pernas descruzadas, pés apoiados no chão, dorso relaxado e recostado na cadeira
Posicionar o braço na altura do coração, apoiado, com a palma da mão voltada para cima e sem garrotear o braço com roupas
Na primeira consulta, registrar a PA em ambos os braços, preferencialmente de forma simultânea, e usar a leitura do braço que forneceu valor mais elevado para medidas subsequentes. Registrar em que braço devem ser feitas as medidas
**Etapas da medida**
Colocar o manguito, sem deixar folgas, 2 a 3 cm acima da fossa cubital, centralizar o meio da bolsa inflável sobre a artéria braquial
Estimar o nível da PA sistólica* (Quadro 5)
Palpar a artéria braquial na fossa cubital e colocar a campânula ou o diafragma do estetoscópio sem compressão excessiva.* Não permitir que o estetoscópio seja colocado sob o manguito
Inflar rapidamente até ultrapassar 20 a 30 mmHg o nível estimado da PA sistólica*
Realizar a deflação lentamente (cerca de 2 mmHg/segundo)*
Determinar a PA sistólica na ausculta do primeiro som (fase I de Korotkoff)
Determinar a PA diastólica no desaparecimento dos sons (fase V de Korotkoff)*
Continuar a auscultar cerca de 20 a 30 mmHg abaixo do último som para confirmar seu desaparecimento e depois proceder a deflação rápida e completa. Se os batimentos persistirem até o nível zero, determinar a diastólica no abafamento dos sons (fase IV de Korotkoff) e anotar valores da PA sistólica/diastólica/zero*
Realizar três medidas, com intervalo de 1 minuto, e usar a média das duas últimas medidas. Se houver diferença > 10 mmHg, realizar medidas adicionais.

*Aplicados à técnica auscultatória.

A análise da hipotensão postural é uma etapa importante na avaliação do paciente, principalmente em idosos, indivíduos com disautonomia e nos pacientes em uso de medicações anti-hipertensivas. Para pesquisá-la, meça a PA na posição supina (após o paciente estar nessa posição por 5 minutos) e depois meça a PA, 1 e 3 minutos após a pessoa ficar em pé. A medida da PA feita em pé deve ser feita preferencialmente com o braço do paciente apoiado pelo examinador no nível do coração. A hipotensão postural é definida como uma redução ≥ 20 mmHg para PA sistólica ou ≥ 10 mmHg para a PA diastólica no primeiro e/ou terceiro minuto na posição ortostática em relação à posição supina.

### 2.4. Medida da PA no Punho e no Dedo

Como descrito, os equipamentos mais utilizados e padronizados para medir a PA utilizam o manguito envolvendo o braço e o método de oclusão-liberação do manguito, associado à técnica auscultatória ou à análise pelo método oscilométrico (aparelhos semiautomáticos e automáticos). Entretanto, existem equipamentos que contêm manguito para envolver a região do punho, associado também à técnica oscilométrica (aparelhos automáticos). Os denominados “monitores de pulso” têm a vantagem de ser menores e de mais fácil transporte e, de fato, vêm sendo utilizados amplamente por pacientes para monitoração da PA fora do consultório. Uma indicação desses modelos é para indivíduos muito obesos, em que os erros nas medidas realizadas nos braços são mais frequentes e pode haver dificuldade com largura e comprimento de manguitos. No entanto, as medidas da PA feitas com esses equipamentos estão sujeitas a apresentar maior número de erros sistemáticos. A posição do punho (altura) em relação ao coração no momento da medida e o aparecimento frequente de artefatos tornam a medida menos fidedigna. Esses aparelhos têm sido aperfeiçoados para liberação de medidas apenas quando a posição é adequada em relação ao coração, entretanto, ainda faltam estudos de validação e correlação mais consistentes.^30 - 32^

Recentemente, novas tecnologias para medir a PA sem a utilização de manguitos vêm sendo desenvolvidas. Dispositivos denominados *wearables* (vestíveis, utilizáveis), que podem ser acoplados a pulseiras ou relógios, medem a PA por meio de diferentes tecnologias, como fotopletismografia e tonometria de aplanação. Apesar de não existirem estudos de validação dos dispositivos frente às formas clássicas de medida da PA, muitos já estão no mercado. Pelo grande apelo promocional como: facilidade de uso, monitoração praticamente contínua da PA, alguns sem necessidade de calibração, facilidade de compartilhamento dos dados e transmissão dos valores obtidos quase em tempo real, esses novos dispositivos têm atraído significativo número de usuários. Contudo, não há recomendação para o uso dessas novas tecnologias e dispositivos no acompanhamento de pacientes na prática clínica atual. Aguardaremos a realização e a divulgação de estudos clínicos que forneçam embasamento científico para que tal acompanhamento possa ser feito.^30 - 32^ Os monitores de dedo ainda são imprecisos e não devem ser utilizados.

### 2.5. Medida da PA no Idoso 33 - 35

- A medida da PA no idoso pode sofrer influência do processo de envelhecimento e as mais comuns são: pseudo-hipertensão, hiato auscultatório, arritmias e hipotensão postural.
- A pseudo-hipertensão é considerada quando há valor indevidamente elevado da PA medida pelo método indireto, comparada com o método direto, em consequência do processo de ateromatose excessiva associada ou não à hipertrofia da camada média das artérias. Nesses casos, a calcificação da parede arterial promove um enrijecimento tão pronunciado que a insuflação do manguito é insuficiente para colabar a artéria braquial. Além disso, deve ser considerado que esse diagnóstico é sugerido quando a PA sistólica se apresenta muito elevada sem acometimento de órgãos-alvo ou quando o paciente apresenta manifestações de hipotensão. A suspeita dessa situação pode ser reforçada pelo achado de cálcio em exame radiológico.
- A manobra de Osler pode ser útil na avaliação da pseudo-hipertensão e consiste na insuflação do manguito no braço até o desaparecimento do pulso radial. Se o trajeto da artéria for palpável após esse procedimento, o paciente é considerado Osler positivo.
- O hiato auscultatório caracteriza-se pelo desaparecimento da ausculta durante a deflação, entre o final da fase I e o início da fase II dos sons de Korotkoff. Dessa forma, pode haver subestimação da PA sistólica ou superestimação da PA diastólica. A estimação do nível da PA sistólica pela medida palpatória é imprescindível para identificação e correção da medida nessa condição.
- A hipotensão postural ou ortostática é frequente nos idosos que apresentam sintomas como tontura, visão turva, escotomas, astenia ou síncope, na mudança da posição supina para a posição ereta. Recomenda-se a verificação da PA no idoso na posição sentada, deitada e em pé, pois as alterações ateroscleróticas nas regiões dos seios carotídeos podem reduzir a sensibilidade dos barorreceptores, ocasionando maior variabilidade da PA nos idosos e redução dos reflexos posturais, o que predispõe à hipotensão postural. O uso de fármacos como diuréticos, antidepressivos, vasodilatadores e betabloqueadores pode também ocasionar hipotensão postural. A prevalência da hipotensão postural em idosos com mais de 75 anos tem sido referida como 34%, e assume importância clínica quando se manifesta com tontura postural, sobretudo na vigência de uso de fármacos hipotensores.
- A presença de arritmias, como fibrilação atrial e extrassistolia, pode dificultar a medida da PA com aparelhos que usam a técnica oscilométrica, exceto nos aparelhos que têm mecanismo de captação da fibrilação atrial e outras arritmias.

### 2.6. Classificação da PA de Acordo com a Medida de Consultório

A classificação é definida de acordo com a PA no consultório e pelo nível mais elevado da sistólica ou diastólica. Considera-se hipertensão sistólica isolada se PAS ≥ 140 mmHg e PAD < 90 mmHg, e deve ser classificada em estágios 1, 2 e 3 ( Quadro 8 ).

**Quadro 8:** – Classificação da pressão arterial (PA) de acordo com a medida no consultório a partir de 18 anos de idade (GR: I – NE: C)

Classificação	PA sistólica (mmHg)	PA diastólica (mmHg)
Ótima	< 120 e	< 80
Normal	120-129 e/ou	80-84
Pré-hipertensão	130-139 e/ou	85-89
Hipertensão estágio 1	140-159 e/ou	90-99
Hipertensão estágio 2	160-179 e/ou	100-109
Hipertensão estágio 3	≥ 180 e/ou	≥ 110

Adaptado das Diretrizes Brasileiras de Hipertensão Arterial (2020).^1^ GR: grau de recomendação; NE: nível de evidência.

É importante salientar que o reconhecimento da HA – portanto, o diagnóstico final – não deve se embasar em uma única medida da PA, considerando-se que ela pode ser muito variável. Assim, níveis pressóricos que não se enquadrem em estágio 3 em ambiente de consultório devem ser reavaliados em medidas subsequentes para a confirmação diagnóstica, bem como para a definição do estágio de hipertensão.

Várias medidas devem ser realizadas em diferentes dias no consultório médico, observando-se 1 a 2 minutos de intervalo entre as medidas.^36 - 39^

Ainda assim, com alguma frequência, os valores obtidos nos consultórios (medida casual) não são suficientes para a caracterização da HA. As medidas de consultório estão sujeitas a inúmeros vieses (erros sistemáticos), além de proporcionarem um número reduzido de medidas. Nos casos de dúvidas, a utilização de outros métodos de medida para o diagnóstico e seguimento são necessários, citados neste capítulo e detalhados em capítulos subsequentes.

## 3. Medidas da PA Fora do Consultório

### 3.1. Automedida da Pressão Arterial (AMPA)

De acordo com a 6ª Diretriz de MAPA e a 4ª Diretriz de MRPA, a AMPA é a medida da PA realizada pelo próprio paciente ou familiar em seu domicílio. Ainda, segundo as referidas diretrizes, a AMPA não obedece a nenhum protocolo preestabelecido, sendo as medidas realizadas aleatoriamente e feitas por decisão do próprio paciente ou até a pedido médico.^18^ Diferentemente da MAPA e da MRPA, realizadas com equipamentos automáticos com acurácia e reprodutibilidade validadas, e pertencentes às instituições de saúde, a AMPA é realizada com equipamento automático do próprio paciente.^17^

Na Diretriz Brasileira de Hipertensão Arterial 2020, um avanço substancial ocorreu no conceito AMPA ao se sugerir um número mínimo de 7 medidas realizadas no período de 16-72 horas, utilizando equipamentos oscilométricos de boa qualidade e validados. Entretanto, estudos que validem os valores da PA obtidos pela AMPA no tocante ao risco cardiovascular e renal não são conhecidos. Nem tampouco as consequências não intencionais de medidas aleatórias da PA, sem rigor metodológico, na tomada de decisão.^1^

Considerando que a acurácia da medida da PA é fundamental na tomada de decisão, a presente diretriz sugere que os mesmos cuidados e procedimentos para a medida da PA (descritos no Quadro 4 ) sejam seguidos para a AMPA. Ressalta-se, no entanto, que não há evidências conclusivas que respaldem a adoção de protocolos específicos (número de medidas, horários e dias de monitoração), nem a determinação de valores de normalidade para esse método. Dessa forma, a diretriz recomenda que a AMPA seja utilizada apenas como meio de triagem, para que métodos confirmatórios sejam solicitados (MRPA ou MAPA), se necessário ( Quadro 9 ).

**Quadro 9:** – Indicação clínica das medidas da PA no consultório e fora dele (GR: I – NE: C)

Uso clínico	Consultório	MRPA	MAPA	AMPA	Farmácia	Espaços públicos
Triagem	+++	+	_	++	++	+
Diagnóstico inicial	+	++	+++	-	-	-
Ajuste de dose	+	++	++	+ (?)	-	-
Seguimento	++	+++	+	+ (?)	+ (?)	-
Indicação principal	Triagem de seguimento	Seguimento	Diagnóstico inicial	Triagem	Triagem de seguimento (?)	Triagem de oportunidade
Valores, mmHg	≥ 140 × 90	≥ 130 × 80	≥ 130 × 80	?	?	?

Adaptado de Stergiou et al. ^5^ GR: grau de recomendação; NE: nível de evidência; PA: pressão arterial; MAPA: monitoração ambulatorial da pressão arterial; MRPA: monitoração residencial da pressão arterial; AMPA: automedida da pressão arterial.

### 3.2. Medidas da PA em Farmácias

Esta prática é utilizada no Brasil e em vários outros países. Pelo baixo custo ao indivíduo, tem potencial para rastreamento e até seguimento de tratamento, mas carece do estabelecimento de protocolos de medida que assegurem validação dos equipamentos e dos próprios métodos de medida, além de estudos que comprovem sua validade ( Quadro 9 ). Não há critérios definidos para valores anormais.^1 , 5^

### 3.3. Medidas da PA em Espaços Públicos

São frequentemente utilizadas em campanhas educativas e apresentam como principal vantagem a possibilidade de rastrear possíveis indivíduos com HA na população geral para posterior confirmação diagnóstica em consultório ( Quadro 9 ). Não há critérios definidos para valores anormais.^1 , 4^ Recomenda-se, portanto, que, para medidas em espaços públicos, pessoas que não conheçam a condição de hipertensas e assim se apresentam, ou que estejam fora da meta pressórica e saibam ser hipertensas, sejam encaminhadas para seguimento médico.

### 3.4. Monitoração Ambulatorial da Pressão Arterial (MAPA)

Ver Capítulos 2 e 3.

### 3.5. Monitoração Residencial da Pressão Arterial (MRPA)

Ver Capítulos 2 e 4.

## 4. Medida Central da PA e seus Parâmetros Derivados

Ver Capítulo 5.

## 5. Medida da PA Durante o Exercício Físico

Recomenda-se que a PA seja medida antes e durante a execução dos exercícios em hipertensos mal controlados e/ou com resposta hiper-reativa ao exercício.^1 , 40^

A medida da PA antes da execução do exercício deve ser realizada na posição sentada ou em pé, de acordo com o exercício que será realizado, podendo-se utilizar os métodos auscultatório ou oscilométrico, seguindo-se as recomendações já descritas neste capítulo em relação à posição do corpo e do braço, tempo de repouso prévio, tamanho do manguito e técnica de medida.^2 , 5^ Recomenda-se que o exercício só iniciado somente se os valores da PA sistólica/diastólica estiverem iguais ou menores que 160/105 mmHg.^1 , 40^

A medida da PA durante a realização dos exercícios aeróbicos pode ser limitada ou impossibilitada em algumas modalidades. A medida só é precisa em modalidades que possibilitem a manutenção do braço em posição estável e no nível do coração. A medida deve, idealmente, ser realizada em momentos de equilíbrio metabólico durante o esforço, ou seja, com a intensidade mantida há, pelo menos, 3 minutos. A medida deve empregar o método auscultatório, pois os monitores automáticos validados para essa condição são raros.^41^Considera-se necessário reduzir a intensidade do exercício se os valores de PA sistólica/diastólica durante o exercício aeróbico estiverem maiores que 180/105 mmHg.^1 , 40^

Nos testes ergométricos, devido ao aumento progressivo da intensidade em intervalos variados, a medida da PA deve ser feita conforme preconizado em cada protocolo.^40 , 41^

Durante a execução dos exercícios resistidos dinâmicos, não se recomenda a medida da PA, visto que os métodos existentes para essa medida na prática clínica (auscultatório e oscilométrico) não são validados para a situação de exercício, resultando em valores imprecisos.^41^

## Parte 2 – Aspectos e Conceitos Comuns à MAPA e à MRPA

## 1. Aspectos Indispensáveis para a Constituição de um Serviço de Mapa e MRPA

Para criar ou dar continuidade a um serviço de MAPA e MRPA, independentemente de sua gestão ou localização, devem ser atendidos alguns princípios básicos, como definidos no Quadro 10 .

**Quadro 10:** – Condições indispensáveis para criação de serviços de MAPA e/ou MRPA

Médico responsável familiarizado com o método e detentor de conhecimento técnico e científico sobre o exame
Equipe formada por técnico capacitado a fazer a instalação e promover as devidas e necessárias orientações ao paciente
Equipamento oscilométrico automático de braço, validado, calibrado, com memória; manguito com todos os tamanhos; pilhas carregadas
Elaboração de laudo padronizado em planilhas, softwares ou plataformas online
Local apropriado

MAPA: monitoração ambulatorial da pressão arterial; MRPA: monitoração residencial da pressão arterial.

Os equipamentos de MAPA e/ou de MRPA deverão ser automáticos, digitais e que utilizem técnica oscilométrica validada, com armazenamento dos dados que possibilite a emissão do laudo. Esses monitores devem possuir certificado de validação (consultar o site www.stridebp.org), corroborado pelo Instituto Nacional de Metrologia, Qualidade e Tecnologia (INMETRO).^27 , 42^ Para ambos os métodos, recomendam-se os monitores de braço. Excepcionalmente na MRPA, nos pacientes com braços com diâmetros superiores a 42 cm, pode ser considerado o uso de equipamentos de punho validados ou dar preferência ao uso de MAPA com o manguito de braço adequado, de 42 a 50 cm. Além disso, os monitores devem ter sua calibração verificada, no mínimo, a cada 12 meses, ou sempre que houver suspeita de descalibração.

## 2. Valores De Anormalidade para PA no Consultório e Fora do Consultório

**Figura 1:**
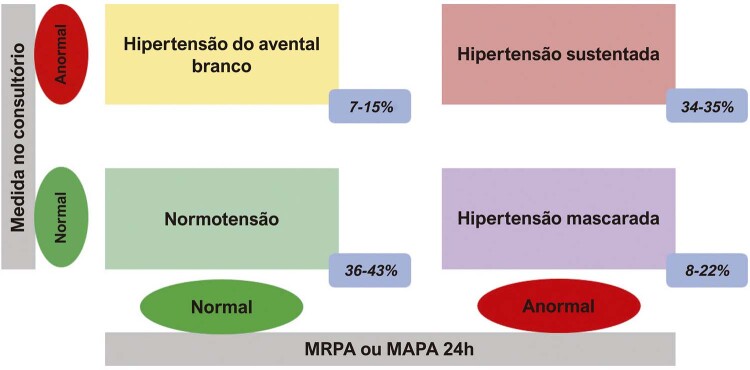
– Comportamento da PA e suas prevalências em indivíduos não tratados com medicações anti-hipertensivas. 43 , 45 MAPA: monitoração ambulatorial da pressão arterial; MRPA: monitoração residencial da pressão arterial; PA: pressão arterial.

Os valores de PA considerados anormais estão apresentados na Tabela 1 . Com o advento da medida da PA fora do consultório, foram definidos oito tipos de comportamento da PA: normotensão (NT), hipertensão controlada (HC), hipertensão sustentada (HS), hipertensão sustentada não controlada (HSNC), hipertensão do avental branco (HAB), hipertensão do avental branco não controlada (HABNC), hipertensão mascarada (HM) e hipertensão mascarada não controlada (HMNC) (Figuras 1 e 2).^1 , 5 , 43^Com o uso da MAPA, temos o nono comportamento, a hipertensão noturna.

**Tabela 1:** – Valores de PA considerados anormais nas medidas casuais (consultório), pela MAPA (nas 24 horas, vigília e durante o sono) e na MRPA para definição de diagnósticos (GR: I – NE: B) 1 **,** 18 **,** 43

	PAS (mmHg)		PAD (mmHg)
Consultório	≥ 140	e/ou	≥ 90
MAPA 24 horas	≥ 130	e/ou	≥ 80
MAPA Vigília	≥ 135	e/ou	≥ 85
MAPA Sono	≥ 120	e/ou	≥ 70
MRPA – MAPA 5d*	≥ 130	e/ou	≥ 80

GR: grau de recomendação; NE: nível de evidência; PAS: pressão arterial sistólica; PAD: pressão arterial diastólica; MAPA: monitoração ambulatorial da pressão arterial; MRPA: monitoração residencial da pressão arterial. *Em 8 de maio de 2019, foi introduzida na Classificação Brasileira Hierarquizada de Procedimentos Médicos (CBHPM) a MRPA, com a denominação de Monitoração Ambulatorial da Pressão Arterial de 5 dias (MAPA 5d) e código: 2.01.02.16-0. ^44^

### 2.1. Normotensão e Hipertensão Controlada

Define-se normotensão quando os valores da PA medida no consultório são inferiores a 140/90 mmHg e médias de PA obtidas pela MAPA 24 horas ou pela MRPA inferiores a 130/80 mmHg, na ausência do uso de medicamentos anti-hipertensivos. Caso o indivíduo faça uso de medicação anti-hipertensiva, ele é definido como indivíduo com HC.^5 , 46^

### 2.2. Hipertensão Sustentada e Hipertensão Sustentada Não Controlada

HS é definida quando os valores de PA são anormais pelas medidas de PA no consultório (≥ 140/90 mmHg) e médias de PA anormais pela MAPA 24 horas ou MRPA (≥ 130/80 mmHg). Caso o indivíduo faça uso de medicamento anti-hipertensivo, é definido como indivíduo com HSNC.^5 , 46^

### 2.3. Hipertensão do Avental Branco e Hipertensão do Avental Branco Não Controlada

A HAB se caracteriza por valores anormais na medida da PA no consultório (≥ 140/90 mmHg) e normais de PA pela MAPA 24 horas (< 130/80 mmHg) ou pela MRPA (< 130/80 mmHg) em indivíduos sem tratamento. Quando esse comportamento ocorre em pacientes sob tratamento, denomina-se HABNC.

#### 2.3.1. Efeito do Avental Branco

O efeito do avental branco se caracteriza por exacerbação da PA na presença do médico que pode ocorrer na faixa de normotensão ou hipertensão. A presença desse efeito não muda o diagnóstico do comportamento de HA do paciente.^47 - 49^Na MAPA, tem sido considerado, empiricamente, efeito do avental branco significativo quando ≥ 20 mmHg para a PA sistólica e/ou ≥ 10 mmHg para a PA diastólica. Na MRPA, o efeito do avental branco significativo, também denominado reação de alarme, deve ser considerado quando ≥ 15 mmHg para a PAS e/ou ≥ 9 mmHg para a PA diastólica.^50^

#### 2.3.2. Investigação

Como não existem dados patognomônicos, as características que orientam a pesquisa do diagnóstico de HAB são: hipertensão sistólica ou diastólica isolada no consultório, idosos, mulheres, gestantes, não fumantes, indivíduos sem lesão de órgão-alvo e, principalmente, pacientes com diagnóstico de hipertensão estágio 1.^50 - 53^

#### 2.3.3. Prognóstico

O risco atribuído aos pacientes que têm HAB tem sido muito discutido. Alguns estudos apontam que os pacientes que têm esse diagnóstico apresentam risco cardiovascular intermediário entre NT e HS, porém mais próximo ao risco atribuído aos normotensos.^54 - 56^ Por outro lado, pacientes com HABNC não parecem ter maior risco cardiovascular do que aqueles com HC.^57^

#### 2.3.4. Seguimento e Tratamento

Nos pacientes com HAB, existe aumento do risco para se transformar em HS quando comparados aos normotensos. Assim, recomenda-se que o diagnóstico de HAB seja confirmado e os pacientes sejam seguidos anualmente, ou mais frequentemente naqueles com risco alto, por meio de MAPA e/ou MRPA para detectar progressão para HS, visto que eles têm maior probabilidade de se tornarem hipertensos.^54^ Não existem evidências de benefícios de intervenções medicamentosas, e o tratamento não medicamentoso deve ser considerado para todos os pacientes com HAB.^49^

## 2.4. Hipertensão Mascarada e Hipertensão Mascarada Não Controlada

A HM se caracteriza por valores normais de PA no consultório (< 140/90 mmHg) e anormais de PA pela MAPA durante o período de 24 horas (≥ 130/80 mmHg), ou pela MRPA (≥ 130/80 mmHg). Quando esse comportamento ocorre em pacientes sob tratamento anti-hipertensivo, denomina-se HMNC. É importante enfatizar que, na HM, ocorre mudança de diagnóstico de normotensão no consultório para HA fora dele.

### 2.4.1. Efeito de Mascaramento

O efeito do mascaramento significativo, ou reação de mascaramento, é definido pela diferença ≤ -1 mmHg entre a média da PA sistólica e/ou PA diastólica obtida na clínica e a média da MRPA. Contudo, a presença desse efeito não muda o diagnóstico do comportamento de HA do paciente.^18 , 58 , 59^

### 2.4.2. Investigação

As características que podem sugerir o diagnóstico de HM e que merecem investigação são: relatos de medidas de PA elevadas fora do consultório, indivíduos com pré-hipertensão no consultório, com lesão de órgão-alvo (hipertrofia de ventrículo esquerdo, retinopatia hipertensiva, microalbuminúria, alteração da função renal), ou com risco cardiovascular elevado (incluindo diabéticos, e com doença renal crônica).^52^ Indivíduos com pré-hipertensão têm três vezes mais risco de terem HM em comparação com indivíduos sem pré-hipertensão.^60^ Contudo, a estratégia de se selecionar pacientes com pré-hipertensão no consultório tem acurácia apenas modesta para prever a presença de HM.^60^

### 2.4.3. Prognóstico

Quando comparados aos normotensos, indivíduos com HM têm pior prognóstico, sendo este similar à HS. De maneira análoga, a HMNC apresenta pior prognóstico quando comparada à HC, tendendo a ser similar à HSNC.^53 , 61^

### 2.4.4. Tratamento

Em um primeiro momento, pode-se supor que o pior prognóstico associado à HM possa justificar o seu tratamento, tal como na HS.^62^ No entanto, até o momento, não há resultados de ensaios clínicos prospectivos analisando o efeito do tratamento da HM sobre o risco de eventos cardiovasculares e mortalidade.

Dada a presença de risco cardiovascular semelhante à HS, a orientação de mudança de estilo de vida pode ser apropriada.^9^Mesmo sem evidências conclusivas para tal, caso o tratamento medicamentoso seja instituído, sobretudo em pacientes com lesão de órgão-alvo estabelecida, faz-se necessário avaliar a resposta com base na medida da PA fora do consultório.^9 , 63^

**Figura 3:**
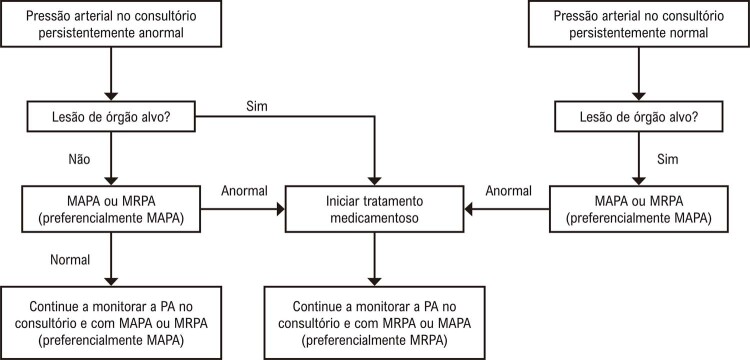
– Fluxograma para avaliação, diagnóstico e condutas na hipertensão mascarada e hipertensão do avental branco. Adaptado de Huang et al. ^63^

Um fluxograma adaptado de Huang et al. é sugerido nesta diretriz na Figura 3 , para avaliação, diagnóstico e condutas na HM e HAB, caso a MAPA ou MRPA estejam disponíveis.

## 2.5. Hipertensão Resistente

A hipertensão arterial resistente (HAR) é definida quando a PA permanece acima das metas recomendadas com o uso de três anti-hipertensivos de diferentes classes, incluindo um bloqueador do sistema renina-angiotensina (inibidor da enzima conversora da angiotensina ou bloqueador do receptor de angiotensina), um bloqueador dos canais de cálcio de ação prolongada, em doses máximas preconizadas e toleradas; e um diurético (preferencialmente, um tiazídico de longa ação).^64^ A HAR é uma indicação bem-definida para a realização da medida da PA fora do consultório.^5 , 18^ A MRPA e a MAPA são mandatórias no diagnóstico e no acompanhamento da HA resistente e refratária devido à grande magnitude do efeito do avental branco encontrada nessas condições clínicas, sendo fundamental para afastar a pseudorresistência e, portanto, definir os quatro comportamentos da HAR: HAR verdadeira, HAR do avental branco, HAR mascarada e HAR controlada.^5 , 18 , 64^ Embora a grande maioria dos estudos seja baseada na MAPA, a MRPA tem uma boa concordância com a MAPA nesses pacientes, sendo o método preferencial para o acompanhamento de hipertensos resistentes, principalmente nos hipertensos com valores mais elevados da PA em consultório.^65^

## 3. Indicações, Vantagens E Desvantagens da MAPA e MRPA

As indicações para uso da MAPA e MRPA estão apresentadas no Quadro 11 , enquanto as vantagens e desvantagens destes métodos estão apresentadas no Quadro 12 .

**Quadro 11:** – Indicações da MAPA e da MRPA (GR: I – NE: C)

Indicações	MAPA	MRPA
Suspeita de HAB – efeito avental branco	X	X
HA estágio 1 (140-159 e/ou 90-99 mmHg) no consultório	X	X
PA > 140/90 mmHg no consultório sem LOA e baixo RCV	X	X
Hipertensão sistólica isolada ou diastólica isolada no consultório	X	X
Suspeita de HM – efeito de mascaramento	X	X
PA faixa de pré-hipertensão (130-139 e/ou 85-89 mmHg)	X	X
PA < 140/90 mmHg no consultório com LOA e alto RCV	X	X
Suspeita de efeito do avental branco	X	X
PA elevada no consultório ou suspeita de pré-eclâmpsia em gestantes	X	X
Identificar HTNC, HAR e redução excessiva da PA	X	X
Ajustar a medicação anti-hipertensiva	X	X
Assegurar controle adequado da PA	X	X
Avaliar o controle da PA nas 24 horas, durante o sono e atividades diárias	X	
Identificar hipotensão postural, pós-prandial e na sesta	X	
Avaliar as variações da PA na disautonomia	X	
Avaliação de sintomas, principalmente de hipotensão	X	
Monitorar a eficácia do tratamento a longo prazo e melhorar a adesão		X

GR: grau de recomendação; NE: nível de evidência; PA: pressão arterial; MAPA: monitoração ambulatorial da pressão arterial; MRPA: monitoração residencial da pressão arterial; AMPA: automedida da pressão arterial; HA: hipertensão arterial; HAB: HA do avental branco; LOA: lesões de órgãos-alvo; HAR: HA resistente.

**Quadro 12:** – Limitações e vantagens da MAPA e da MRPA

Limitações	MAPA	MRPA
Braços com dificuldade do ajuste adequado do manguito	X	X
Situações clínicas associadas a distúrbios do movimento, como Parkinson	X	X
Arritmias cardíacas como fibrilação atrial, *flutter* atrial e extrassístoles ventriculares frequentes	X	X
Valores muito elevados da PAS	X	
Dificuldade do paciente e/ou do cuidador de executar as medidas		X
**Vantagens**	**MAPA**	**MRPA**
Medida da PA fora do consultório e sem a presença do médico	**X**	**X**
Correlação com prognóstico melhor que a PA no consultório	**X**	X
Pode reduzir gastos com saúde	**X**	**X**
É considerado padrão-ouro na avaliação da PA	**X**	
Avalia o controle da PA nas 24 horas; durante o sono e atividades diárias	**X**	
Avalia a elevação rápida da PA matinal	X	
Melhor método para monitoramento de longo prazo		X
Favorece o controle da PA e adesão ao tratamento		X
Custo menor que o da MAPA		**X**
**Desvantagens**	**MAPA**	**MRPA**
Disponibilidade por vezes limitada	**X**	**X**
Relutância de alguns pacientes em usar e repetir o exame	**X**	X
Pode ser desconfortável, principalmente durante o sono	X	
Frequentemente, a PA durante o sono não é calculada de acordo com os horários de sono do indivíduo	X	
Medida da PA somente em repouso na vigília		X
Requer orientação e treinamento; potencial para erro de medida: medida em momentos inadequados, número excessivo de medidas, indução de ansiedade, mudança da medicação pelo paciente, paciente relata os valores mais baixos		X

PA: pressão arterial; PAS: pessãoa rterial sistólica; MAPA: monitoração ambulatorial da pressão arterial; MRPA: monitoração residencial da pressão arterial.

**Figura 4:**
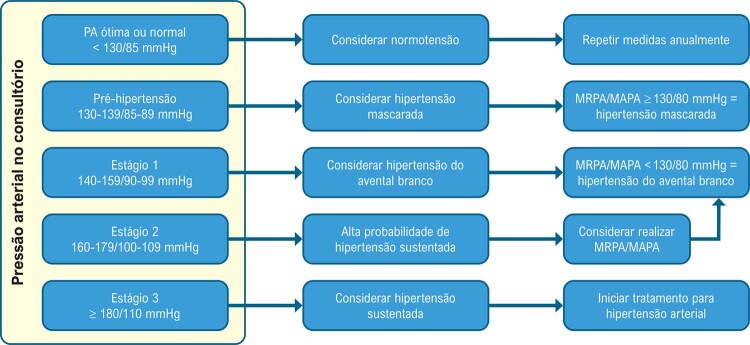
– Interpretação do comportamento da pressão arterial no consultório e fora dele (GR: IIa – NE: C). GR: grau de recomendação; NE: nível de evidência; PA: pressão arterial; MAPA: monitoração ambulatorial da pressão arterial; MRPA: monitoração residencial da pressão arterial.

Atualmente, o diagnóstico e o tratamento da HA têm como base as medidas de PA no consultório e fora dele. A Figura 4 apresenta um algoritmo que norteia como interpretar o comportamento da PA neste cenário.

## Parte 3 – Monitoração Ambulatorial da Pressão Arterial (MAPA)

## 1. Definição

Monitoração ambulatorial da pressão arterial de 24 horas (MAPA) é o método que permite o registro indireto e intermitente da PA durante 24 horas ou mais, enquanto o paciente realiza as suas atividades usuais na vigília e durante o sono.^18^

A MAPA foi descrita há mais de 50 anos e, atualmente, é realizada com o emprego de monitores totalmente automatizados.^66^

## 2. Protocolos para Realização da MAPA

Recomenda-se que o monitor seja programado para medir a PA entre 15 e 20 minutos durante a vigília e entre 20 e 30 minutos no período do sono.^5 , 9 , 18 , 67^ O período do sono pode ser programado previamente conforme o horário habitual de sono informado pelo paciente e definido posteriormente conforme o registrado no diário. Deve ser sempre colocado um manguito de tamanho adequado no braço não dominante, a não ser em condições especiais ou caso haja uma diferença da PA entre os braços acentuada (> 10 mmHg).

### 2.1. Reprodutibilidade da MAPA

A MAPA é um exame que apresenta boa reprodutibilidade desde que realizada em curto intervalo de tempo com as mesmas condições técnicas e clínicas. Os valores das médias de PA sistólica e PA diastólica obtida em 24 horas, vigília e sono, apresentam resultados semelhantes em exames consecutivos, realizados em curto intervalo de tempo.^68 , 69^

Em relação ao descenso do sono, também costuma haver boa reprodutibilidade, não havendo diferença significativa entre o primeiro e o segundo exame. A reprodutibilidade do comportamento da PA entre os períodos de vigília e sono é questionada na literatura por causa da probabilidade de 30 a 50% dos indivíduos mudarem de estado em exames subsequentes, especialmente quando existem variáveis que possam interferir, tais como estresse ao exame, mudanças farmacológicas e qualidade do sono.^18^

## 3. Orientações aos Pacientes e Profissionais de Saúde

### 3.1. Diário de Atividades Durante o Exame

**Figura 5:**
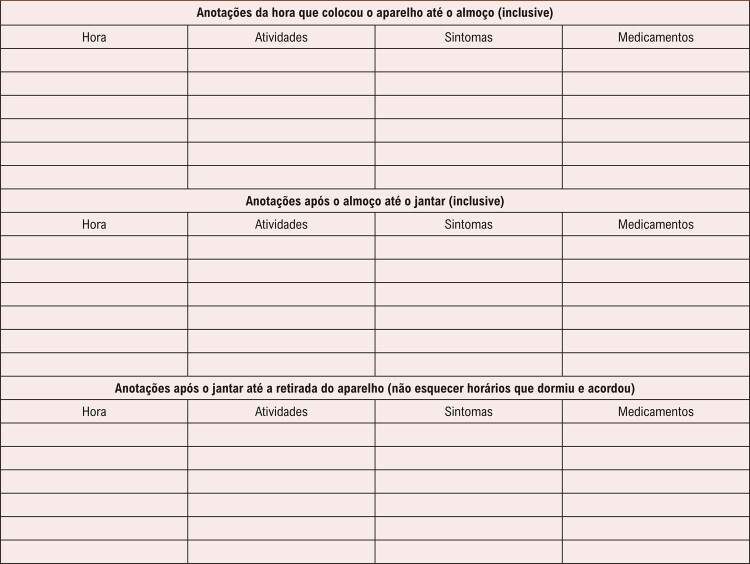
– Modelo de diário para registro das atividades durante o exame.

O paciente deve ser orientado a fazer anotações simplificadas no diário de atividades para que seja possível correlacionar as atividades, os sintomas e o uso de medicamentos durante o período de exame. Para assegurar o preenchimento correto, é recomendada a verificação dos registros feitos no momento da retirada do equipamento. Um modelo de Diário de Atividades é sugerido na Figura 5 , e as orientações para o seu preenchimento estão no Quadro 13 .

**Quadro 13:** – Orientações para o preenchimento do diário de atividades

Orientações gerais
***1.*** Todos os relatos anotados no diário devem ser sincronizados com o horário mostrado pelo monitor.
***2.*** Especificar horários e atividades realizadas nas 24 horas: profissionais, domésticas, escolares, físicas e de repouso.
***3.*** Orientações específicas
**Anotar: **
***a)*** Nome, dose e horário dos medicamentos utilizados durante o exame.
***b)*** Horário das refeições, incluindo o consumo de café.
***c)*** Na ocorrência do consumo de álcool, cigarro e outras drogas; informar horário e quantidade.
***d)*** Horários em trânsito e meios de locomoção.
***e)*** Ocorrência e horários de eventos estressantes.
***f)*** Presença de sintomas, preferencialmente, com horários de início e término, intensidade (forte, médio ou fraco), tipo (tontura, dor, falta de ar etc.) e medida tomada para sua resolução.
***g)*** Horários em que dormiu e acordou, inclusive durante o dia (sesta) e qualidade do sono, identificando-o como bom, regular e ruim, segundo sua percepção, comparando com a qualidade do sono do dia do exame com a que tem habitualmente.

### 3.2. Orientações Gerais

Para que o exame tenha a colaboração necessária do examinando, algumas orientações indispensáveis deverão ser dadas a ele no agendamento ( Quadro 14 ) e na instalação do equipamento ( Quadro 15 ). As atividades do profissional de saúde que realiza a instalação e retirada do aparelho estão nos Quadros 16 e 17, respectivamente.

**Quadro 14:** – Orientações para o paciente no momento do agendamento do exame de MAPA

No dia do agendamento do exame, os pacientes devem receber as orientações descritas a seguir:
***1.*** Realizar a MAPA, preferencialmente, em dia representativo das atividades habituais.
***2.*** Vestir camisa de manga larga ou sem manga para não limitar o movimento dos braços e interferir na instalação do manguito; as mulheres devem evitar o uso de vestido.
***3.*** Seguir a orientação do médico sobre o(s) medicamento(s) de uso crônico.
***4.*** Levar lista de medicamentos em uso com doses e horários da prescrição.
***5.*** Tomar banho antes do exame, pois não será permitido fazê-lo durante a realização do procedimento.
***6.*** Levar cinto para facilitar a colocação do monitor na cintura.

MAPA: monitoração ambulatorial da pressão arterial.

**Quadro 15:** – Orientações ao paciente no momento da instalação do aparelho de MAPA

Orientar o paciente quantos aos aspectos descritos a seguir:
***1.*** Não será permitido tomar banho durante o período do exame.
***2.*** Não fazer treinamento físico durante a realização do exame.
***3.*** Ensinar como acionar uma medida manual em caso de necessidade ou presença de sintomas.
***4.*** O braço deve ficar imóvel e relaxado ao longo do corpo durante as medidas.
***5.*** Eventual reajuste do manguito ao longo do dia e a colocação do monitor sob o travesseiro durante o período de sono.
***6.*** Como desligar o monitor caso haja alguma necessidade urgente ou mau funcionamento do equipamento.
***7.*** Não se deitar sobre o braço que está com o manguito.
***8.*** Preenchimento correto do diário, enfatizando sua importância.
**Recomendar:**
***1.*** Que o monitor não seja desconectado e o manguito não seja trocado de braço.
***2.*** Que o monitor não seja exposto a água, gelo, excesso de poeira ou calor.
***3.*** Que mantenha suas atividades habituais durante o exame.
***4.*** Que entre em contato em caso de urgência.

MAPA: monitoração ambulatorial da pressão arterial.

## 4. Interpretação dos Resultados

### 4.1. Critérios para Interpretação do Exame

Para um exame ser considerado válido para interpretação adequada, deverá apresentar um número mínimo de 16 medidas válidas na vigília e 8 durante o período de sono.^18^ Idealmente, o exame não deve ter períodos superiores a 2 horas sem medidas e duração menor que 22 horas. Dependendo do objetivo do exame e em situações especiais, a juízo clínico, pode ser aceito número menor de medidas que o preconizado ou ser analisado, isoladamente, o período com número de medidas adequados, seja a vigília ou o sono.

### 4.2. Valores de Anormalidade

Apesar da relação contínua entre a PA medida por MAPA e risco cardiovascular, na prática clínica, é necessário que se estabeleçam pontos de corte para anormalidade. Os pontos de corte de hipertensão arterial (HA) para PA medida pela MAPA atualmente são definidos por poucos estudos de coorte e por diretrizes internacionais (ver Tabela 2 do Capítulo 2).^54 , 56 , 70 , 71^

**Tabela 2:** – Classificação do comportamento da pressão arterial entre os períodos de vigília e sono (GR: I – NE: B)

Comportamento da pressão arterial (PA)	Descenso PA (%)
Descenso presente	≥ 10 ≤ 20
Descenso ausente ou ascensão da PA	≤ 0
Descenso atenuado	> 0 < 10
Descenso acentuado	>20

GR: grau de recomendação; NE: nível de evidência.

### 4.3. Comportamento da Pressão Arterial entre os Períodos de Vigília e Sono

A MAPA tem a característica única, entre os métodos de medida da PA, de registrar a PA no período de sono. Caracteriza-se o padrão vigília-sono avaliando-se a diferença percentual entre as médias de PA nesses dois períodos ( Tabela 2 ).^72^ Não existem evidências que esses padrões de descenso da PA tenham alguma implicação terapêutica, sendo, portanto, considerados tão somente marcadores de risco.

Os pacientes considerados de maior risco são aqueles em que a PA média no sono não cai entre 10 e 20% em relação à vigília.^73^

### 4.4. Ascensão Matinal da Pressão Arterial

A elevação matinal da PA reflete o aumento dessa variável nas primeiras horas da manhã e decorre da atividade simpática. Vários modos de se determinar a elevação matinal da PA têm sido propostos em diferentes estudos.^74 - 77^ Contudo, a dificuldade na padronização do cálculo desse parâmetro e a sua baixa reprodutibilidade indicam que ele não deve ser usado na prática clínica.^72^

### 4.5. Cargas de Pressão, Áreas sob as Curvas, Variabilidade da Pressão Arterial e Frequência Cardíaca

#### 4.5.1. Cargas de Pressão

**Figura 6:**
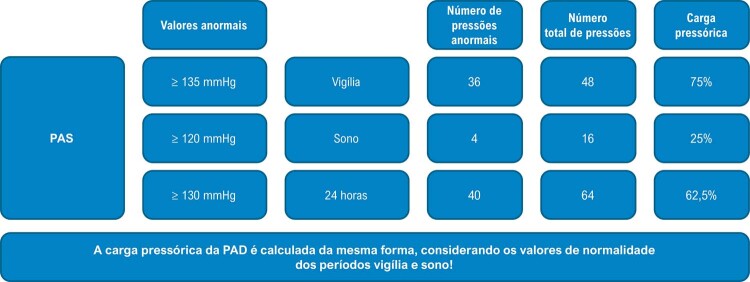
– Exemplo de como calcular a carga pressórica da PA. PAS: pressão arterial sistólica; PAD: pressão arterial diastólica.

Por definição, trata-se do percentual de medidas anormais para o período de registro obtido. Podem ser avaliadas nos períodos de vigília, sono e de 24 horas. A Figura 6 exemplifica o cálculo da carga pressórica. Por seu cálculo apontar apenas que houve valores elevados, mas não os quantificar, pode haver pacientes com uma mesma carga pressórica e valores médios de pressões completamente diferentes. Sua fraca correlação com desfechos clínicos torna seu uso dispensável na prática clínica.^78^

#### 4.5.2. Áreas sob as Curvas

É a área definida sob a curva de PA registrada que se localiza acima dos limites de normalidade da PA para o respectivo período analisado.^79^

Podemos considerar que esse parâmetro tem importância clínica maior que as cargas de pressões isoladas, pois leva em consideração, além da quantidade de medidas acima do normal no registro, o nível destss valores registrados.^79^ Com essa avaliação bidimensional, podemos obter uma estimativa mais precisa do impacto da PA de 24 horas. No entanto, esse cálculo de planimetria não está disponível nos programas de computador para laudos de MAPA utilizados comercialmente. A análise das correlações entre cargas de pressões, médias de 24 horas e subperíodos e áreas sob as curvas de pressões mostrou que há forte correlação entre os valores das áreas obtidas com os demais parâmetros. Igualmente, as áreas sob as curvas mostraram boa correlação com hipertrofia ventricular esquerda. Em contrapartida, por faltarem parâmetros de normalidade e evidências de correlação dessa medida com desfechos clínicos, não há ainda indicação para seu uso na prática clínica.

#### 4.5.3. Variabilidade da Pressão Arterial

Trata-se da variação dos valores de PA sistólica e diastólica obtidos no período de 24 horas do registro. Poderá ser avaliada individualmente no período de vigília, sono ou nas 24 horas. Nos relatórios, é expressa pelo desvio padrão dos valores pressóricos, embora também possa ser obtida pelo coeficiente de variância e “variabilidade real média”.^80^

A partir de estudos da medida contínua da PA, têm-se acumulado evidências que correlacionam o aumento da variabilidade a desfechos cardiovasculares, renais e neurológicos indesejáveis e a um maior risco de HAB e HM.^63^ Entretanto, por não ter valor de referência de normalidade e pela falta de dados que apoiem que o tratamento atuando sobre a variabilidade traria alguma vantagem clínica, a sua aplicabilidade fica prejudicada.

#### 4.5.4 Frequência Cardíaca

É a estimativa do valor da frequência dos batimentos cardíacos obtidos pelo pulso arterial durante o registro dos valores de PA nas 24 horas.^81^ É um dado pouco estudado em termos de significado clínico a partir do registro da MAPA, visto que os aparelhos convencionais estimam com pouca sensibilidade esse comportamento.

Por ser um indicador muito suscetível a erros e pela falta de parâmetros de normalidade, a frequência cardíaca obtida pela MAPA não tem aplicabilidade clínica.

## 5. Interpretação da MAPA e Emissão de Laudo

### 5.1. Emissão de Laudo

O laudo da MAPA deve conter os itens listados no Quadro 18 .^82^ Os diversos tipos de comportamento da PA, tais como normotensão, HA, HAB ou HM não devem ser estabelecidos, pois são diagnósticos clínicos.

**Quadro 18:** – Itens que deverão constar no relatório de MAPA (GR: I – NE: C)

Data e horário de início e término do exame
Número e porcentagem das medidas realizadas e das efetivamente válidas
Médias de PA sistólica nas 24 horas, vigília e sono
Médias da PA diastólica nas 24 horas, vigília e sono
Comportamento da PA entre vigília e sono
Episódios de hipotensão
Correlação entre atividades, sintomas e medicamentos
Conclusão

GR: grau de recomendação; NE: nível de evidência; PA: pressão arterial; MAPA: monitoração ambulatorial da pressão arterial.

### 5.2. Comportamento da Pressão Arterial Sistólica e Diastólica nas 24 Horas, Vigília e Sono

O laudo deve especificar as médias das pressões arteriais sistólicas e diastólicas durante o período de 24 horas, na vigília e no sono. Existe correlação positiva, tanto em hipertensos tratados quanto em não tratados, entre os valores PA sistólica e/ou PA diastólica de 24 horas, na vigília e/ou no sono com morbidade e mortalidade cardiovascular, além de lesões em órgãos-alvo.^71 , 83 - 85^

### 5.3. Comportamento da Pressão Arterial Sistólica e Diastólica entre os Períodos de Vigília e Sono

A classificação do comportamento da PA entre os períodos de vigília e sono está na Tabela 2 . O laudo deve descrever a classificação do descenso da PA sistólica e da PA diastólica, conjuntamente, quando as classificações forem iguais e, separadamente, quando forem diferentes.^86^

Para a definição dos períodos de vigília e sono, é fundamental a anotação dos horários em que o indivíduo submetido ao exame dormiu e acordou. Esses dados devem estar claramente anotados no diário de atividades. A qualidade do sono referida durante o exame também deve ser considerada no momento da interpretação.

Vale lembrar que a inversão do comportamento fisiológico da PA vigília-sono, a ausência de descenso ou a ascensão da PA podem estar relacionadas a determinadas condições, tais como distúrbio do sono provocado pelo exame, controle inadequado da PA em pacientes tratados, alguns tipos de HA secundária, indivíduos com apneia do sono, disautonomia e uso de alguns medicamentos como, por exemplo, a ciclosporina.

### 5.4. Pico de Pressão e Hipotensão

Os picos de PA são elevações de duas ou três medidas de forma progressiva, que estão muito acima das médias de PA observadas, antes e depois do evento. No entanto, deve-se observar que, na maioria das vezes, valores elevados e isolados da PA correspondem a artefatos e não devem ser caracterizados como picos de pressão. Como não há estudos prospectivos que avaliaram o prognóstico em decorrência de picos de PA, o seu significado é muito restrito e não se recomenda que sejam descritos no laudo.

**Figura 7:**
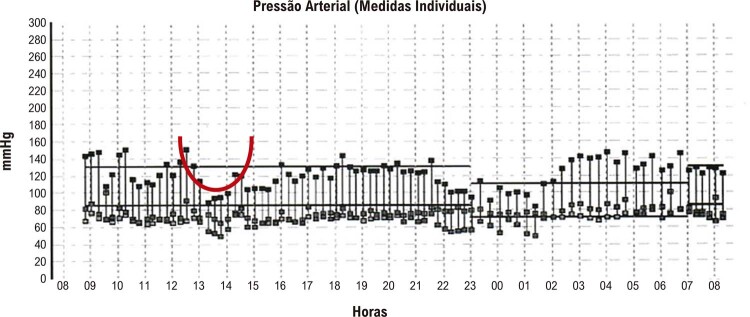
– Episódio de queda de pressão arterial na MAPA.

A hipotensão é caracterizada por episódios em que a PA apresenta valores bem mais baixos que as médias de PA observadas antes e depois do evento, desde que acompanhados de sintomas ( Figura 7 ). As reduções da PA não acompanhadas de sintomas não deverão ser descritas como hipotensão.

Episódios sintomáticos de diminuição da PA podem ocorrer nas seguintes situações: uso de medicamentos, síncope, lipotimia, hipotensão postural e neuropatia autonômica. Medidas isoladas e não acompanhadas de sintomas, ainda que com acentuadas quedas da PA, também podem ser decorrentes de artefato técnico.

### 5.5. Correlação entre Atividades, Medicamentos Utilizados e Sintomas

O correto preenchimento do “Diário de Atividades” pelo paciente é de extrema importância para a correlação das modificações da PA com os medicamentos utilizados, atividades e sintomas durante a realização do exame. Devem estar especificadas as doses dos medicamentos, horários das tomadas, e registro das principais atividades (dormir, acordar, desjejum, almoço e jantar).

Os sintomas deverão ser mencionados com a hora em que ocorreram e a intensidade. No laudo, deve ser mencionado se o sintoma descrito provocou alguma variação da PA, deverá ser registrado no diário se a medicação foi tomada no dia da realização do exame e deverão constar os anti-hipertensivos utilizados, bem como doses e horários das tomadas.

### 5.6. Conclusão

Na conclusão, devem constar as seguintes informações (GR: I – NE: C):

- Quando o paciente refere que a qualidade do sono foi boa ou regular:
  1. O comportamento normal ou anormal da PA sistólica e/ou PA diastólica durante as 24 horas.
  2. O descenso presente, ausente, acentuado ou atenuado da PA sistólica e/ou PA diastólica.
  3. O controle ou não da PA sistólica e/ou PA diastólica nos períodos de vigília e sono do exame quando o paciente refere uso de anti-hipertensivos.
- Quando o paciente refere que a qualidade do sono foi ruim não deve ser analisado o descenso da PA durante o sono, e o comportamento e o controle da PA devem ser descritos somente para o período de vigília:
  1. O comportamento normal ou anormal da PA sistólica e/ou PA diastólica durante a **vigília** .
  2. O controle ou não da PA sistólica e/ou PA diastólica no período de **vigília** do exame.

Exemplo de conclusão quando paciente refere que a qualidade do sono é boa ou regular:

1. Comportamento anormal da pressão arterial sistólica e normal da pressão arterial diastólica com base nas médias de pressão de 24 horas.
2. Descenso ausente da pressão arterial sistólica e acentuado da pressão arterial diastólica durante o sono.
3. A medicação referida controlou a pressão arterial diastólica, mas não controlou a pressão arterial sistólica durante os períodos de vigília e sono dessa monitoração.

Exemplo de conclusão quando paciente refere que a qualidade do sono é ruim:

1. Comportamento anormal das pressões arteriais sistólica e diastólica com base nas médias de pressão de vigília.
2. A medicação referida não controlou as pressões arteriais sistólica e diastólica durante o período de vigília dessa monitoração.

Exemplo de descrição da variação da pressão entre a vigília e o sono quando o paciente refere qualidade de sono ruim.

1. Variação da pressão entre a vigília e o sono.

Paciente refere qualidade de sono ruim. Assim, a análise do comportamento da pressão arterial durante o sono não pode ser realizada.

Recomenda-se que, ao final do laudo, seja colocada a seguinte ressalva:

A MAPA, como os demais exames complementares em medicina, deve ser avaliada segundo critérios do médico assistente.

Finalmente, é importante salientar que o modelo de laudo aqui sugerido é uma orientação do que pode ser feito. Cada médico pode estabelecer seu modelo de laudo desde que contenha as informações essenciais para o médico que solicitou o exame.

Outro aspecto importante é que o laudo deve se ater às informações oferecidas pelo exame, sem deduções e conclusões clínicas que não são fornecidas pelo exame.

## 6. Aplicações Clínicas da MAPA

### 6.1. Para Avaliação do Prognóstico de Pacientes com Hipertensão Arterial

Os diferentes componentes da PA obtidos com a MAPA fornecem subsídios relevantes para a avaliação do risco cardiovascular e do prognóstico em pacientes hipertensos.

As médias de PA de 24 horas, vigília e sono correlacionam-se mais fortemente com lesões de órgãos-alvo, morbidade e Mortalidade que as medidas casuais.^87^

O comportamento da PA durante o sono, parâmetro somente obtido por meio da MAPA dentre todos os demais métodos de medida indireta da PA, também determina risco cardiovascular, com associação independente entre o aumento da PA sistólica durante esse período e mortalidade por eventos cardiovasculares.^88^ Pacientes com descenso sistólico durante o sono atenuado, apresentaram maior incidência de infartos lacunares cerebrais que pacientes com descenso normal da PA.^89^

Atenuação do descenso da PA durante o sono associa-se a maior risco de eventos cardiovasculares também em indivíduos com doença renal crônica, em idosos e em pacientes com doença arterial coronariana.^89 - 91^

Associação independente do padrão de descenso da PA e eventos cardiovasculares foi igualmente demonstrada em coorte de hipertensos resistentes. Uma redução da PA inferior a 10%, ou sua elevação durante o sono, associou-se com desfecho combinado de eventos cardiovasculares e mortalidade total após seguimento médio de 4,8 anos.^92^

### 6.2. Para Avaliação da Eficácia Terapêutica Anti-hipertensiva

Diretrizes têm dado maior ênfase ao papel da MAPA no diagnóstico e no seu maior poder de estabelecer o prognóstico do que na avaliação da eficácia da terapêutica. Alguns estudos têm demonstrado resposta discrepante da eficácia do tratamento quando se comparam a MAPA e a PA de consultório.^93 , 94^Foi demonstrado controle em apenas 12% dos casos pela avaliação da PA de consultório, enquanto na MAPA houve controle em 33% dos casos. Além disso, 38% dos pacientes tiveram sua prescrição modificada pela MAPA, 32% tiveram que adicionar outra medicação e 14% dos pacientes novos diagnosticados no consultório foram mantidos sem medicação depois de realizarem a MAPA.^93^ Estudos longitudinais utilizando MAPA, desenhados especificamente para avaliação da eficácia terapêutica, são necessários antes de se generalizar as indicações do método a todos os hipertensos.

## 7. MAPA em Situações Especiais

### 7.1. Crianças e Adolescentes

Dados substanciais relacionam níveis elevados de PA medidos na infância e adolescência e danos atuais e futuros em órgãos-alvo.^95^

As definições ambulatoriais normativas para os valores da MAPA na população pediátrica são derivadas de estudos na população normal, e as recomendações para utilização de MAPA nessa população são baseadas em opiniões de especialistas, e não em evidências decorrentes de estudos bem delineados para esse fim.^96^

A MAPA é considerada procedimento obrigatório para confirmação do diagnóstico de HA em crianças e adolescentes, com medidas de consultório em nível de PA elevada por 1 ano ou mais, ou, em nível de HA estágio 1 em 3 visitas clínicas consecutivas, assim como para avaliação da HA e a ocorrência de padrões circadianos anormais de PA em crianças e adolescentes com condições de alto risco, tais como doença renal crônica, diabetes melito tipos 1 e 2, pré e pós-operatório de coarctação de aorta, transplante de órgãos sólidos, síndrome da apneia obstrutiva do sono, obesidade, suspeita de HM ou HAB e síndromes genéticas associadas à HA como síndrome de Williams, síndrome de Turner e neurofibromatose.^97^

A nova normativa para MAPA pediátrica recentemente publicada apresenta um novo esquema para classificação da medida de PA pela MAPA, que, além de favorecer a transição de cuidado do paciente adolescente para o adulto jovem, elimina o uso da carga pressórica ( Tabela 3 ).^98^

**Tabela 3:** – Classificação para PA de consultório pela MAPA em pacientes pediátricos (GR: IIa – NE: B)

	PAS/PAD consultório		PAS/PAD MAPA	
	< 13 anos	≥ 13 anos	< 13 anos	≥ 13 anos
PA normal	< p95	< 130/80	< Percentil 95 OU valores de corte para adolescentes*	< 125/75 mmHg 24 horas E < 130/80 mmHg vigília E < 110/65 mmHg sono
HAB	≥ p95	≥ 130/80		
HM	< p95	< 130/80	≥ Percentil 95 OU valores de corte para adolescentes*	≥ 125/75 mmHg 24 horas OU ≥ 130/80 mmHg vigília OU ≥ 110/65 mmHg sono
HA ambulatorial	≥ p95	≥130/80		

GR: grau de recomendação; NE: nível de evidência; HA: hipertensão arterial; PA: pressão arterial; PAS: pressão arterial sistólica; PAD: pressão arterial diastólica; HAB: hipertensão do avental branco; HM: hipertensão mascarada.

Em crianças < 13 anos, selecionar o valor mais baixo entre o percentil 95 para 24 horas e o respectivo ponto de corte adulto que consta da coluna ≥ 13 anos de idade.

### 7.2. Gestantes

O papel da MAPA na gestação ainda não está claramente definido. O seu uso é útil particularmente na primeira metade da gestação.^99^ A HAB, assim como a HM, pode ocorrer em até um terço das gestantes. Sua identificação é fundamental para evitar o tratamento desnecessário e potencialmente lesivo ao feto.^99^

A HAB tem prognóstico mais favorável que a hipertensão gestacional, persistindo em 50% dos casos ao longo da gestação e não se associando a complicações. Entretanto, 40% das gestantes desenvolvem hipertensão gestacional, e 8% das gestações cursam com pré-eclâmpsia.^100^ Por outro lado, 22% de 158 gestantes cuja HA foi confirmada pela MAPA desenvolveram pré-eclâmpsia.^100^ Nesse estudo, a HA durante o sono ocorreu em 60% dos casos, sendo mais associada com risco de pré-eclâmpsia e de complicações fetais. A suspeita diagnóstica de HM é mais difícil, devendo ser pesquisada na presença de lesão em órgão-alvo na gestante.

Esta diretriz sugere que:

- MAPA está indicada para avaliar a suspeita de HAB e de HM na gestação.
- Os valores de referência para o diagnóstico de hipertensão na gestação devem ser idênticos aos usados para a população em geral (MAPA ≥ 130/80 mmHg).^101^

### 7.3. Idosos

A MAPA pode dar informações clínicas valiosas em pacientes idosos, como nos casos de suspeita de hipotensão arterial postural, pós-prandial, medicamentosa e situacional, bem como na avaliação de disautonomias e síncopes. Nos idosos, algumas limitações devem ser salientadas: a) aceitam-se para os idosos os mesmos valores de normalidade da MAPA adotados para os adultos não idosos, e b) a redução do descenso da PA durante o sono, a pressão de pulso aumentada e a elevação matinal precoce da PA, comuns nos idosos, relacionam-se a aumento do risco cardiovascular.^84 , 102 , 103^

Pacientes com descenso acentuado, com queda > 20% da PA sistólica, tiveram maior incidência de acidente vascular cerebral isquêmico, enquanto o aumento da PA sistólica durante o sono associou-se ao maior risco de acidente vascular cerebral hemorrágico.^104^

### 7.4. Diabetes Melito

A ausência de descenso da PA durante o sono, a elevação da PA durante o sono em relação à de vigília, a HA durante o sono, a HM e a variabilidade pressórica são altamente prevalentes em pacientes com diabetes tipo 2, com ou sem história conhecida de HA.^105^ Em pacientes com diabetes melito, a MAPA pode contribuir para a avaliação de hipotensão secundária à neuropatia autonômica cardiovascular, muitas vezes relacionada a sintomas como síncopes, tonturas e sudorese, auxiliando no diagnóstico diferencial com hipoglicemia.^106^ A HA sistólica durante o sono destaca-se como um preditor de danos aos órgãos-alvo em pacientes com diabetes tipo 2.^105^ Por outro lado, a ausência de descenso da PA durante o sono foi associada à doença cardiovascular, neuropatia e retinopatia, enquanto a elevação matinal da PA foi associada à neuropatia.^107^

### 7.5. Doença Renal Crônica

Em qualquer estágio da doença renal, a MAPA pode identificar as alterações do padrão vigília/sono, detectar episódios de hipotensão arterial, HM e HAB.^51^ Além disso, nos indivíduos com DRC, os valores de PA obtidos com a MAPA estão correlacionados de forma independente com massa ventricular esquerda, ritmo de filtração glomerular, proteinúria e outras lesões em órgãos-alvo.^108 , 109^ Nos pacientes em hemodiálise, a MAPA de 24 horas pode não contemplar a avaliação da PA ao longo do ciclo dialítico. Assim, a realização de MAPA de 44 horas, instalada após a sessão de diálise do meio da semana (entre a segunda e terceira sessão da semana) e retirada imediatamente antes da sessão seguinte, permite avaliação mais adequada.^90^ Em termos práticos, se o *software* utilizado não contempla o protocolo de 44 horas, sugerimos a realização de dois exames de 22 horas em dias consecutivos. Essa diretriz não recomenda o uso da MAPA de 24 horas em pacientes em hemodiálise. Nessa população, o manguito não pode ser instalado no braço dos pacientes com fístula arteriovenosa. Pacientes submetidos à diálise peritoneal ambulatorial contínua ou automática também mostram alterações do padrão de comportamento de PA durante o sono.^110^

### 7.6 Síndrome da Apneia Obstrutiva do Sono

Na fisiopatologia da síndrome da apneia obstrutiva do sono (SAOS), os episódios de microdespertares e hipóxia intermitente deflagram, entre outros eventos, a ativação do sistema nervoso simpático, levando a consequente elevação da PA, principalmente no período de sono.^111^

Essas alterações fisiopatológicas determinam alterações significativas no comportamento da PA pela MAPA, aumentando de forma expressiva o percentual de pacientes com descenso atenuado ou ausente da PA durante o sono.^112^

Uma metanálise mostrou que a prevalência do descenso atenuado na PA pela MAPA em pacientes com SAOS foi de 59%, e a presença da SAOS pelo menos moderada, índice apneia-hipopneia > 15 eventos/hora, aumentou o risco de o paciente apresentar descenso atenuado da PA em pelo menos 1,67 vez. Tal observação pode explicar, pelo menos em parte, o maior risco cardiovascular apresentado por pacientes com SAOS.^113^

Estudo transversal incluindo 153 pacientes mostrou que pacientes com descenso ausente da PAS na MAPA tinham chance 3,5 vezes maior de apresentarem SAOS moderada quando realizavam a polissonografia, mostrando assim que dados advindos da MAPA podem triar pacientes com maior chance de terem o diagnóstico de SAOS quando da realização do exame de polissonografia.^114^

### 7.7. Insuficiência Cardíaca

A MAPA pode ser indicada para aperfeiçoar o tratamento de pacientes com insuficiência cardíaca (IC) cujos sintomas estejam relacionados a alterações da PA, como, por exemplo, em casos de dispneia paroxística noturna ou IC com fração de ejeção preservada. Igualmente, a MAPA pode ser útil para orientar a terapêutica de pacientes com sintomas causados por hipotensão, visto que muitos daqueles com IC avançada apresentam fadiga, sintomas de insuficiência coronariana ou manifestações encefálicas. A MAPA também pode ser utilizada na avaliação de pacientes com IC que serão submetidos a programas de exercício físico.^115^

Pacientes com IC apresentam mais frequentemente ausência de descenso noturno.^116^ Alterações do padrão vigília-sono têm sido associadas à gravidade da disfunção sistólica.^117^

Mais recentemente, a mortalidade total e cardiovascular foi avaliada em pacientes com IC com FE reduzida, IC com FE levemente reduzida e IC com FE preservada. A PA sistólica elevada estava associada a aumento de risco entre os pacientes com IC com FE preservada, mas não em pacientes com IC com FE reduzida ou IC com FE levemente reduzida.^118^ Nos pacientes com IC com FE reduzida, a PA sistólica/diastólica baixa e a presença de descenso durante o sono foram preditores de alta mortalidade quando comparados àqueles com PA sistólica/diastólica alta e sem descenso durante o sono.^119^

### 7.8. Exercício Físico

A prática de exercício físico durante a realização da MAPA pode produzir medidas incorretas ou perda de medidas.^120^ Após a execução de uma sessão de exercício físico, a PA diminui por várias horas, o que é mais evidente em hipertensos e pode modificar os valores médios da PA ambulatorial habitual do paciente.^121^ Assim, a prática de exercício físico deve ser evitada durante a realização da MAPA, e, também no dia que antecede o exame em pacientes que não praticam regularmente.

## 8. Custo-efetividade

A MAPA é uma estratégia de escolha para o diagnóstico e início de tratamento para a maioria dos adultos na atenção primária. Diagnosticando corretamente a HAB e a HM, o custo total do tratamento da HA e de futuros eventos cardiovasculares e cerebrovasculares é reduzido.^67^ O National Institute for Health and Care Excellence (NICE) realizou uma rigorosa análise de custo-efetividade que demonstrou que a MAPA seria não apenas o meio mais efetivo de fazer o diagnóstico de HA, mas também proporcionaria uma abordagem mais custo-efetiva que a medida de consultório ou a MRPA em todos os subgrupos de idade e sexo, conduzindo a uma melhora nos resultados da qualidade da assistência e na redução de gastos quando os custos de longo prazo foram levados em conta. O principal fator de economia de gastos nessa análise foi o fato de que as despesas com o tratamento da HA seriam evitadas devido à melhora de especificidade na realização de diagnóstico com a MAPA. O modelo sugeriu que a terapia anti-hipertensiva seria necessária em cerca de 25% menos pacientes do que se o diagnóstico fosse feito com base apenas na PA de consultório. A economia de custos com medicamentos superou os aumentos de custos associados à utilização da MAPA. As análises foram baseadas no modelo de prestação de cuidados de saúde do Reino Unido e podem não ser consideradas válidas para uso em outros países.^122^ O uso da MAPA diminui gastos com medicamentos e com consultas médicas, quando comparado com uso de medidas de consultório.^123^ Os benefícios da MAPA são inquestionáveis, principalmente na atenção primária a saúde e vêm sendo incorporados pelo Sistema Único de Saúde (SUS), tendo sido incorporada integralmente no sistema de saúde suplementar. Na América Latina, muitas Sociedades Científicas de HA e/ou Cardiologia têm dedicado especial atenção em suas diretrizes quanto ao uso da MAPA para o diagnóstico e controle da HA, de modo a estimular as autoridades de saúde a regulamentar o acesso e permitir que mais pacientes possam ter acesso aos benefícios desse exame.

## 9. Perspectivas

Desde o início da MAPA em 24 horas, em 1964, houve grande progresso com os equipamentos empregados para a realização do exame. Consequência dessa evolução tecnológica, houve maior conforto para os pacientes com o emprego desses monitores e menor custo para a realização do exame.

Em futuro próximo, esperam-se progressos igualmente nesse sentido e a produção de aparelhos capazes de registrar a PA perifericamente, batimento a batimento, por meio de *chips* que detectem fenômenos mecânicos decorrentes da dinâmica circulatória e transformá-los em valores de PA sistólica e PA diastólica.

Igualmente, já é realidade o registro em 24 horas de parâmetros da hemodinâmica central, incluindo PA aórtica, velocidade de onda de pulso e *augmentation index.*

Com o tempo, poderemos conhecer as mais amplas aplicabilidades desses parâmetros obtidos e, certamente, nesse momento, a execução desse tipo de exame deverá ser amplamente acessível na prática clínica diária.

As futuras aplicações e possibilidades de uso da MAPA envolvem:

- Monitores de MAPA com actígrafo acoplado.
- Manguitos ajustáveis.
- Avaliação de outros parâmetros, além de PA sistólica e PA diastólica, tais como frequência cardíaca, pressão de pulso, velocidade e forma de onda de pulso, variabilidade da PA nas 24 horas ou em subperíodos e elevação matinal da PA.
- Valores referenciais para MAPA de 24 horas resultantes de estudos com diversas populações em todo o mundo.
- Estudos prospectivos para avaliação de prognóstico e de eficácia do tratamento anti-hipertensivo em populações seguidas pela MAPA.
- Desenvolvimento de equipamentos de baixo custo para registro não invasivo da PA batimento a batimento.

## Parte 4 – Monitoração Residencial da Pressão Arterial

## 1. Introdução

A MRPA é um método que auxilia no diagnóstico de HA e acompanhamento de pacientes hipertensos por meio dos registros da PA por dias, fora do ambiente do consultório, no domicílio. Deve ser feita por indivíduo treinado na medida da PA pelo método oscilométrico, preferencialmente o paciente, ou outra pessoa desde que devidamente treinada, na impossibilidade de o paciente realizar a sua própria medida da PA. Sua característica fundamental é obedecer a protocolo previamente validado, estabelecido e normatizado.^1 , 5^

## 2. Orientações ao Paciente

A realização da MRPA com boa qualidade depende fundamentalmente das orientações fornecidas ao paciente. Essas devem abordar fatores que podem modificar a PA ou criar artefatos em sua medida, como os cuidados com o ambiente, o posicionamento e o preparo do paciente.^1 , 5^ Medidas domiciliares devem seguir a mesma sistemática da medida de PA no consultório e também estão sujeitas a variações transitórias ( Quadro 19 ).

**Quadro 19:** – Instruções gerais a serem fornecidas ao paciente para realizar MRPA

Condições
Ambiente silencioso com temperatura agradável
Há pelo menos 30 minutos sem fumar, utilizar cafeína, ter se alimentado ou praticado exercícios antes da medição da PA
Sentado e relaxado por pelo menos 3 minutos, idealmente 5 minutos, com bexiga vazia
Não falar durante e entre as medições
**Postura**
Sentado com as costas apoiadas na cadeira
Pernas descruzadas, pés apoiados no chão
Braço nu apoiado na mesa; palma da mão voltada para cima, meio do braço ao nível do coração
Enrole o manguito ao redor do braço de acordo com as instruções do equipamento
Utilize sempre o mesmo braço para realizar as medidas durante todo o exame. O braço escolhido será o de maior PA no consultório (idealmente, medida realizada de maneira simultânea)
Informar ao paciente sobre a variação da PA: “A pressão varia a cada batimento cardíaco”
Salientar que, na maioria das pessoas, a PA fora do consultório é mais baixa; em outras pessoas, a PA é mais alta em casa
Orientar para a realização das medidas nos dias e horários recomendados pelo protocolo, sem alterar a rotina de vida
Reforçar que é proibido medir a PA de outras pessoas durante a MRPA
Orientar o paciente a não modificar o esquema terapêutico em decorrência das medidas observadas ao longo do exame, que medidas eventualmente altas ou baixas não devem ser motivo de preocupação

PA: pressão arterial; MRPA: monitoração residencial da pressão arterial.

## 3. Protocolo para Realização do Exame

O protocolo de MRPA tem por objetivo representar a PA usual e ter, no seu resultado, aplicação clínica, auxiliando o médico na tomada de decisões.^124^ A reprodutibilidade da MRPA está diretamente relacionada ao número de medidas realizadas.^125^ Com base em diversos estudos e evidências disponíveis na literatura, são apresentadas, a seguir, as recomendações desta diretriz (GR: I – NE: C):^1 , 5^

- **Número de medidas:** devem ser obtidas idealmente de 24 a 36 medidas válidas.
- **Período de verificação:** o período sugerido é de 4 a 6 dias.
- **Dia 0 ou dia de instalação:** são realizadas medidas no consultório (idealmente três medidas, utilizando-se a média das duas últimas para o cálculo de reação de alarme/mascaramento) e medidas à noite em domicílio, para diminuir um possível efeito do avental branco. As medidas realizadas no consultório ou no domicílio no dia de instalação devem ser excluídas do cálculo da média da MRPA (utilizar para a média as medidas a partir do dia seguinte à instalação).
- **Dias 1, 2, 3, 4, 5 e 6 da MRPA:** no domicílio, são realizadas medidas de PA por mais 4, 5 ou 6 dias. O paciente deverá fazer 3 medidas pela manhã e 3 medidas ao entardecer ou à noite. Sempre após 5 minutos de repouso, antes das refeições, de bexiga vazia e antes do uso das medicações anti-hipertensivas (se for o caso). Se o paciente tiver se alimentado, aguardar 2 horas para realizar as medidas.
- **Exclusão de medidas:** devem ser excluídas medidas discrepantes, tais como PA diastólica > 140 mmHg ou < 40 mmHg, PA sistólica < 70 mmHg ou > 250 mmHg, PA sistólica < PA diastólica anterior ou seguinte, PA diastólica > PA sistólica anterior ou seguinte, pressão de pulso < 20 mmHg ou > 100 mmHg, desde que não exista justificativa clínica para preservá-las no conjunto de medidas obtidas.

**Figura 8:**
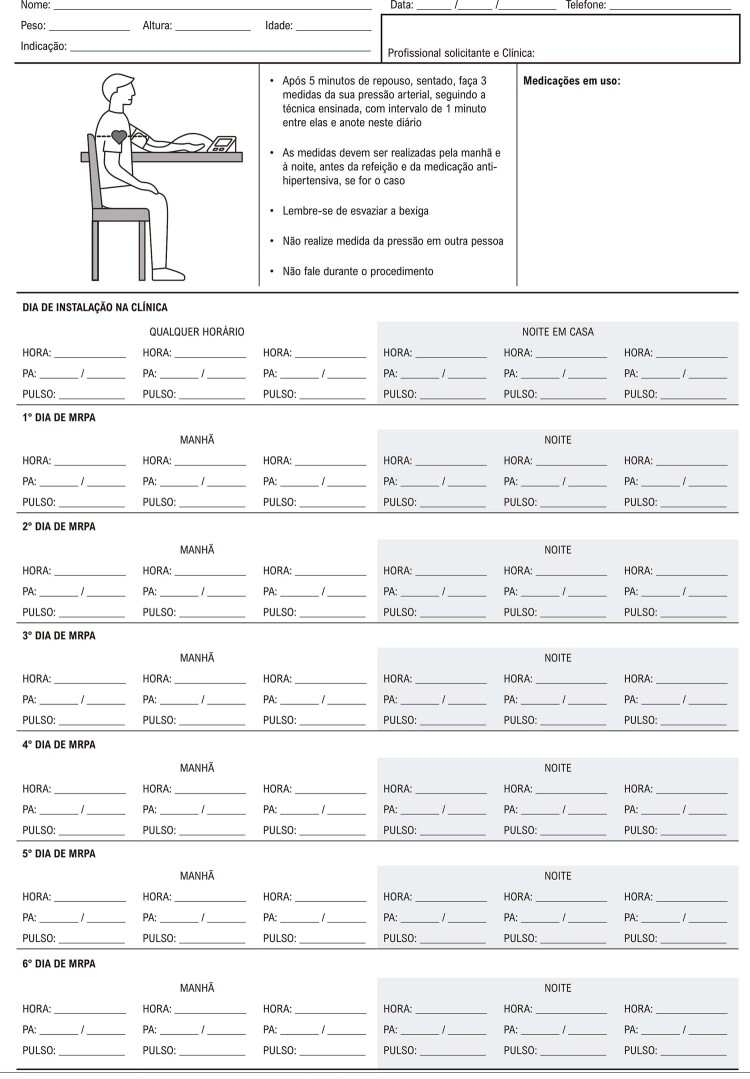
– Diário de monitoração residencial da pressão arterial (MRPA) para laudos digitados.

A elaboração de um diário do exame é de grande valor. Nele, devem estar contidas informações sobre o uso de medicamentos, e o paciente deve anotar as medidas realizadas. Isso é importante para que o protocolo do exame seja realizado adequadamente e facilite a transcrição dos valores de PA (no caso de equipamento sem transmissão dos dados) para confecção do laudo. A Figura 8 mostra sugestão de um diário de exames para laudos digitados.

## 4. Valores Referenciais de Anormalidade

Considera-se anormal a média das medidas da MRPA ≥ 130 mmHg para a PA sistólica e/ou ≥ 80 mmHg para a PA diastólica.^1 , 50 , 126 - 129^ Esses valores foram recomendados pela Diretriz Brasileira de HA 2020^1^ e diferem dos valores de normalidade anteriormente sugeridos pela 7ª Diretriz Brasileira de HA (publicada em 2016)^130^ e pela 4ª Diretriz de MRPA (publicada em 2018),^18^ as quais consideravam como anormal a média da MRPA ≥ 135 mmHg para a PA sistólica e/ou ≥ 85 mmHg para a PA diastólica. A Tabela 4 apresenta a estimativa da prevalência da HA na população brasileira baseada nesses dois diferentes alvos, enquanto a Tabela 5 estima a correspondência da PA da MRPA de acordo com a classificação da PA de consultório.

**Tabela 4:** – Prevalência de hipertensão e de seus fenótipos com base nos 2 limites da MRPA de acordo com 4ª Diretriz de MRPA e da Diretriz Brasileira de HA 2020 **1,18**

Hipertensão	MRPA ≥ 135/85 mmHg*	MRPA ≥ 130/80 mmHg**
Diagnóstico	37%	57%
Normotensão	47%	36%
HAB	16%	7%
HM	10%	22%
HS	27%	35%
Controle	40%	58%
Hipertensão controlada	45%	34%
HAB não controlada	15%	8%
HM não controlada	11%	22%
HS não controlada	29%	36%

MAPA: monitoração ambulatorial da pressão arterial; MRPA: monitoração residencial da pressão arterial; HAB: hipertensão do avental branco; HM: hipertensão mascarada; HS: hipertensão sistólica.

**Tabela 5:** – Correspondências estimadas da PA de acordo com medidas obtidas no consultório e na MRPA (GR: IIa – NE: B) **128**

Consultório PAS/PAD (mmHg)	MRPA PAS/PAD (mmHg)
< 120/< 80	< 120/< 75
120-129/80-84	120-124/< 75
130-139/85-89	125-129/75-79
140-159/90-99	130-139/80-89
160-179/100-109	140-149/90-95
≥ 180/≥ 110	≥ 150/≥ 95

GR: grau de recomendação; NE: nível de evidência; MRPA: monitoração residencial da pressão arterial; HA: hipertensão arterial; PA: pressão arterial; PAS: pressão arterial sistólica; PAD: pressão arterial diastólica. *A classificação é definida de acordo com a PA no consultório ou da MRPA e pelo nível mais elevado de PA, sistólica ou diastólica, de acordo com a técnica escolhida.

A média da PA a ser considerada para definição de normalidade é a do período total da MRPA; entretanto, a análise das médias nos períodos da manhã e da tarde/noite pode ser útil para a definição de estratégias mais individualizadas para o tratamento medicamentoso.

## 5. Emissão de Laudo e Interpretação dos Resultados

O laudo de MRPA deve conter os seguintes aspectos (GR: I – NE: C):

- Motivo da solicitação do exame: citar qual a indicação do exame.
- Descrição do protocolo utilizado: citar o número de dias de medidas efetivas, horário e número das medidas de cada dia:
- Qualidade do procedimento: o registro deverá ser aceito para interpretação quando atingir, pelo menos, 14 medidas no protocolo de 4 dias, 15 medidas ou mais no protocolo de 5 dias e, pelo menos, 18 medidas válidas no protocolo de 6 dias, com medidas em todos os dias, representativas dos períodos da manhã e da noite.
- Médias de PA: no relatório, devem ser citadas as médias total, diárias e as dos períodos da manhã e do final de tarde/noite, principalmente em pacientes sob terapêutica medicamentosa. As médias são obtidas a partir de registros efetivos de, no mínimo, 4 dias, e idealmente 6 dias de MRPA, sendo que o dia 0 não conta para cálculo da média, pois as medidas foram feitas no consultório e o aparelho foi entregue ao paciente.
- Os efeitos do avental branco e do mascaramento: informar os valores obtidos pelo cálculo da PA na clínica – MRPA.
- Valores de anormalidade: é recomendado considerar exame anormal quando as médias forem ≥ 130 mmHg e/ou ≥ 80 mmHg.
- Conclusão:

- Comportamento da PA durante a MRPA foi normal ou anormal (de acordo com a média global da MRPA).
- De acordo com a diferença da PA entre a clínica e a MRPA, foi observada reação de alarme e/ou de mascaramento (análise separadas da PA sistólica e PA diastólica).
- Informado (ou não informado) uso de medicação anti-hipertensiva.

Recomenda-se que, ao final do laudo, seja colocada a seguinte ressalva:

A MRPA, como os demais exames complementares em medicina, deve ser avaliada segundo critérios do médico assistente.

## 6. Aplicações da Monitoração Residencial da Pressão Arterial

### 6.1. Para o Estabelecimento do Comportamento da Pressão Arterial no Consultório e Fora dele

A PA oscila continuamente como resposta a estímulos internos e externos, podendo levar a diagnósticos errôneos de HA, resistência ao tratamento ou normotensão.^131^ A MRPA é superior à medida casual do consultório para avaliar o comportamento da PA, especialmente por permitir o diagnóstico de HAB e HM.^132 - 134^ Além disso, a MRPA pode avaliar variações dia a dia da e variações sazonais da PA, além de estabelecer separadamente o comportamento da PA no período matinal e no período do entardecer/anoitecer, muito embora o valor incremental dessas medidas em relação ao valor de PA médio obtido pela MRPA na predição de risco cardiovascular e renal ainda permaneça incerto.^135 - 137^

### 6.2. Para a Avaliação do Prognóstico

Diversos estudos demonstraram de forma consistente que a MRPA apresenta maior associação com lesões de órgão-alvo, particularmente com a hipertrofia ventricular esquerda, e maior capacidade de predizer eventos cardiovasculares e renais do que a PA obtida no consultório.^138 - 140^ Por exemplo, resultados de uma metanálise incluindo aproximadamente 18.000 indivíduos mostraram que a PA na MRPA foi superior à PA obtida no consultório na predição de mortalidade cardiovascular (risco relativo [intervalo de confiança de 95%] = 1,29 [1,02-1,64] para cada aumento de 10 mmHg na PA sistólica na MRPA; e risco relativo [intervalo de confiança de 95%] = 1,15 (0,91-1,46) para cada aumento de 10 mmHg na PA sistólica obtida no consultório).^138^

Evidências também mostraram que MRPA se associa a lesões de órgãos-alvo com confiabilidade semelhante a MAPA.^139^ Além disSo, uma metanálise de estudos que realizaram exclusivamente MRPA ou MAPA mostrou que a HM e a HMNC diagnosticadas por ambos os métodos apresentavam valor similar na predição de eventos cardiovasculares e mortalidade.^141^ Em conjunto, esses dados sugerem que a MAPA e a MRPA possam ter valor equivalente na predição de risco cardiovascular. Contudo, há poucas evidências disponíveis comparando diretamente a *performance* da MRPA com a MAPA na predição de eventos cardiovasculares em uma mesma população, o que limita conclusões mais robustas sobre esse tema.^142^ Uma análise recente do estudo PAMELA mostrou que a MRPA e a MAPA foram superiores à PA obtida no consultório na predição de risco e ainda sugeriu que a MRPA poderia ter uma acurácia melhor que a MAPA na predição de mortalidade cardiovascular e total.^143^ Além disso, há ainda demonstrações de que a variabilidade dia a dia da PA detectada pela MRPA tenha valor preditivo de risco de doenças cerebrovasculares, renais e cardiovasculares.^140^

### 6.3. Para Avaliação da Terapêutica Anti-hipertensiva

A principal contribuição da MRPA para o tratamento da HA é a caracterização dos fenótipos da HA, em especial a hipertensão do avental branco não controlada e a hipertensão mascarada não controlada, mas também a confirmação da HA controlada, HA não controlada e HA resistente.^1 , 5 , 18 , 144^ A identificação desses fenótipos permite uma abordagem mais personalizada de cada caso, com ajustes terapêuticos individualizados.^5 , 18 , 144^

A MRPA é de amplo acesso, baixo custo e de fácil aceitação, tornando a sua realização e repetição bem aceitas pelo paciente e pelo médico. Assim, é o método de medida da PA preferencial para o acompanhamento de pacientes hipertensos em tratamento, permitindo a titulação de fármacos para a obtenção do controle da PA e seu monitoramento a longo prazo.^5 , 144 - 146^

Em relação às metas pressóricas a serem alcançadas na MRPA com o tratamento, considera-se recomendável atingir valores de PA sistólica < 130 mmHg e de PA diastólica < 80 mmHg.^145 , 147^ Estudos demonstraram que o uso da MRPA pode aumentar o engajamento do paciente e a sua adesão e persistência ao tratamento a longo prazo, e pode ser usada para telemonitoramento da PA. Todos esses aspectos podem contribuir para o melhor controle da PA, especialmente se combinados com medidas educativas e de aconselhamento.^148 - 150^

### 6.4. Em Situações e Populações Especiais

#### 6.4.1. Crianças e Adolescentes

Crianças e adolescentes também podem apresentar HA do avental branco e HA mascarada. Por este motivo, a realização de medidas de PA fora do consultório também tem sido encorajada. Deve-se tomar o cuidado de utilizar equipamentos validados para essa população e manguitos adequados. Existem poucos estudos que avaliaram valores de normalidade de MRPA em crianças e adolescentes. Em adolescentes, essa diretriz sugere que os valores maiores ou iguais aos percentis 95 de tabelas de normalidade obtidas de uma população brasileira possam ser utilizados para diagnosticar HA na MRPA.^151 , 152^

#### 6.4.2. Gestantes

Recomenda-se que a PA seja medida nas mulheres grávidas em cada visita pré-natal. Sabemos, porém, que mesmo com visitas pré-natais regulares, isso pode não ser suficiente para identificar a pré-eclâmpsia ou a HAB, que é de ocorrência comum no final da gestação.^144 , 153^ Dentre as vantagens do uso da MRPA no período gestacional, estão a boa aceitação pelas mulheres, além de melhorar a supervisão do tratamento, reduzindo o número de visitas médicas. A posição sentada parece ser apropriada para a MRPA durante a gravidez.^144 , 153 , 154^ Além disso, é importante que sejam utilizados equipamentos com validação específica para essa população.

Durante a gravidez, recomendamos o uso da MAPA e da MRPA para avaliação de HAB e HM, a fim de evitar o tratamento desnecessário e potencialmente lesivo ao feto.^155^ Recomenda-se o uso da MAPA na avaliação da PA antes das 20 semanas e MRPA após a vigésima semana.^153^

#### 6.4.3. Idosos

A MRPA é um recurso de extrema importância na avaliação inicial e no controle terapêutico periódico de pacientes idosos, colaborando para um melhor prognóstico.^156 , 157^ Essa população habitualmente possui alto risco CV, maior variabilidade da PA e menor tolerabilidade ao tratamento inadequado, como, por exemplo, o uso de anti-hipertensivos em indivíduos com HAB.^158^

Existem divergências sobre se a idade é um fator de risco para maior prevalência de HAB e HM.^50 , 159^ O método é factível com mínimo treinamento em gerontes, devendo-se ter cuidado maior para o treinamento inicial em alguns perfis: aqueles com mais de 80 anos, pouca escolaridade, indivíduos com declínio cognitivo ou restrições físicas que demandem ajuda de outras pessoas.^160^ Uma alternativa, para melhor controle da PA, seria o uso do telemonitoramento em hipertensos idosos.^161^

#### 6.4.4 Diabetes Melito

O diabetes melito (DM) é uma doença com diversos agrupamentos fisiopatogênicos, mas que, em geral, aumenta em cerca de duas vezes o risco de desfechos cardiovasculares e renais como acidente vascular cerebral, DAC, DRC e morte cardiovascular.^162 , 163^ Os pacientes com DM, particularmente aqueles do agrupamento fisiopatogênico centrado na obesidade visceral e resistência à insulina, têm mais frequentemente HM que a população sem a doença.^43^ Em um pequeno estudo observacional com 170 pacientes com DM tipo 2, observou-se melhor grau de identificação de lesões microvasculares quando utilizamos a MRPA no controle da PA, comparativamente à PA de consultório.^164^ Esse achado sugere o grande potencial dessa medida para o manejo clínico e a necessidade de ensaios clínicos que definam a relevância clínica nessa população.

#### 6.4.5. Doença Renal Crônica

Fenótipos como HA do avental branco e HA mascarada são muito frequentes em pacientes com doença renal crônica (DRC).^165 , 166^ A utilidade da MRPA é indiscutível nos pacientes com DRC em tratamento conservador, diálise peritoneal (DP) e hemodiálise (HD), inclusive em transplantados renais, por predizer a progressão da doença renal e o risco de eventos cardiovasculares e morte.^167^ No entanto, sua principal limitação decorre das alterações do padrão noturno da PA, que são comuns, e que não podem ser acessados pela MRPA. Possivelmente, o telemonitoramento domiciliar noturno possa ser uma alternativa, o que está por ser determinado na DRC e no transplante renal.^168^

Nos pacientes em tratamento conservador e nos transplantados renais, a MRPA segue as recomendações habituais.^169^ Em pacientes dialíticos, recomendamos o seguinte protocolo:

- **Número de medidas:** devem ser obtidas idealmente 36 medidas.
- **Período de verificação:** o período sugerido é de 6 dias.
- **Dia 0 ou dia de instalação:** são realizadas medidas no consultório ou clínica de hemodiálise, nunca no braço da fístula arteriovenosa, idealmente três medidas (utilizando-se a média das duas últimas para o cálculo de reação de alarme/mascaramento) e medidas à noite em domicílio, para diminuir um possível efeito do avental branco. As medidas realizadas no consultório ou no domicílio no dia de instalação devem ser excluídas do cálculo da média da MRPA.
- **Dias da MRPA:** No domicílio, são realizadas medidas de PA por mais 6 dias. O paciente deverá fazer 3 medidas pela manhã e 3 medidas ao entardecer ou à noite. Sempre após 5 minutos de repouso, antes das refeições, de bexiga vazia e antes do uso das medicações anti-hipertensivas (se for o caso). Se o paciente tiver se alimentado, aguardar 2 horas para realizar as medidas. Nos pacientes que realizam hemodiálise clássica (duas a três vezes semanais), as medidas realizadas nos dias de diálise devem ser excluídas do cálculo da média da MRPA. Nos casos de hemodiálise diária e diálise peritoneal, todas as medidas serão consideradas para o cálculo da média.

#### 6.4.6. Obesidade

A avaliação da PA nos indivíduos obesos impõe desafios na prática clínica. Nessa população, a variabilidade pressórica e a prevalência de HAB e HM é maior do que nos não obesos. Dessa forma, a MRPA é uma ferramenta fundamental para definição do fenótipo clínico desses pacientes.^170 , 171^ Contudo, a correta medida da PA é, muitas vezes, limitada pela grande circunferência e/ou forma cônica do braço desses indivíduos, além da baixa disponibilidade de manguitos apropriados. Neste contexto, o uso de manguitos inadequados pode superestimar a pressão arterial.^172^ Na ausência de manguitos com comprimento e/ou forma compatíveis para a medição da PA no indivíduo obeso, o uso de aparelhos de punho validados e calibrados pode ser uma alternativa para realização da MRPA nesses indivíduos.^144^

#### 6.4.7. Arritmias

Os aparelhos de MRPA, especialmente os comercializados atualmente, são validados para medida da PA em pacientes com arritmias cardíacas, especialmente fibrilação atrial.^173^ Alguns aparelhos automáticos oscilométricos são equipados com algoritmo específico que podem sugerir a presença de fibrilação atrial, podendo auxiliar no diagnóstico dessas condições, principalmente na população idosa.^168 , 174 , 175^

## 7. Custo-efetividade

Os custos em saúde são uma preocupação mundial, e as exigências para sua contenção são adotadas universalmente. Análise de custo-efetividade permite avaliar custo (valor monetário) com resultados (efetividade [p. ex., vidas salvas]) aplicando diferentes métodos de intervenção.^176 , 177^

Recente análise de custo-efetividade concluiu que MRPA é mais eficaz que a avaliação convencional da PA em consultório e requer menor investimento financeiro e humano do que a MAPA.^123 , 178^

## 8. Perspectivas

Nos últimos anos, inúmeros equipamentos sem manguito têm sido disponibilizados para uso, com a alegação de que medem com precisão a PA. Em geral, esses aparelhos dispõem de um sensor que avalia a pulsação das artérias e estima a PA, baseando-se especialmente na análise da onda de pulso.^179^

Acredita-se que esse novo método tenha grande potencial, por permitir a obtenção de inúmeras medidas ou mesmo medidas contínuas da PA durante dias ou semanas sem o desconforto da compressão induzida pela insuflação. Contudo, a precisão e a utilidade dos dispositivos sem insuflação são incertas e devem ser validadas em grandes estudos randomizados e controlados em diferentes condições clínicas, comparando-se com o padrão-ouro de medida (MAPA ou medida invasiva). Por conseguinte, não devem ser utilizados para decisões de diagnóstico ou tratamento aqui no Brasil, por enquanto.

A MRPA com medidas realizadas durante o sono já é uma realidade em alguns países do mundo. Trata-se de um método que permite avaliar a PA durante o sono e analisar o descenso noturno por dias ou semanas.^180^ No Brasil, entretanto, ainda não dispomos de equipamentos validados pela ANVISA que realizam essa medida.

## Parte 5 – Pressão Central, Velocidade da Onda de Pulso E *Augmentation Index*

## 1. Introdução

Os valores de PA diferem significativamente entre as regiões central e periférica da árvore arterial; a PA sistólica (PAS) é mais elevada nas artérias periféricas do que nas centrais, enquanto a PA diastólica (PAD) e a média (PAM) diferem apenas ligeiramente. Idade e genética influenciam muito a diferença entre as curvas de pressão periférica e central, e essa diferença pode chegar a 20 mmHg para a PAS em indivíduos jovens. Esse efeito é conhecido como amplificação da PAS ou pressão de pulso amplificada. Em idosos, a pressão de pulso amplificada é menor devido ao aumento da rigidez arterial (RA) e ao retorno precoce das ondas refletidas, de modo que a PAS central pode ser próxima à periférica. Assim, o intervalo de tempo relativo às ondas de pressão que são transmitidas para a frente e para trás na aorta é considerado um parâmetro importante para definir a PAC^181 , 182^

## 2. Definições

O aumento da PA está diretamente relacionado ao aumento do risco cardiovascular (CV) por disfunção e dano endotelial e por aumento da RA resultante da agressão na camada média vascular.^183 - 187^ A RA é definida como um conjunto de propriedades dos vasos que determinam suas características biofísicas. Dentre elas, a distensibilidade, a elasticidade e a complacência interferem na dinâmica do fluxo sanguíneo a cada ciclo cardíaco.^183^Alterações na microvasculatura, arteriosclerose, disfunção endotelial e aumento da RA configuram danos resultantes da hipertensão arterial (HA) e de outras doenças como o *diabetes melito* (DM), obesidade e dislipidemia, recebendo também influência do componente genético e da idade.^184 , 185 , 188 - 192^ O aumento da RA é a principal causa do aumento da PAS que se observa no processo de envelhecimento.^193^

### 2.1. Velocidade de Onda de Pulso

Definida como a distância percorrida entre dois pontos do sistema arterial pela onda de pressão gerada após ejeção ventricular na unidade de tempo, a VOP é medida em metros por segundo (m/s).^184 , 194 , 195^ A VOP é o método padrão-ouro para quantificar a RA, e quanto mais intensa for a RA, maior será a VOP.^196^ A determinação da VOP pode ser realizada de forma simples, em ambiente ambulatorial e com método não invasivo.^183 , 197^ O método de referência atual na pesquisa clínica é a VOP carótida-femoral (VOPcf).

### 2.2. Augmentation Index

O AIx é definido como a razão entre a pressão determinada pela onda refletida e a onda de ejeção. Essa medida tem relação direta com a VOP, e inversa com a FC,^192 , 198^ e tem a vantagem de levar em consideração o tempo das ondas para a frente e para trás que são os principais determinantes da PAC.^199 , 200^ O AIx descreve a relação entre a PAC e a onda de pulso refletida, incorporando a magnitude e a velocidade das ondas refletidas. Consequentemente, esse índice pode ser definido como medida de intensidade de reflexão das ondas de pulso.^201 , 202^

A FC deve ser levada em consideração para a correção da AIx, assim o AIx@75 é o AIx corrigido para FC de 75 batimentos por minuto (bpm). Esse índice é informado pelos equipamentos de medida de RA, em resultado da aplicação da fórmula: AIx@75 = Aix – 0,39 × (75 – FC).^203^

### 2.3. Pressão Central

A PAC é a pressão exercida pela coluna sanguínea, em cada momento, nas artérias aorta e carótida, sendo uma aproximação à PA exercida no coração e no cérebro. Por esse motivo, costuma ser um marcador mais relacionado à morbimortalidade CV do que a PA periférica.^204 , 205^ Essa medida já pode ser realizada de forma não invasiva por diversas metodologias e aparelhos validados.^206^

## 3. Indicações

As possíveis indicações de PAC, VOP e AIx estão apresentadas de forma sumária na Tabela 6 .

**Tabela 6:** – Possíveis indicações da medida da pressão central, augmentation index e velocidade da onda de pulso (GR: IIa – NE: C)

Parâmetro	Possíveis indicações
Pressão sistólica central (PSC)	Avaliar hipertensão espúria na hipertensão sistólica isolada do jovem
Velocidade da onda de pulso (VOP)	Reestratificação dos pacientes pré-hipertensos e hipertensos de risco baixo ou intermediário Pacientes avaliados pelo escore SAGE ≥ 8
*Augmentation index* (AIx)	Reservado apenas para ambiente de pesquisa clínica

GR: grau de recomendação; NE: nível de evidência.

## 4. Vantagens da Medida da Pressão Central, *Augmentation Index* e Velocidade da Onda de Pulso

- Identificação de lesão subclínica em órgão-alvo: VOP ≥ 10 m/s.^207 , 208^

**Avaliação prognóstica:** aumento de 1 m/s na VOP se associa com aumento de 14% na ocorrência de eventos CV e de 15% na mortalidade.^205^

- AIx é um preditor de desfechos e de lesões de órgãos-alvo (LOA).^209 , 210^
- Essas medidas podem fornecer maior precisão para o diagnóstico da HA, maior segurança na decisão terapêutica e melhor definição do prognóstico.^211^

## 5. Limitações da Medida da Pressão Central, *Augmentation Index* e Velocidade da Onda de Pulso

- Pouca disponibilidade de serviços.
- Alto custo dos equipamentos.
- Medidas obtidas pelo método oscilométrico ainda necessitam de mais estudos epidemiológicos, especialmente quanto ao valor prognóstico.

## 6. Técnicas Disponíveis para Verificação dos Parâmetros Centrais e de Rigidez Arterial

A tonometria de aplanação, assim como os mecanorreceptores piezelétricos, sensíveis à pressão intravascular, pode ser utilizada como substituta da PAC, devido à proximidade anatômica com a aorta ascendente. A tonometria de aplanação, na radial, obtém a medida da PAC a partir de uma função matemática de transferência generalizada, tendo com uma das limitações desse método a variação dependente do operador. A técnica oscilométrica braquial tem como vantagem a praticidade para uso no consultório, além de não sofrer com variação operador dependente. No entanto, sofre grande crítica pela forma como esses dispositivos são calibrados, visto que tendem a subestimar a verdadeira pressão intra-arterial braquial e, consequentemente, a PAC.^1 , 212 - 219^

### 6.1. Métodos para Medida Indireta da Pressão Arterial Central

- **Tonometria arterial direta** : é o método utilizado no Complior Analyse®; neste caso, a forma da onda de pressão é obtida diretamente por aplanação na artéria carótida comum e apresenta correspondência com a forma de onda de pressão da aorta ascendente.^212 , 213^
- **Tonometria arterial indireta** : é o método utilizado no SphygmoCor® a partir da medida da PA na artéria braquial e da aplanação da arterial radial; neste caso, a forma da onda de pressão é reconstituída indiretamente a partir de um algoritmo.^212 , 213^
- **Método oscilométrico braquial** : é o método utilizado no Mobil-O-Graph®, Dyna-MAPA AOP® e Arteris® a partir da medida da PA na artéria braquial, utilizando-se algoritmo e função de transferência; neste caso, a forma da onda de pressão é reconstituída indiretamente.

Apesar de a tonometria arterial indireta ser considerada padrão-ouro na avaliação da rigidez arterial pela obtenção da VOPcf, a mensuração pelo método oscilométrico permite uma execução de forma mais simplificada, reprodutível e com resultados confiáveis.^214 , 215 , 220 , 221^

## 7. Protocolos para Medidas de PAC, VOP E AIx

### 7.1. Protocolo para Realização das Medidas de Parâmetros Centrais pelo Método Tonométrico

Pelo método direto, os sensores piezoelétricos são colocados nas artérias carótida e femoral e capazes de registrar as curvas de variação do diâmetro arterial secundárias a alterações na pressão intra-arterial com gravação simultânea do sinal central e periférico. A pressão arterial central é obtida diretamente da onda da pressão carotídea. Também é possível avaliar a VOP em três segmentos arteriais diferentes em uma única aquisição, para o estudo de artérias periféricas.^183 , 219^

Pelo método indireto, o sistema é composto por um tonômetro de aplanação e um eletrocardiograma, a VOP é medida em dois pontos e alinhada com a onda R do complexo QRS como ponto de referência. A forma de onda da pressão central e os valores ascendentes da pressão da aorta são definidos por meio de uma função de transferência.^183 , 219^

### 7.2. Protocolo para Realização do Triplo Tiro (Medida do Consultório) pelo Método Oscilométrico

Dependendo do equipamento utilizado, as medidas centrais da PA (PAC, VOP E AIx) obtidas pelo método oscilométrico podem ser realizadas em intervalos de tempo preestabelecidos. Deve-se utilizar a metodologia de preparo para medida da PA de consultório, incluindo a escolha adequada do manguito e do braço, conforme preconizado pelas Diretrizes Brasileiras de HA de 2020.^1^

Após a escolha do braço, aplicar o manguito conectado ao equipamento programado para realizar 15 minutos de monitoração, com disparos que avaliaram a pressão braquial e parâmetros centrais a cada 3 minutos, sendo consideradas as últimas três medidas ou as três medidas válidas (protocolo conhecido como Triplo PWA). Alguns equipamentos já possuem a função Triplo PWA estabelecida, não sendo necessária nenhuma programação prévia.

### 7.3. Protocolo para Realização das Medidas de Parâmetros Centrais da PA de 24 Horas pelo Método Oscilométrico

O mesmo protocolo de preparo, orientações ao paciente, escolha do braço/manguito propostos para realização daMAPA de 24 horas deve ser seguido para realização da monitoração de 24 horas de medidas de parâmetros centrais da PA. Sugerimos, para maior conforto durante a realização do exame, que o equipamento seja programado para realizar medidas com intervalos de 30 minutos (vigília e sono). Devem ser consideradas pelo menos 16 e 8 leituras diurnas e noturnas válidas, respectivamente. Além disso, o paciente deve ser alertado sobre a dupla insuflação durante as medidas, evitando assim que o paciente desconfie de erro na aquisição das medidas.

## 8. Valores de Referência 216 , 218 , 222 - 225

### 8.1. Valores de Referência para Velocidade de Onda de Pulso (VOP)

Como a idade e a PA têm uma grande influência na RA, os valores de referência para VOP costumam ser apresentados em categorias derivadas dessas variáveis. Nas Tabelas 7 e 8, são sugeridos valores de referências para VOP, cujas medidas foram obtidas principalmente por equipamentos tonométricos ou piezoelétricos.^226^

### 8.2. Valores de Referência para a Pressão Sistólica Central (PSc)

Os valores de referência para PSC utilizando os equipamentos Sphygmocor, Omron HEM-9000AI, PulsePen e tonometria carotídea direta e de acordo com categorias de idade, gênero e fatores de risco e de PA periférica estão mostrados nas Tabelas 9 e 10.^227^

### 8.3. Valores de Referência para VOP, PAC e AIx Utilizando o Método Oscilométrico na População Brasileira

Um estudo multicêntrico brasileiro, incluindo 6.499 indivíduos de 4 centros, descreveu os valores de referência para VOP, PSc e AIx na população brasileira, cujas medidas foram obtidas por meio do método oscilométrico.^212^ Nas Tabelas 11 e 12, são mostrados os valores de referência dessas medidas conforme a faixa etária, o gênero e os fatores de risco CV.^214^

Diante do exposto, podemos concluir que, nos dias de hoje, já existem valores de referência para os diversos dispositivos de avaliação da RA e PAC, inclusive para a população brasileira. Contudo, pouco ainda se sabe sobre os valores de anormalidade para as medidas de parâmetros centrais da PA capazes de predizer eventos cardiovasculares, especialmente quando obtidas pelo método oscilométrico. Os valores de anormalidade sugeridos para as medidas de parâmetros centrais da PA estão apresentados na Tabela 13 .

**Tabela 13:** – Valores de anormalidade para PAC, VOP e AIx (GR: IIa – NE: C). **216,218,222-225**

Parâmetros	Medida no consultório	MAPA 24 horas	MAPA vigília	MAPA sono
PAS central	≥ 130 mmHg	≥ 120 mmHg	≥ 125 mmHg	≥ 110 mmHg
PAD central	≥ 90 mmHg	≥ 80 mmHg	≥ 85 mmHg	≥ 70 mmHg
VOP	≥ 10 m/s	≥ 10 m/s	≥ 10 m/s	≥ 10 m/s
AIx	Não disponível			

GR: grau de recomendação; NE: nível de evidência; PAC: pressão arterial central; PAS: pressão arterial sistólica; PAD: pressão arterial diastólica; MAPA: monitoração ambulatorial da pressão arterial; MRPA: monitoração residencial da pressão arterial; VOP: velocidade de onda de pulso; AIx: Augmentation Index

## 9. Valor Prognóstico das Medidas Derivadas dos Parâmetros Centrais

Evidências indicam que a PAC está mais associada aos riscos CV tradicionais e às lesões de órgãos-alvo. A RA é um dos principais determinantes da PAC e tem sido considerada de alto valor preditivo para eventos CV. Da mesma maneira, como descrito a seguir, estudos prospectivos observacionais têm demonstrado o valor preditivo de parâmetros hemodinâmicos centrais para eventos CV na população geral, nos idosos e nos pacientes com doença coronária e com doença renal crônica (DRC).^228^

No Strong Heart Study, a PPc foi superior à periférica como preditor de eventos CV fatais e não fatais.^229^ Outros estudos encontraram igual superioridade em idosos normotensos ou hipertensos e em indivíduos de origem asiática.^227 , 228^

No estudo MESA (Multiethnic Study of Atherosclerosis), parâmetros de reflexão da onda de pulso foram associados com novos eventos CV e incidência de insuficiência cardíaca (IC) em indivíduos sem evidências DCV prévia.^229 , 230^

Em metanálise envolvendo mais de 5 mil indivíduos normotensos e hipertensos, alguns com doença coronária ou DRC, para cada aumento de 10 mmHg na PPc e PSc, havia um aumento de 14% no risco CV e 9% no risco de DCV, respectivamente.^231^ Médias da PAC ambulatorial de 24 horas também já foram consideradas melhores preditores de risco CV em relação às medidas de PA periférica em normotensos e hipertensos.^232^

Em pacientes com DRC em fases mais iniciais, a PPc foi capaz de predizer a progressão para DR terminal e, nos estágios 2 a 4, foi demonstrada relação independente entre PSC e mortalidade geral.^233^ O efeito adverso do aumento da reflexão da onda de pulso arterial sobre o sistema CV de pacientes com DRC terminal também já foi demonstrado.^234^

O valor clínico da medida da PAC foi inicialmente demonstrado no estudo ASCOT-CAFE (Anglo-Scandinavian Cardiac Outcomes Trial – Conduit Artery Function Evaluation). O estudo principal ASCOT já havia relatado um resultado CV mais favorável nos pacientes hipertensos tratados com um regime terapêutico com um inibidor da enzima conversora de angiotensina (IECA) associado a um bloqueador de canal de cálcio (BCC) em relação a outro esquema baseado em betabloqueador (BB) e diurético (DIU).^235 , 236^

No subestudo CAFE, embora sem diferença nos níveis de PA periférica entre os dois grupos, o tratamento com IECA + BCC promoveu maior redução da PAC.^45^

O estudo REASON foi capaz de demonstrar que um efeito favorável da associação IECA + DIU sobre o coeficiente de reflexão permaneceu presente mesmo após 9 meses de tratamento, embora sem efeito adicional sobre a PA periférica.^237^

A predição de eventos cardiovasculares (ECV) e mortalidade por todas as causas através da análise da hemodinâmica central não invasiva permitiu calcular o valor preditivo das pressões centrais e índices hemodinâmicos centrais para ECV e mortalidade por todas as causas através das medidas da VOP, PSc, PPc, AIx, podendo-se afirmar: um aumento da VOP aórtica em 1 m/s correspondeu a um aumento de risco de 14%, 15% e 15% no total de ECV, mortalidade CV e mortalidade por todas as causas.^205^ Aumento de 10 mmHg na PSc determina RR para ECV de 8,8%. Aumento de 10 mmHg na PPc determina RR para ECV de 12,9%. Aumento de 10% no AIx determina RR para ECV totais de 29,4% e mortalidade por todas as causas da ordem de 38,4%.^205^

A VOP tem valor preditivo na morbidade e mortalidade CV e é atualmente considerada o método padrão-ouro na avaliação da RA e do envelhecimento arterial com boa correlação com risco de morte CV, ECV e mortalidade por outras causas.^197 , 205^

**Figura 9:**
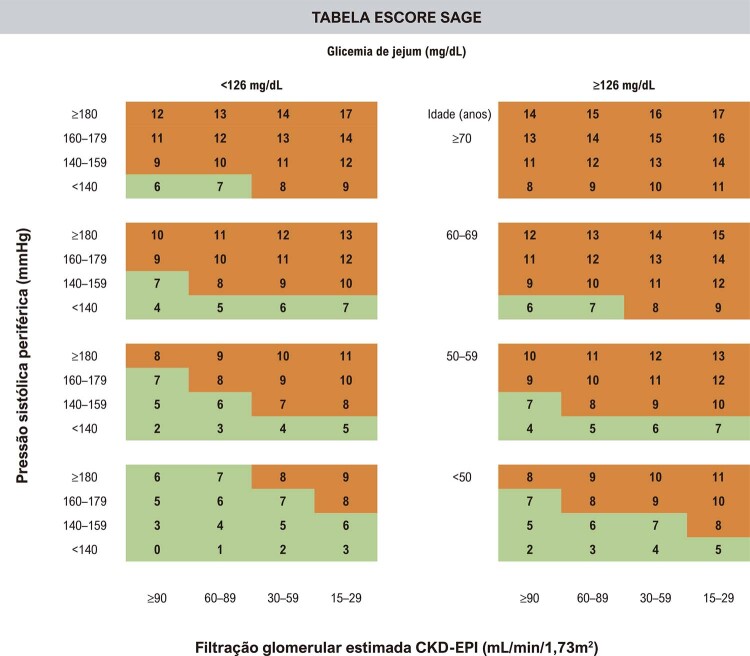
– Tabela de pontuação escore SAGE. 238 , 239 Em laranja (SAGE ≥ 8): alta probabilidade de RA elevada (VOP ≥10 m/s) e, em verde, (SAGE <8): baixa probabilidade de RA elevada. VOP: velocidade de onda de pulso; RA: rigidez arterial.

Com o objetivo de permitir uma identificação prática dos indivíduos com maior risco de apresentar a VOP aumentada, foi desenvolvido e validado um escore clínico que avalia variáveis facilmente disponíveis e foi denominado SAGE: (S) *systolic blood pressure* (PAS), (A) *age* (idade), (G) *fasting plasma glucose* (glicemia de jejum) e (E) *estimated glomerular filtration rate* (filtração glomerular estimada pela CKD-EPI), resumidos na Figura 9 .^242^ Esse escore também foi aplicado em hipertensos brasileiros avaliados pelo método oscilométrico e quando ≥ 8 foi eficaz na identificação de um alto risco de VOP ≥ 10 m/s, apresentando sensibilidade de 67,19% (IC 95%: 60,1-73,8) e especificidade de 93,95% (IC 95%: 91,8-95,7).^239^

## 10. Parâmetros Centrais de 24 Horas

A avaliação da PAC e dos indicadores de RA ao longo das 24 horas ainda é pouco utilizada na prática clínica, mesmo com evidências crescentes em todo o mundo de sua validade preditiva.^217^ Há necessidade de ampliar os conhecimentos científicos acerca do acompanhamento durante as 24 horas. Entretanto, indícios apontam que monitorar esses parâmetros nas condições da vida diária pode favorecer a avaliação e o prognóstico clínico de doenças CV, a possibilidade de categorização dos fenótipos da HA, especialmente a hipertensão do avental branco (HAB) e a hipertensão mascarada (HM), bem como a investigação específica da PA na vigília e no sono.^1 , 45 , 214 , 217^

Um consórcio de pesquisa internacional (20 centros, 14 países e 5 continentes) de parâmetros centrais de 24 horas obtidos através do Mobil-O-Graph^®^ analisou uma parte do seu banco de dados (2.092 adultos) e evidenciou que a PSc de 24 horas estava mais associada a danos cardíacos hipertensivos (massa e hipertrofia do ventrículo esquerdo), do que a pressão braquial de 24 horas. Esse mesmo grupo, em publicação recente, usando 130.804 medições de PSc válidas de 2.423 adultos não tratados, propôs pragmaticamente como limite superior normal para PSc de 24 horas 120 mmHg (calibração C1 = sistólica/diastólica).^222 , 240^

Metanálise recente revisou as atuais tecnologias não invasivas de 24 horas para obtenção da PSc, VOP, AIx e as evidências que apoiam seu uso no tratamento clínico de pacientes com HA ou em risco de complicações CV, e concluiu que os estudos realizados até o presente momento sugerem que os parâmetros centrais de 24 horas podem representar uma ferramenta promissora para avaliar a função vascular, estrutura e danos nas condições da vida diária e promover o rastreio precoce em indivíduos com maior risco.^217^ Ainda existe uma escassez de estudos avaliando o valor preditivo de VOP de 24 horas. Além disso, a precisão das medidas pode variar entre os diferentes dispositivos, afetando a generalização de resultados dos estudos. São ainda necessários estudos longitudinais que validem o valor preditivo dos parâmetros centrais de 24 horas.

## 11. Perpectivas

A avaliação da RA no panorama das recomendações para estratificação de risco CV como maneira de identificar a presença de LOA ainda subclínicos já foi incorporada pelas principais diretrizes.^1 , 216 , 217^ Nesse contexto, a análise da RA pela VOP e os índices de hemodinâmica central (PAS, PPC e AIx) são úteis para acompanhamento clínico de pacientes com HA e risco CV baixo ou intermediário; hipertensos jovens; pré-hipertensos; diabéticos; DRC; história familiar de DCV precoce e outros fatores de risco.^205 , 207 , 241 - 245^ Embora a utilização da VOP com a finalidade de identificar LOA esteja estabelecida na literatura, assim como seu ponto de corte > 10m/s, valores que definem a normalidade para a VOP, corrigidos para sexo e idade, ainda podem ser melhor explorados.^1 , 216 , 218 , 223 - 225^

Por meio das interações da genética com a epigenética, e a capacidade do sistema biológico em lidar com o resultado dessas combinações, pode haver uma diferença entre a idade cronológica e biológica. Esta é a base para os novos conceitos em envelhecimento vascular. O envelhecimento vascular acelerado ocorre em indivíduos em que a idade biológica vascular é superior à idade cronológica, e estes apresentarão manifestações mais precoces de doenças. O termo SUPERNOVA é reservado aos indivíduos que apresentam um envelhecimento vascular em que a lesão/rigidez arterial é significativamente inferior ao esperado para um indivíduo saudável da mesma idade. Entretanto, mesmo que essa habilidade de envelhecer lentamente seja predeterminada pelos genes, o estilo de vida e as intervenções farmacêuticas podem desacelerar a senescência vascular e melhorar o prognóstico.^206 , 219 , 246^

Os estudos que direcionam o tratamento medicamentoso utilizando a PSc, até o momento, demonstraram apenas redução do número de fármacos anti-hipertensivos, sem resposta na função do ventrículo esquerdo.^247 - 249^ Em outro estudo que avaliou a utilização de combinação livre ou combinação fixa de fármacos visando a metas de PA sistólica central e braquial, a classe dos IECA em combinação fixa permitiu uma maior eficiência em atingir metas pressóricas. Partindo da repercussão do estudo CAFE, tornou-se evidente que os fármacos apresentam efeitos distintos sobre os valores pressóricos (central e periférico), que resultam em desfechos clínicos distintos.^250 , 251^

Todos esses aspectos nos permitem concluir pelo enorme potencial de uma verdadeira evolução para a medicina de precisão em seu conceito mais puro, identificando precocemente o dano ou até prevenindo que o mesmo aconteça, tratando cada indivíduo com a estratégia mais adequada para as suas características clínicas e, dessa forma, reduzindo ainda mais os desfechos CV.

Em conclusão, esta diretriz não recomenda o uso rotineiro da avaliação da PSc, VOP e AIx. Para isSo, estudos com desfechos clínicos robustos necessitam ser realizados para definição dos valores de normalidade e prognóstico dessas medidas.

**Figura 2:**
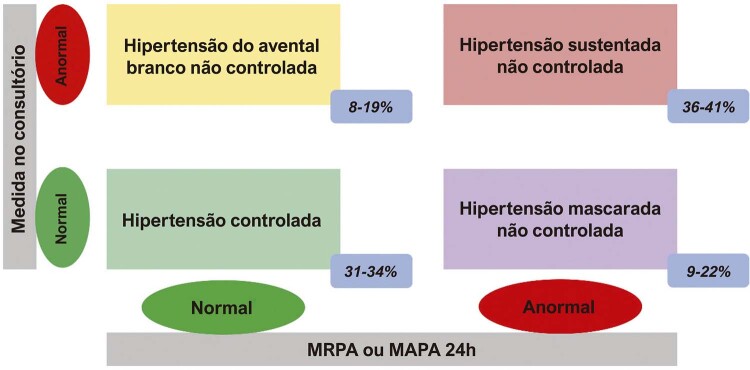
– Comportamento da PA e suas prevalências em hipertensos tratados. 43 , 45 MAPA: monitoração ambulatorial da pressão arterial; MRPA: monitoração residencial da pressão arterial; PA: pressão arterial.

**Tabela 7:** – Valores de referência da VOP categorizados por idade na população normal **226**

Idade (anos)	Média (+2 SD)	Mediana (10-90 pc)
30	6,2 (4,7-7,6)	6,1 (5,3-7,1)
30-39	6,5 (3,8-9,2)	6,4 (5,2-8,0)
40-49	7,2 (4,6-9,8)	6,9 (5,9-8,6)
50-59	8,3 (4,5-12,1)	8,1 (6,3-10,0)
60-69	10,3 (5,5-15,0)	9,7 (7,9-13,1)
≥70	10,9 (5,5-16,3)	10,6 (8,0-14,6)

VOP: velocidade de onda de pulso; SD: desvio padrão; 10 pc: limite superior do 10º percentil; 90 pc limite inferior do 90º percentil.

**Tabela 8:** – Valores de referência da VOP categorizada por idade e pressão arterial **226**

Idade (anos)	PA ótima	PA normal	PA normal alta	Estágio 1 HA	Estágio 2/3 HA
**VOP média (+2 SD)**					
< 30	6,1 (4,6-7,5)	6,6 (4,9-8,2)	6,8 (5,1-8,5)	7,4 (4,6-10,1)	7,7 (4,4-11,0)
30-39	6,6 (4,4-8,9)	6,8 (4,2-9,4)	7,1 (4,5-9,7)	7,3 (4,0-10,7)	8,2 (3,3-13,0)
40-49	7,0 (4,5-9,6)	7,5 (5,1-10,0)	7,9 (5,2-10,7)	8,6 (5,1-12,0)	9,8 (3,8-15,7)
50-59	7,6 (4,8-10,5)	8,4 (5,1-11,7)	8,8 (4,8-12,8)	9,6 (4,9-14,3)	10,5 (4,1-16,8)
60-69	9,1 (5,2-12,9)	9,7 (5,7-13,6)	10,3 (5,5-15,1)	11,1 (6,1-16,2)	12,2 (5,7-18,6)
≥ 70	10,4 (5,2-15,6)	11,7 (6,0-17,5)	11,8 (5,7-17,9)	12,9 (6,9-18,9)	14,0 (7,4-20,6)
**VOP mediana (10-90 pc)**					
< 30	6,0 (5,2-7,0)	6,4 (5,7-7,5)	6,7 (5,8-7,9)	7,2 (5,7-9,3)	7,6 (5,9-9,9)
30-39	6,5 (5,4-7,9)	6,7 (5,3-8,2)	7,0 (5,5-8,8)	7,2 (5,5-9,3)	7,6 (5,8-11,2)
40-49	6,8 (5,8-8,5)	7,4 (6,2-9,0)	7,7 (6,5-9,5)	8,1 (6,8-10,8)	9,2 (7,1-13,2)
50-59	7,5 (6,2-9,2)	8,1 (6,7-10,4)	8,4 (7,0-11,3)	9,2 (7,2-12,5)	9,7 (7,4-14,9)
60-69	8,7 (7,0-11,4)	9,3 (7,6-12,2)	9,8 (7,9-13,2)	10,7 (8,4-14,1)	12,0 (8,5-16,5)
≥ 70	10,1 (7,6-13,8)	11,1 (8,6-15,5)	11,2 (8,6-15,8)	12,7 (9,3-16,7)	13,5 (10,3-18,2)

PA: pressão arterial; VOP: velocidade de onda de pulso; SD: desvio padrão; 10 pc: limite superior do 10º percentil; 90 pc: limite inferior do 90º percentil.

**Tabela 9:** – Valores de referência da PAC categorizada por idade, sexo e fatores de risco **227**

Idade (anos)	População normal		População com fatores de risco cardiovascular	
	Feminino	Masculino	Feminino	Masculino
< 20 (n = 1.104)	97 (86, 91, 102, 109) n = 350	105 (95, 99, 109, 113) n = 290	99 (88, 93, 105, 120) n = 182	109 (96, 102, 117, 127) n = 282
20-29 (n = 4.157)	95 (80, 88, 102, 110) n = 1.411	103 (92, 97, 109, 115) n = 880	101 (88, 91, 110, 124) n = 888	110 (95, 102, 120, 130) n = 974
30-39 (n = 6.386	98 (84, 90, 108, 119) n = 1.860	103 (88, 95, 112, 120) n = 1.259	111 (92, 100, 127, 141) n = 1.373	114 (95, 103, 129, 144) n = 1.889
40-49 (n = 9.595)	102 (87, 93, 113, 123) n = 2.318	106 (90, 97, 114, 123) n = 2.068	116 (95, 104, 133, 146) n = 2.196	118 (97, 106, 132, 144) n = 2.995
50-59 (n = 11.950)	110 (93, 100, 119, 127) n = 2.002	110 (96, 102, 118, 126) n = 1.997	120 (100, 109, 134, 148) n = 4.251	123 (102, 111, 137, 150) n = 3.646
60-69 (n = 7.779)	114 (97, 105, 122, 129) n = 1.057	114 (97, 105, 122, 128) n = 1.410	128 (105, 115, 141, 154) n = 2.656	128 (105, 115, 142, 155) n = 2.629
> 70 (n = 4.445)	118 (100, 109, 126, 131) n = 530	116 (99, 107, 124, 130) n = 747	138 (113, 126, 152, 164) n = 1.567	135 (113, 124, 147, 160) n = 1.592

PAC: pressão arterial central. Valores descritos nos percentis 50 (10, 25, 75 e 90).

**Tabela 10:** – Valores de referência da PA de acordo com a classificação da pressão arterial e fatores de risco cardiovascular **227**

Categoria da pressão arterial	População normal		População com fatores de risco cardiovascular	
	Feminino	Masculino	Feminino	Masculino
Ótima (n = 17.678) 108 (96, 102, 114, 117)	97 (84, 90, 104, 110) n = 6.415	100 (88, 94, 106, 111) n = 4.035	102 (89, 95, 108, 112) n = 4.082	101 (90, 96, 107, 112) n = 3.146
Normal (n = 9.313) 123 (120, 121, 126, 128)	116 (104, 110, 121, 125) n = 1.902	112 (102, 106, 117, 122) n = 2.669	116 (107, 111, 120, 123) n = 2.281	113 (103, 108, 118, 122) n = 2.461
Normal alta (n = 7.148) 133 (128, 130, 136, 138)	126 (115, 120, 131, 135) n = 1.212	122 (110, 115, 128, 132) n = 1.947	125 (116, 120, 130, 133) n = 1.861	123 (111, 116, 128,132) n = 2.128
Estágio 1 (n = 3.288) 143 (130, 137, 150, 155)			137 (122, 129, 144, 150) n = 1.276	133 (119, 126, 142, 148) n = 2.012
Estágio 2 (n = 1.930) 161 (146, 154, 168, 174)			154 (128, 142, 161, 168) n = 798	148 (128, 138, 158, 165) n = 1.132
Estágio 3 (n = 701) 183 (162, 178, 193, 206)			173 (153, 164, 183, 194) n = 312	171 (143, 158, 183, 192) n = 389
ISH (n = 5.255) 147 (141, 143, 155, 163)			140 (128, 134, 148, 156) n = 2.507	137 (122, 129, 144, 152) n = 2.748

PA: pressão arterial. Valores descritos nos percentis 50 (10, 25, 75 e 90). As categorias de pressão arterial são referentes à pressão arterial braquial.

**Tabela 11:** – Valores de referência para PAC de acordo com sexo e idade na população normal e com fatores de risco cardiovascular **214**

Idade (anos)	População normal		População com fatores de risco cardiovascular	
	Feminino	Masculino	Feminino	Masculino
**Variável: Pressão sistólica central**				
< 30	101 (90, 93, 113, 119)	113 (104, 109, 120, 123)	118 (102, 109, 127, 131)	123 (107, 114, 132, 144)
30-39	109 (96, 102, 117, 123)	114 (102,110,121, 127)	120 (102, 110, 130, 143)	125 (108, 116, 133, 141)
40-49	110 (99, 103, 117, 122)	116 (102,109, 122, 126)	121 (104, 112, 134, 146)	123 (108, 115, 131, 141)
50-59	110 (97, 104, 120, 124)	112 (100, 106, 118, 124)	124 (106, 114, 135, 146)	124 (105, 114, 134, 144)
60-69	114 (100, 105, 120, 125)	112 (96, 101, 120, 127)	127 (105, 115, 141, 154)	123 (103, 112, 136, 149)
≥ 70	113 (100, 103, 121, 126)	116 (94, 104, 125, 129)	131 (108, 118, 146, 165)	125 (102, 111, 140, 156)
**Variável: Pressão diastólica central**				
< 30	73 (60, 66, 77, 85)	76 (66, 71, 82, 87)	82 (68, 73, 90, 97)	83 (72, 77, 93, 100)
30-39	77 (67, 71, 83, 88)	80 (71, 75, 85, 88)	86 (71, 77, 95, 105)	88 (75, 80, 96, 103)
40-49	79 (67, 73, 84, 88)	81 (74, 77, 86, 89)	86 (71, 78, 94, 103)	90 (75, 82, 97, 104)
50-59	76 (64, 70, 82, 85)	82 (70, 77, 86, 88)	84 (71, 77, 92, 100)	88 (75, 80, 97, 103)
60-69	76 (66, 71, 81, 87)	80 (68, 72, 83, 87)	81 (67, 74, 90, 98)	85 (71, 77, 93, 101)
≥ 70	76 (60, 70, 79, 83)	79 (60, 70, 84, 90)	81 (66, 72, 89, 97)	82 (68, 74, 91, 98)
**Variável: Pressão de pulso central**				
< 30	29 (23, 27, 37, 43)	36 (26, 32, 43, 53)	34 (24, 28, 41, 48)	38 (26, 31, 46, 52)
30-39	30 (22, 26, 37, 44)	35 (25, 29, 42, 50)	34 (24, 28, 38, 46)	36 (25, 31, 41, 48)
40-49	31 (22, 27, 36, 42)	32 (25, 28, 38, 45)	35 (25, 29, 43, 53)	33 (23, 28, 37, 46)
50-59	34 (25, 28, 42, 49)	30 (25, 27, 35, 42)	39 (28, 32, 47, 58)	34 (25, 28, 41, 49)
60-69	35 (28, 31, 43, 52)	31 (24, 28, 36, 49)	44 (30, 36, 55, 66)	37 (25, 31, 46, 58)
≥ 70	39 (28, 34, 45, 52)	37 (19, 27, 41, 51)	50 (33, 41, 63, 77)	42 (28, 34, 52, 66)

PAC: pressão arterial central. Valores descritos nos percentis 50 (10, 25, 75 e 90).

**Tabela 12:** – Valores de referência para VOP e AIx de acordo com sexo e idade na população normal e com fatores de risco cardiovascular **214**

Idade (anos)	População normal		População com fatores de risco cardiovascular	
	Feminino	Masculino	Feminino	Masculino
**Variável: VOP**				
< 30	4,9 (4,4, 4,5, 5,0, 5,3)	5,2 (4,9, 5,1, 5,4, 5,7)	5,3 (4,7, 5,0, 5,6, 6,0)	5,5 (5,0, 5,3, 5,8, 6,3)
30-39	5,4 (5,0, 5,2, 5,8, 6,1)	5,7 (5,3, 5,5, 5,9, 6,1)	5,8 (5,3, 5,5, 6,2, 6,7)	6,1 (5,5, 5,8, 6,4, 6,7)
40-49	6,4 (5,7, 6,0, 6,7, 6,9)	6,5 (5,9, 6,2, 6,8, 7,0)	6,8 (6,0, 6,4, 7,2, 7,7)	6,8 (6,2, 6,4, 7,1, 7,5)
50-59	7,5 (6,7, 7,0, 7,8, 8,2)	7,4 (6,9, 7,2, 7,9, 8,0)	7,9 (7,1, 7,5, 8,3, 8,8)	7,9 (7,1, 7,5, 8,3, 8,7)
60-69	8,9 (8,1, 8,5, 9,2, 9,4)	8,9 (8,2, 8,6, 9,1, 9,6)	9,3 (8,4, 8,8, 9,8, 10,4)	9,2 (8,4, 8,7, 9,7, 10,2)
≥ 70	11,3 (10,2, 10,4, 12,5, 13,2)	11,0 (10,1, 10,6, 11,6, 12,3)	11,8 (10,2, 10,8, 12,9, 14,0)	11,2 (9,9, 10,4, 12,1, 13,2)
**Variável: AIx**				
< 30	20 (11, 13, 27, 33)	16 (04, 10, 23, 27)	28 (11, 20, 34, 38)	16 (02, 08, 23, 30)
30-39	22 (12, 16, 28, 34)	14 (01, 07, 18, 24)	26 (11, 18, 32, 37)	15 (03, 09, 21, 27)
40-49	23 (09, 15, 29, 35)	15 (00, 06, 21, 25)	25 (10, 17, 34, 38)	15 (02, 08, 23, 30)
50-59	22 (07, 12, 33, 39)	12 (02, 04, 19, 22)	24 (08, 14, 33, 39)	15 (03, 07, 24, 32)
60-69	23 (09, 14, 34, 42)	17 (01, 05, 27, 43)	28 (11, 18, 37, 44)	17 (03, 09, 26, 34)
≥ 70	28 (11, 20, 39, 42)	22 (05, 10, 33, 41)	33 (17, 25, 42, 48)	22 (04, 12, 31, 41)

VOP: velocidade de onda de pulso; AIx: Augmentation Index. Valores descritos nos percentis 50 (10, 25, 75 e 90).

**Quadro 1:** – Sons de Korotkoff

FASES	SONS
Fase I	Aparecimento do primeiro som, que é fraco, seguido por batidas regulares, que corresponde à PA sistólica
Fase II	Sons suaves e longos como um murmúrio intermitente
Fase III	Sons tornam-se mais crispados
Fase IV	Sons diminuem de intensidade (abafamento)
Fase V	Desaparecimento dos sons que correspondem à PA diastólica

**Quadro 2:** – Desvantagens da técnica auscultatória em relação à técnica oscilométrica

• Arredondamento dos valores de PA para dígitos terminados em 0 ou 5
• Posicionamento incorreto dos olhos sobre o mostrador do manômetro aneroide
• Pressão excessiva do estetoscópio deformando a artéria
• Inflação excessiva do manguito causando dor
• Deflação rápida do manguito com subestimação da PA sistólica e/ou superestimação da diastólica
• Identificação incorreta dos sons da PA sistólica e diastólica
• O manômetro aneroide pode estar descalibrado, mesmo quando o ponteiro está no “zero”
• Não permite a medida desacompanhada da PA no consultório

PA: pressão arterial.

**Quadro 3:** – Medida da pressão arterial (PA) desacompanhada no consultório

Orientações
• Realizar em ambiente calmo, preferencialmente em uma sala isolada
• Orientar o paciente sobre a importância do procedimento
• Seguir os passos de preparo do paciente para a realização da medida com aparelhos automáticos (Quadro 4)
• Afastar todas as pessoas da sala
• Orientar para apertar o botão do aparelho que inicia as medidas quando estiver sozinho na sala
• Aguardar cerca de 1 minuto entre a realização das medidas
• Realizar três medidas consecutivas.
• Manter um folheto ilustrativo com orientações sobre o procedimento, em local visível

**Quadro 5:** – Como estimar o nível da pressão sistólica

Estimativa da PA sistólica
Posicionar o manguito ideal (Quadro 6) 2 a 3 cm acima da fossa cubital com o meio da bolsa inflável sobre a artéria braquial
Palpar a artéria radial
Fechar a válvula da pera e inflar até identificar o desaparecimento do pulso
Abrir a válvula lentamente, para desinflar o manguito
Identificar pelo método palpatório a PA sistólica (reaparecimento do pulso)

PA: pressão arterial.

**Quadro 6:** – Como medir a circunferência do braço

Medida da circunferência do braço
Posicionar o braço dobrado na altura da cintura
Medir a distância entre as duas proeminências ósseas do ombro (acrômio) e o cotovelo (olécrano) para determinar a porção mediana.
Estender o braço ao longo do corpo e medir a circunferência na região identificada como mediana
Selecionar o manguito ideal de acordo com o tamanho do braço. A largura da bolsa inflável deve corresponder a 37-50% da circunferência braquial, e seu comprimento envolver pelo menos 75-100% do braço (Quadro 7)
Não coloque o manguito sobre roupas

**Quadro 7:** – Dimensões do manguito de acordo com a circunferência do membro

Circunferência	Denominação do manguito	Largura da bolsa inflável do manguito	Comprimento da bolsa inflável do manguito
≤ 6 cm	Recém-nascido	3 cm	6 cm
6-15 cm	Criança	5 cm	15 cm
16-21 cm	Infantil	8 cm	21 cm
22-26 cm	Adulto pequeno	10 cm	24 cm
27-34 cm	Adulto	13 cm	30 cm
35-44 cm	Adulto grande	16 cm	38 cm
45-52 cm	Coxa	20 cm	42 cm

**Quadro 16:** – Atividades do profissional da saúde no momento da instalação do aparelho de MAPA

No dia da realização do exame, o profissional responsável pela instalação do monitor deve seguir as recomendações descritas a seguir:
***1.*** Explicar detalhadamente como será o exame e recomendar a manutenção das atividades habituais durante o período em que ele será realizado.
***2.*** Recomendar o seguimento da orientação médica quanto ao uso dos medicamentos.
***3.*** Orientar o paciente que não pratique exercícios físicos durante o período de realização do exame.
***4.*** Medir o peso e a estatura.
***5.*** Medir a circunferência do braço e selecionar o manguito com largura e comprimento adequados.
***6.*** Medir a PA na posição sentada, após 5 minutos de repouso, em ambos os braços, antes da instalação do aparelho pelo método auscultatório ou oscilométrico, preferencialmente de forma simultânea.
***7.*** Instalar o manguito no braço não dominante, se a diferença da PA sistólica for < 10 mmHg. Quando ≥ 10 mmHg, usar o manguito no braço com maior valor de PA.
***8.*** Posicionar o manguito de 2 a 3 cm acima da fossa cubital, seguindo a orientação específica do equipamento em uso.
***9.*** Programar o monitor seguindo as informações do paciente para a definição dos períodos de vigília e sono. Seguir as orientações estabelecidas no item “Protocolo para a realização do exame”.
***10.*** Após a colocação do equipamento, comparar a medida obtida pelo monitor de MAPA com a medida obtida pelo método auscultatório ou oscilométrico, certificando-se de que as diferenças não sejam superiores a 5 mmHg.
***11.*** Certificar-se de que o paciente compreendeu claramente todas as orientações e que está seguro para contribuir adequadamente para a realização do exame.
***12.*** Fazer, pelo menos, duas medidas de teste antes de liberar o paciente.

PA: pressão arterial; MAPA: monitoração ambulatorial da pressão arterial.

**Quadro 17:** – Atividades do profissional da saúde no momento da retirada do aparelho de MAPA


***1.*** Conferir o preenchimento do diário com o paciente, especialmente no que se refere a horários de tomada dos medicamentos, duração e qualidade do sono e relato dos “acontecimentos ocasionais”.
***2.*** Fazer, pelo menos, duas medidas de teste.
***3.*** Fazer a análise subjetiva da qualidade das atividades exercidas no período de monitoração como, por exemplo: se manteve atividades regulares, se sentiu limitação de suas atividades por incômodo com as insuflações, entre outros. Esses fatos devem ser considerados na interpretação e emissão do laudo.
***4.*** Verificar o número de leituras válidas durante a vigília e o sono e informar o paciente sobre a necessidade de repetir o exame.
***5.*** Transferir os dados do monitor para o computador e colocar informações como: peso, altura, medicações com dose e número de tomadas, horários das principais atividades (dormir, acordar, desjejum, almoço, jantar).

MAPA: monitoração ambulatorial da pressão arterial.
