# Supplementary material for: Diretrizes Brasileiras de Medidas da Pressão Arterial Dentro e Fora do Consultório – 2023
Source: Arq Bras Cardiol. 2024 Feb 23;121(4):e20240113. [Article in Portuguese] doi: 10.36660/abc.20240113 (PMC11186661; doi:10.36660/abc.20240113)
Supplement: Translation

# Translation: Brazilian Guidelines for In-office and Out-of-office Blood Pressure Measurement – 2023

**DOI:** 10.36660/abc.20240113i

**Authors:** Audes Diogenes de Magalhães Feitosa, Weimar Kunz Sebba Barroso, Decio Mion, Fernando Nobre, Marco Antonio Mota-Gomes, Paulo Cesar Brandão Veiga Jardim, Celso Amodeo, Adriana Camargo Oliveira, Alexandre Alessi, Ana Luiza Lima Sousa, Andréa Araujo Brandão, Andrea Pio-Abreu, Andrei C. Sposito, Angela Maria Geraldo Pierin, Annelise Machado Gomes de Paiva, Antonio Carlos de Souza Spinelli, Carlos Alberto Machado, Carlos Eduardo Poli-de-Figueiredo, Cibele Isaac Saad Rodrigues, Claudia Lucia de Moraes Forjaz, Diogo Pereira Santos Sampaio, Eduardo Costa Duarte Barbosa, Elizabete Viana de Freitas, Elizabeth do Espirito Santo Cestario, Elizabeth Silaid Muxfeldt, Emilton Lima, Erika Maria Gonçalves Campana, Fabiana Gomes Aragão Magalhães Feitosa, Fernanda Marciano Consolim-Colombo, Fernando Antônio de Almeida, Giovanio Vieira da Silva, Heitor Moreno, Helius Carlos Finimundi, Isabel Cristina Britto Guimarães, João Roberto Gemelli, José Augusto Soares Barreto-Filho, José Fernando Vilela-Martin, José Marcio Ribeiro, Juan Carlos Yugar-Toledo, Lucélia Batista Neves Cunha Magalhães, Luciano F. Drager, Luiz Aparecido Bortolotto, Marco Antonio de Melo Alves, Marcus Vinícius Bolívar Malachias, Mario Fritsch Toros Neves, Mayara Cedrim Santos, Nelson Dinamarco, Osni Moreira, Oswaldo Passarelli, Priscila Valverde de Oliveira Vitorino, Roberto Dischinger Miranda, Rodrigo Bezerra, Rodrigo Pinto Pedrosa, Rogerio Baumgratz de Paula, Rogério Toshiro Passos Okawa, Rui Manuel dos Santos Póvoa, Sandra C. Fuchs, Sandro Gonçalves de Lima, Sayuri Inuzuka, Sebastião Rodrigues Ferreira-Filho, Silvio Hock de Paffer Fillho, Thiago de Souza Veiga Jardim, Vanildo da Silva Guimarães, Vera Hermina Kalika Koch, Waléria Dantas Pereira Gusmão, Wille Oigman, Wilson Nadruz

**Affiliations:** 1 Brazil Universidade Federal de Pernambuco (UFPE), Recife, PE – Brazil; 2 Brazil Pronto Socorro Cardiológico de Pernambuco (PROCAPE), Recife, PE – Brazil; 3 Brazil Instituto de Assistência, Pesquisa e Ensino em Saúde (IAPES), Recife, PE – Brazil; 4 Brazil Faculdade de Medicina da Universidade Federal de Goiás (UFG), Goiânia, GO – Brazil; 5 Brazil Hospital das Clínicas da Faculdade de Medicina da Universidade de São Paulo (HCFMUSP), São Paulo, SP – Brazil; 6 Brazil Hospital das Clínicas da Faculdade de Medicina de Ribeirão Preto da Universidade de São Paulo, Ribeirão Preto, SP – Brazil; 7 Brazil Centro Universitário CESMAC, Maceió, AL – Brazil; 8 Brazil Hospital do Coração de Alagoas, Maceió, AL – Brazil; 9 Brazil Centro de Pesquisas Clínicas Dr. Marco Mota, Maceió, AL – Brazil; 10 Brazil Hcor, Associação Beneficente Síria, São Paulo, SP – Brazil; 11 Brazil Liga de Hipertensão Arterial da Universidade Federal de Goiás (UFG), Goiânia, GO – Brazil; 12 Brazil Universidade Federal do Paraná (UFPR), Curitiba, PR – Brazil; 13 Brazil Faculdade de Enfermagem da Universidade Federal de Goiás (UFG), Goiânia, GO – Brazil; 14 Brazil Universidade do Estado do Rio de Janeiro (UERJ), Rio de Janeiro, RJ – Brazil; 15 Brazil Universidade Estadual de Campinas (UNICAMP), Campinas, São Paulo – Brazil; 16 Brazil Universidade de São Paulo (USP), São Paulo, SP – Brazil; 17 Brazil Cardiocentro, Natal, RN – Brazil; 18 Brazil Secretaria Municipal de Saúde de Campos do Jordão, Campos do Jordão, SP – Brazil; 19 Brazil Pontifícia Universidade Católica do Rio Grande do Sul, Porto Alegre, RS – Brazil; 20 Brazil Pontifícia Universidade Católica de São Paulo, Faculdade de Ciências Médicas e da Saúde, Sorocaba, SP – Brazil; 21 Brazil Escola de Educação Física e Esporte da Universidade de São Paulo (USP), São Paulo, SP – Brazil; 22 Brazil Hospital das Clínicas da Universidade Federal de Goiás, Goiânia, GO – Brazil; 23 Brazil Universidade FEEVALE, Porto Alegre, RS – Brazil; 24 Brazil Sociedade Brasileira de Geriatria e Gerontologia, Rio de Janeiro, RJ – Brazil; 25 Brazil Centro Universitário de Votuporanga (UNIFEV), Votuporanga, SP – Brazil; 26 Brazil Universidade Federal do Rio de Janeiro (UFRJ), Hospital Universitário Clementino Fraga Filho – Programa de Hipertensão Arterial Resistente (ProHArt), Rio de Janeiro, RJ – Brazil; 27 Brazil Instituto de Educação Médica (IDOMED) - Universidade Estácio de Sá, Rio de Janeiro, RJ – Brazil; 28 Brazil Pontifícia Universidade Católica do Paraná, Curitiba, PR – Brazil; 29 Brazil Instituto de Medicina Integral Prof. Fernando Figueira (IMIP), Recife, PE – Brazil; 30 Brazil Instituto do Coração da Faculdade de Medicina da Universidade de São Paulo (Incor/FMUSP), São Paulo, SP – Brazil; 31 Brazil Universidade de Caxias do Sul (UCS), Caxias do Sul, RS – Brazil; 32 Brazil Universidade Federal da Bahia (UFBA), Salvador, BA – Brazil; 33 Brazil Governo do Estado de Rondônia, Porto Velho, RO – Brazil; 34 Brazil Universidade Federal de Sergipe, São Cristóvão, SE – Brazil; 35 Brazil Faculdade de Medicina de São José do Rio Preto (FAMERP), São José do Rio Preto, SP – Brazil; 36 Brazil Faculdade Ciências Médicas de Minas Gerais, Belo Horizonte, MG – Brazil; 37 Brazil Hospital Felício Rocho, Belo Horizonte, MG – Brazil; 38 Brazil Faculdade Zarns, Salvador, BA – Brazil; 39 Brazil Hospital Esperança Recife, Ilha do Leite, Recife, PE – Brazil; 40 Brazil Fundação Educacional Lucas Machado (FELUMA), Belo Horizonte, MG – Brazil; 41 Brazil Colegiado de Medicina – Universidade Estadual de Santa Cruz (UESC), Ilhéus, BA – Brazil; 42 Brazil Clínica Gapski Moreira, Curitiba, PR – Brazil; 43 Brazil Instituto Dante Pazzanese de Cardiologia, São Paulo, SP – Brazil; 44 Brazil Pontifícia Universidade Católica de Goiás, Goiânia, GO – Brazil; 45 Brazil Escola Paulista de Medicina, Universidade Federal de São Paulo (UNIFESP), São Paulo, SP – Brazil; 46 Brazil Laboratório de Imunopatologia Keizo Asami da Universidade Federal de Pernambuco, Recife, PE – Brazil; 47 Brazil Universidade Federal de Juiz de Fora, Juiz de Fora, MG – Brazil; 48 Brazil Universidade Estadual de Maringá, Maringá, PR – Brazil; 49 Brazil Universidade Federal do Rio Grande do Sul (UFRGS), Porto Alegre, RS – Brazil; 50 Brazil Unidade de Hipertensão Arterial – NIPEE – LHA/UFG, Goiânia, GO – Brazil; 51 Brazil Nefroclinica de Uberlândia, Uberlândia, MG – Brazil; 52 Brazil Faculdade de Medicina de Olinda, Olinda, PE – Brazil; 53 Brazil Hospital do Coração de Goiás, Goiânia, GO – Brazil; 54 Brazil Diagnóstico Cardíaco, Recife, PE – Brazil; 55 Brazil Instituto da Criança e do adolescente do Hospital das Clínicas da Faculdade de Medicina da Universidade de São Paulo (HCFMUSP), São Paulo, SP – Brazil; 56 Brazil Universidade Estadual de Ciências da Saúde de Alagoas (UNCISAL), Maceió, AL – Brazil

**Table:** 

Brazilian Guidelines for In-office and Out-of-office Blood Pressure Measurement – 2023	
The report below lists declarations of interest as reported to the SBC by the experts during the period of the development of these statement, 2021-2023.	
Expert	Type of relationship with industry
Adriana Camargo Oliveira	Financial declaration A - Economically relevant payments of any kind made to (i) you, (ii) your spouse/partner or any other person living with you, (iii) any legal person in which any of these is either a direct or indirect controlling owner, business partner, shareholder or participant; any payments received for lectures, lessons, training instruction, compensation, fees paid for participation in advisory boards, investigative boards or other committees, etc. From the brazilian or international pharmaceutical, orthosis, prosthesis, equipment and implants industry: - Brace Pharma; EMS; Servier; Biolab. Other relationships Funding of continuing medical education activities, including travel, accommodation and registration in conferences and courses, from the brazilian or international pharmaceutical, orthosis, prosthesis, equipment and implants industry: - Biolab: Bivolet; EMS: Xakilis.
Alexandre Alessi	Nothing to be declared
Ana Luiza Lima Sousa	Nothing to be declared
Andréa Araujo Brandão	Nothing to be declared
Andrea Pio-Abreu	Nothing to be declared
Andrei C. Sposito	Other relationships Funding of continuing medical education activities, including travel, accommodation and registration in conferences and courses, from the brazilian or international pharmaceutical, orthosis, prosthesis, equipment and implants industry: - Novo Nordisk; Novartis.
Angela Maria Geraldo Pierin	Nothing to be declared
Annelise Machado Gomes de Paiva	Nothing to be declared
Antonio Carlos de Souza Spinelli	Financial declaration A - Economically relevant payments of any kind made to (i) you, (ii) your spouse/partner or any other person living with you, (iii) any legal person in which any of these is either a direct or indirect controlling owner, business partner, shareholder or participant; any payments received for lectures, lessons, training instruction, compensation, fees paid for participation in advisory boards, investigative boards or other committees, etc. From the brazilian or international pharmaceutical, orthosis, prosthesis, equipment and implants industry: - Novonordisk: Ozempic; Daiichi Sankyo: Benicar Triplo; Torrent: Rosucor; Boehringer: Jardiance. Other relationships Funding of continuing medical education activities, including travel, accommodation and registration in conferences and courses, from the brazilian or international pharmaceutical, orthosis, prosthesis, equipment and implants industry: - Novonordisk: Ozempic; Daiichi Sankyo: Benicar Triplo; Torrent: Rosucor; Boehringer: Jardiance.
Audes Diogenes de Magalhães Feitosa	Financial declaration A - Economically relevant payments of any kind made to (i) you, (ii) your spouse/partner or any other person living with you, (iii) any legal person in which any of these is either a direct or indirect controlling owner, business partner, shareholder or participant; any payments received for lectures, lessons, training instruction, compensation, fees paid for participation in advisory boards, investigative boards or other committees, etc. From the brazilian or international pharmaceutical, orthosis, prosthesis, equipment and implants industry: - Omron Consultant; classes for Omron, Micromed and Cardios. Other relationships Funding of continuing medical education activities, including travel, accommodation and registration in conferences and courses, from the brazilian or international pharmaceutical, orthosis, prosthesis, equipment and implants industry: - Omron.
Carlos Alberto Machado	Nothing to be declared
Carlos Eduardo Poli-de-Figueiredo	Financial declaration A - Economically relevant payments of any kind made to (i) you, (ii) your spouse/partner or any other person living with you, (iii) any legal person in which any of these is either a direct or indirect controlling owner, business partner, shareholder or participant; any payments received for lectures, lessons, training instruction, compensation, fees paid for participation in advisory boards, investigative boards or other committees, etc. From the brazilian or international pharmaceutical, orthosis, prosthesis, equipment and implants industry: - AstraZeneca/Alexion: HUS and Chronic kidney disease; Bayer: Chronic kidney disease; Fresenius, Life and Baxter: Dialysis. Organization of the MAYOPUCRS 2023 event and other congresses of Medical Societies with support from companies. No conflicts in the area of hypertension. B - Research funding under your direct/personal responsibility (directed to the department or institution) from the Brazilian or international pharmaceutical, orthosis, prosthesis, equipment and implants industry: - Researcher at the PUCRS Clinical Research Center. No studies at the moment. Outros relacionamentos Any economically relevant equity interest in companies in the healthcare or education industry or in any companies competing with or supplying to SBC: - Doctor's office (Nefromonitoriza). Participation in procurement committees for supplies or drugs in health institutions or any similar roles taken: - Head of Nephrology Service at HSL/PUCRS until 2023. Participation in government-related regulatory authorities or advocacy authorities in cardiology: - Participation in studies funded by the Ministry of Health, CNPq and CAPES.
Celso Amodeo	Nothing to be declared
Cibele Isaac Saad Rodrigues	Nothing to be declared
Claudia Lucia de Moraes Forjaz	Nothing to be declared
Décio Mion Junior	Nothing to be declared
Diogo Pereira Santos Sampaio	Nothing to be declared
Eduardo Costa Duarte Barbosa	Financial declaration A - Economically relevant payments of any kind made to (i) you, (ii) your spouse/partner or any other person living with you, (iii) any legal person in which any of these is either a direct or indirect controlling owner, business partner, shareholder or participant; any payments received for lectures, lessons, training instruction, compensation, fees paid for participation in advisory boards, investigative boards or other committees, etc. From the brazilian or international pharmaceutical, orthosis, prosthesis, equipment and implants industry: - Cardios: Arteris; Brace Pharma: Olmy Anlo; Servier: Triplixam; EMS: Bramicar. Other relationships Funding of continuing medical education activities, including travel, accommodation and registration in conferences and courses, from the brazilian or international pharmaceutical, orthosis, prosthesis, equipment and implants industry: - Servier: Triplixam.
Elizabete Viana de Freitas	Nothing to be declared
Elizabeth do Espírito Santo Cestario	Financial declaration A - Economically relevant payments of any kind made to (i) you, (ii) your spouse/partner or any other person living with you, (iii) any legal person in which any of these is either a direct or indirect controlling owner, business partner, shareholder or participant; any payments received for lectures, lessons, training instruction, compensation, fees paid for participation in advisory boards, investigative boards or other committees, etc. From the brazilian or international pharmaceutical, orthosis, prosthesis, equipment and implants industry: - Servier: Triplixam; Torrent: Rosucor. B - Research funding under your direct/personal responsibility (directed to the department or institution) from the Brazilian or international pharmaceutical, orthosis, prosthesis, equipment and implants industry: - Servier: Vastarel; Libbs: Hipertensão. Other relationships Funding of continuing medical education activities, including travel, accommodation and registration in conferences and courses, from the brazilian or international pharmaceutical, orthosis, prosthesis, equipment and implants industry: - Servier; Torrent.
Elizabeth Silaid Muxfeldt	Nothing to be declared
Emilton Lima Júnior	Financial declaration A - Economically relevant payments of any kind made to (i) you, (ii) your spouse/partner or any other person living with you, (iii) any legal person in which any of these is either a direct or indirect controlling owner, business partner, shareholder or participant; any payments received for lectures, lessons, training instruction, compensation, fees paid for participation in advisory boards, investigative boards or other committees, etc. From the brazilian or international pharmaceutical, orthosis, prosthesis, equipment and implants industry: - Servier: Hypertension; Novo Nordisk: Diabetes/Obesity; Daiichi Sankyo: Liliana; Chiesi: COPD; Novartis: Dyslipidemia. Other relationships Funding of continuing medical education activities, including travel, accommodation and registration in conferences and courses, from the brazilian or international pharmaceutical, orthosis, prosthesis, equipment and implants industry: - Servier: Hypertension; Novo Nordisk: Diabetes/Obesity; Daiichi Sankyo: Liliana; Chiesi: COPD; Novartis: Dyslipidemia.
Erika Maria Gonçalves Campana	Financial declaration A - Economically relevant payments of any kind made to (i) you, (ii) your spouse/partner or any other person living with you, (iii) any legal person in which any of these is either a direct or indirect controlling owner, business partner, shareholder or participant; any payments received for lectures, lessons, training instruction, compensation, fees paid for participation in advisory boards, investigative boards or other committees, etc. From the brazilian or international pharmaceutical, orthosis, prosthesis, equipment and implants industry: - Servier: Perindopril/Hypertension; Biolab: Pressplus/Hypertension. Other relationships Funding of continuing medical education activities, including travel, accommodation and registration in conferences and courses, from the brazilian or international pharmaceutical, orthosis, prosthesis, equipment and implants industry: - Servier: Perindopril/Hypertension.
Fabiana Gomes Aragão Magalhães Feitosa	Nothing to be declared
Fernanda M. Consolim-Colombo	Financial declaration A - Economically relevant payments of any kind made to (i) you, (ii) your spouse/partner or any other person living with you, (iii) any legal person in which any of these is either a direct or indirect controlling owner, business partner, shareholder or participant; any payments received for lectures, lessons, training instruction, compensation, fees paid for participation in advisory boards, investigative boards or other committees, etc. From the brazilian or international pharmaceutical, orthosis, prosthesis, equipment and implants industry: - Daiichi Sankyo; Merck; Servier; AstraZeneca. Other relationships Funding of continuing medical education activities, including travel, accommodation and registration in conferences and courses, from the brazilian or international pharmaceutical, orthosis, prosthesis, equipment and implants industry: - Daiichi Sankyo; Servier.
Fernando Antônio de Almeida	Nothing to be declared
Fernando Nobre	Nothing to be declared
Giovanio Vieira da Silva	Nothing to be declared
Heitor Moreno Júnior	Nothing to be declared
Helius Carlos Finimundi	Nothing to be declared
Isabel Cristina Britto Guimarães	Nothing to be declared
João Roberto Gemelli	Financial declaration A - Economically relevant payments of any kind made to (i) you, (ii) your spouse/partner or any other person living with you, (iii) any legal person in which any of these is either a direct or indirect controlling owner, business partner, shareholder or participant; any payments received for lectures, lessons, training instruction, compensation, fees paid for participation in advisory boards, investigative boards or other committees, etc. From the brazilian or international pharmaceutical, orthosis, prosthesis, equipment and implants industry: - Servier; Boerinhger; AstraZeneca; Libbs.
José Augusto Soares Barreto Filho	Nothing to be declared
José Fernando Vilela-Martin	Nothing to be declared
José Marcio Ribeiro	Nothing to be declared
Juan Carlos Yugar-Toledo	Nothing to be declared
Lucélia Batista Neves Cunha Magalhães	Other relationships Funding of continuing medical education activities, including travel, accommodation and registration in conferences and courses, from the brazilian or international pharmaceutical, orthosis, prosthesis, equipment and implants industry: - AstraZeneca: Selozok; Farmoquímica: Exforge.
Luciano Ferreira Drager	Nothing to be declared
Luiz Aparecido Bortolotto	Financial declaration A - Economically relevant payments of any kind made to (i) you, (ii) your spouse/partner or any other person living with you, (iii) any legal person in which any of these is either a direct or indirect controlling owner, business partner, shareholder or participant; any payments received for lectures, lessons, training instruction, compensation, fees paid for participation in advisory boards, investigative boards or other committees, etc. From the brazilian or international pharmaceutical, orthosis, prosthesis, equipment and implants industry: - GSK: Shingrix; Servier: Triplicam.
Marco Antonio de Melo Alves	Other relationships Funding of continuing medical education activities, including travel, accommodation and registration in conferences and courses, from the brazilian or international pharmaceutical, orthosis, prosthesis, equipment and implants industry: - Torrent: Dyslipidemia/Hypertension.
Marco Antonio Mota-Gomes	Financial declaration A - Economically relevant payments of any kind made to (i) you, (ii) your spouse/partner or any other person living with you, (iii) any legal person in which any of these is either a direct or indirect controlling owner, business partner, shareholder or participant; any payments received for lectures, lessons, training instruction, compensation, fees paid for participation in advisory boards, investigative boards or other committees, etc. From the brazilian or international pharmaceutical, orthosis, prosthesis, equipment and implants industry: - Omron: Equipment; Biolab: Press Plus; Daiichi Sankyo: Benicar Triplo; AstraZeneca: Forxiga; Ache: Edistride. B - Research funding under your direct/personal responsibility (directed to the department or institution) from the Brazilian or international pharmaceutical, orthosis, prosthesis, equipment and implants industry: - Novartis: Inclisiran; Libbs: Associação Tripla; Servier: Associação Quádrupla.
Marcus Vinícius Bolívar Malachias	Financial declaration A - Economically relevant payments of any kind made to (i) you, (ii) your spouse/partner or any other person living with you, (iii) any legal person in which any of these is either a direct or indirect controlling owner, business partner, shareholder or participant; any payments received for lectures, lessons, training instruction, compensation, fees paid for participation in advisory boards, investigative boards or other committees, etc. From the brazilian or international pharmaceutical, orthosis, prosthesis, equipment and implants industry: - Boehringer Ingelheim/Lilly: diabetes; Daiichi Sankyo: Hypertension; Libbs: Hypertension, Roche: Biomarkers; Novo Nordisk: Diabetes; Bayer: Diabetes; Novartis: Heart Failure; Viatris: Heart Failure; Servier: Heart Failure. Other relationships Funding of continuing medical education activities, including travel, accommodation and registration in conferences and courses, from the brazilian or international pharmaceutical, orthosis, prosthesis, equipment and implants industry: - Boehringer Ingelheim/Lilly: Diabetes; Daiichi Sankyo: Hypertension; Libbs: Hypertension, Novo Nordisk: Diabetes; Bayer: Diabetes. Any economically relevant equity interest in companies in the healthcare or education industry or in any companies competing with or supplying to SBC: - Institute of Arterial Hypertension of Minas Gerais: health services; Cardio Check Up: services in the health sector.
Mario Fritsch Toros Neves	Nothing to be declared
Mayara Cedrim Santos	Nothing to be declared
Nelson Dinamarco	Nothing to be declared
Osni Moreira Filho	Financial declaration A - Economically relevant payments of any kind made to (i) you, (ii) your spouse/partner or any other person living with you, (iii) any legal person in which any of these is either a direct or indirect controlling owner, business partner, shareholder or participant; any payments received for lectures, lessons, training instruction, compensation, fees paid for participation in advisory boards, investigative boards or other committees, etc. From the brazilian or international pharmaceutical, orthosis, prosthesis, equipment and implants industry: - Daiichi Sankyo: Benicar Triplo; Servier: Triplixam. Other relationships Funding of continuing medical education activities, including travel, accommodation and registration in conferences and courses, from the brazilian or international pharmaceutical, orthosis, prosthesis, equipment and implants industry: - Torrent: Arterial Hypertension; Servier: Arterial Hypertension.
Oswaldo Passarelli Júnior	Nothing to be declared
Paulo Cesar Brandão Veiga Jardim	Nothing to be declared
Priscila Valverde de Oliveira Vitorino	Nothing to be declared
Roberto Dischinger Miranda	Other relationships Funding of continuing medical education activities, including travel, accommodation and registration in conferences and courses, from the brazilian or international pharmaceutical, orthosis, prosthesis, equipment and implants industry: - Novo Nordisk: Ozempic; EMS: Arterial Hypertension and Atrial Fibrillation.
Rodrigo Bezerra	Nothing to be declared
Rodrigo Pinto Pedrosa	Nothing to be declared
Rogério Baumgratz de Paula	Nothing to be declared
Rogério Toshiro Passos Okawa	Financial declaration A - Economically relevant payments of any kind made to (i) you, (ii) your spouse/partner or any other person living with you, (iii) any legal person in which any of these is either a direct or indirect controlling owner, business partner, shareholder or participant; any payments received for lectures, lessons, training instruction, compensation, fees paid for participation in advisory boards, investigative boards or other committees, etc. From the brazilian or international pharmaceutical, orthosis, prosthesis, equipment and implants industry: - EMS: Hypertension. Other relationships Funding of continuing medical education activities, including travel, accommodation and registration in conferences and courses, from the brazilian or international pharmaceutical, orthosis, prosthesis, equipment and implants industry: - Novo Nordisk: Obesity.
Rui Manuel dos Santos Povoa	Nothing to be declared
Sandra C. Fuchs	Nothing to be declared
Sandro Gonçalves de Lima	Nothing to be declared
Sayuri Inuzuka	Other relationships Funding of continuing medical education activities, including travel, accommodation and registration in conferences and courses, from the brazilian or international pharmaceutical, orthosis, prosthesis, equipment and implants industry: - Servier; EMS; Daichi Sankyo.
Sebastião Rodrigues Ferreira-Filho	Nothing to be declared
Silvio Hock de Paffer Fillho	Nothing to be declared
Thiago de Souza Veiga Jardim	Nothing to be declared
Vanildo da Silva Guimarães Neto	Nothing to be declared
Vera Hermina Kalika Koch	Nothing to be declared
Waléria Dantas Pereira Gusmão	Nothing to be declared
Weimar Kunz Sebba Barroso	Financial declaration A - Economically relevant payments of any kind made to (i) you, (ii) your spouse/partner or any other person living with you, (iii) any legal person in which any of these is either a direct or indirect controlling owner, business partner, shareholder or participant; any payments received for lectures, lessons, training instruction, compensation, fees paid for participation in advisory boards, investigative boards or other committees, etc. From the brazilian or international pharmaceutical, orthosis, prosthesis, equipment and implants industry: - EMS; Brace Pharma; Servier; Biolab; Omron; Cardios. B - Research funding under your direct/personal responsibility (directed to the department or institution) from the Brazilian or international pharmaceutical, orthosis, prosthesis, equipment and implants industry: - Ministry of Health; PROADI SUS; Novartis: Entresto. C - Personal research funding paid by the Brazilian or international pharmaceutical, orthosis, prosthesis, equipment and implants industry: - EMS: RITMO Study; Brace Pharma: BRACE 23 Study. Other relationships Funding of continuing medical education activities, including travel, accommodation and registration in conferences and courses, from the brazilian or international pharmaceutical, orthosis, prosthesis, equipment and implants industry: - Servier; European Hypertension Meeting.
Wille Oigman	Nothing to be declared
Wilson Nadruz Junior	Nothing to be declared

## Table of contents

Updates and modifications to the Brazilian Guidelines for In-Office and Out-of-Office Blood Pressure Measurement 10

Part 1 – Blood pressure measurement 10

1. Introduction 10

2. In-office BP measurement 11

2.1. Auscultatory BP measurement 11

2.2. Oscillometric BP measurement with automated and semiautomated devices 11

2.3. Steps for in-office BP measurement 11

2.4. Wrist and finger BP measurement 12

2.5. BP measurement in older patients 13

2.6. BP classification according to in-office measurements 13

3. Out-of-office BP measurement 13

3.1. Self-measured blood pressure (SMBP) 13

3.2. BP measurement in pharmacies 14

3.3. BP measurement in public spaces 14

3.4 Ambulatory blood pressure monitoring (ABPM) 14

3.5. Home blood pressure monitoring (HBPM) 14

4. Central BP measurement and derived parameters 14

5. BP measurement during exercise 14

Part 2 – Aspects and concepts common to ABPM and HBPM 15

1. Essential aspects for establishing an ABPM and HBPM service 15

2. Abnormality thresholds for in-office and out-of-office BP measurements 15

2.1. Normotension and controlled hypertension 15

2.2. Sustained hypertension and sustained uncontrolled hypertension 16

2.3. White-coat hypertension and white-coat uncontrolled hypertension 16

**2.3.1. White-coat effect** 16

**2.3.2. Investigation** 16

**2.3.3. Prognosis** 16

**2.3.4. Follow-up and treatment** 16

2.4. Masked hypertension and masked uncontrolled hypertension 17

**2.4.1. Masking effect** 17

**2.4.2. Investigation** 17

**2.4.3. Prognosis** 17

**2.4.4. Treatment** 17

2.5 Resistant hypertension 17

3. Indications, advantages, and disadvantages of ABPM and HBPM 18

Part 3 – Ambulatory blood pressure monitoring (ABPM) 18

1. Definition 18

2. ABPM protocols 18

2.1. ABPM reproducibility 18

3. Guidance for patients and health professionals 18

3.1. Activity diary during BP measurements 18

3.2. General instructions 18

4. Interpretation of results 19

4.1. Criteria for interpreting BP measurements 19

4.2. Abnormality thresholds 19

4.3. BP patterns in transition periods between daytime and nighttime 19

4.4. Morning BP surge 22

4.5. BP load, areas under the curves, BP variability, and heart rate 22

**4.5.1. BP load** 22

**4.5.2. Areas under the curves** 22

**4.5.3. BP variability** 23

**4.5.4. Heart rate** 23

5. Interpretation of ABPM and technical report 23

5.1. Technical report 23

5.2. Systolic and diastolic BP patterns over 24 hours, daytime, and nighttime 23

5.3. Systolic and diastolic BP patterns in transition periods between daytime and nighttime 23

5.4. BP spikes and hypotension 23

5.5. Correlation between activities, medication use, and symptoms 23

5.6. Conclusion 24

6. Clinical applications of ABPM 25

6.1. Assessment of prognosis in patients with hypertension 25

6.2. Assessment of antihypertensive treatment efficacy 25

7. ABPM in special situations 25

7.1. Children and adolescents 25

7.2. Pregnant women 25

7.3. Older people 26

7.4. Diabetes 26

7.5. Chronic kidney disease 26

7.6. Obstructive sleep apnea syndrome 26

7.7 Heart failure 26

7.8. Physical activity 27

8. Cost-effectiveness 27

9. Perspectives 27

Part 4 – Home blood pressure monitoring (HBPM) 27

1. Introduction 27

2. Instructions for patients 27

3. HBPM protocol 28

4. Abnormality thresholds 28

5. Technical report and interpretation of results 28

6. Applications of HBPM 30

6.1. Determination of in-office and out-of-office BP behavior 30

6.2. Assessment of prognosis 30

6.3. Assessment of antihypertensive treatment 30

6.4. In special populations and situations 31

**6.4.1. Children and adolescents** 31

**6.4.2. Pregnant women** 31

**6.4.3. Older patients** 31

**6.4.4. Diabetes** 31

**6.4.5. Chronic kidney disease** 31

**6.4.6. Obesity** 32

**6.4.7. Arrhythmias** 32

7. Cost-effectiveness 32

8. Perspectives 32

Part 5 – Central blood pressure, pulse wave velocity, and augmentation index 32

1. Introduction 32

2. Definitions 32

2.1. Pulse wave velocity 32

2.2. Augmentation index 33

2.3. Central blood pressure 33

3. Indications 33

4. Advantages of measuring CBP, AIx, and PWV 33

5. Limitations of measuring CBP, AIx, and PWV 33

6. Techniques available for checking central and

arterial stiffness parameters 33

6.1. Methods for indirect measurement of CBP 33

7. Protocols for CBP, PWV, and AIx measurements 33

7.1. Protocol for measuring central parameters using tonometry 33

7.2. Protocol for the triple-trigger procedure (in-office measurement) using the oscillometric method 33

7.3. Protocol for 24-hour central BP parameter measurements using the oscillometric method 34

8. Reference values 34

8.1 Reference values for PWV 34

8.2. Reference values for cSBP 34

8.3. Reference values for PWV, CBP, and AIx using the oscillometric method in the Brazilian population 34

9. Prognostic value of BP measurements derived from central parameters 34

10. Measurement of central parameters over 24 hours 37

11. Perspectives 38

References 39

## Updates and modifications to the Brazilian Guidelines for In-Office and Out-of-Office Blood Pressure Measurement

These Guidelines revise and update the previous Guidelines for Ambulatory Blood Pressure Monitoring (ABPM) and Home Blood Pressure Monitoring (HBPM). Several relevant modifications have been made, focusing on fundamental aspects of in-office and out-of-office blood pressure (BP) measurement and monitoring, including ABPM, HBPM, self-measured blood pressure, and central blood pressure (CBP) measurement. Some of the main modifications are described below:

1. The update of the 6th Brazilian ABPM Guidelines and the 4th Brazilian HBPM Guidelines, published in 2018, is now called “Brazilian Guidelines for In-Office and Out-of-Office Blood Pressure Measurement”. This updated nomenclature reflects the current scope and relevance of these Guidelines, highlighting their substantial contribution to clinical practice.
2. Emphasis is placed on the accuracy and quality of in-office BP measurement.
3. New recommendations are provided on unattended automated in-office BP measurement.
4. A rigorous assessment of postural hypotension is described.
5. The use of BP measurements in pharmacies and public spaces to screen for hypertension is expanded.
6. The clinical indications for in-office and out-of-office BP measurements are updated.
7. The importance of BP measurements during physical exercise is acknowledged.
8. Mandatory certification and validation procedures are stipulated for monitors, according to www.stridebp.org and INMETRO.
9. Normality threshold values for HBPM are updated.
10. New behaviors identified in patients receiving antihypertensive treatment are described.
11. The flowchart for the assessment and management of masked and white-coat hypertension is revised.
12. The indications, limitations, advantages, and disadvantages of ABPM and HBPM are updated.
13. A new classification of normal BP values obtained by ABPM in children and adolescents is introduced.
14. The HBPM protocol is updated.
15. Updated values are introduced for determining white-coat and masking effects on HBPM.
16. A new HBPM protocol is provided for patients undergoing hemodialysis.
17. A new chapter on CBP, pulse wave velocity (PWV), and augmentation index (AIx) is introduced, which addresses possible indications, definition of specific protocols, and reference values for PWV, CBP, and AIx measurements.

These modifications support the commitment to diagnostic accuracy and improvement of BP care, promoting clinical practices that are based on and aligned with the most recent advances in the area.

## Part 1 – Blood pressure measurement

### 1. Introduction

Hypertension is one of the primary modifiable risk factors for morbidity and mortality worldwide. It ranks among the leading risk factors for conditions such as coronary artery disease (CAD), stroke, and renal failure. Moreover, it is highly prevalent, affecting over a third of the world’s population.^1 - 9^

Blood pressure (BP) measurement is an ESSENTIAL procedure in any medical setting and can be performed by different health care professionals. However, it is still commonly performed without the necessary technical care.^1 - 9^The diagnosis of hypertension relies on accurate BP readings, emphasizing the need for meticulous attention to measurement techniques, methods, and the use of adequate equipment.^1 - 9^

Following a diagnosis of hypertension, the course of action, including short-, medium-, and long-term treatments, is decided based on the results of BP measurements. Thus, poor technique and/or the use of inadequate equipment can lead to misdiagnosis, with either underestimation or overestimation of BP values, which in turn can lead to ineffective management, potentially causing significant harm to the health and economies of patients and nations.^1 - 11^

As recognition of the importance of adequate treatment increases, characterized by the adoption of more detailed normality thresholds and meticulous treatment objectives aimed at achieving stringent BP goals, the significance of taking accurate BP measurements becomes increasingly evident.^1 - 11^

BP measurement is commonly performed at the doctor’s office using the traditional method, and this is referred to as “in-office BP measurement”. Over the years, alternative methods have emerged, allowing patients to use semiautomated or automated monitors to measure BP in the waiting room or out of the office, such as in their homes or public spaces. A notable advancement occurred with the introduction of memory-equipped semiautomated monitors that take sequential out-of-office measurements (self-measured blood pressure [SMBP] and home blood pressure monitoring [HBPM]) and automated monitors that take programmed measurements for longer periods of time (ambulatory blood pressure monitoring [ABPM]).^1 , 2 , 4 , 12 , 13^

Some factors related to BP measurement can affect the accuracy of results, potentially complicating decision-making regarding the appropriate approach. These include the importance of using average values, BP variability throughout the day, and short-term BP variability. As a result, an increased frequency of measurements at different times of the day has been encouraged, and various guidelines have recommended the use of devices that facilitate this process. The use of HBPM and ABPM monitors is becoming increasingly common. In addition to providing more accurate measurements, they can identify white-coat hypertension (WCH), masked hypertension (MH), variations in BP during sleep, and resistant hypertension (RH), as defined in Chapter 2 of these Guidelines, if used together.^1 - 9 , 14 - 20^

However, it should be noted that, despite these advancements, diagnosis, classification, and goal-setting still primarily rely on in-office BP measurements. Therefore, the utmost importance should be given to this procedure.^15 , 16 , 21^

## 2. In-office BP measurement

### 2.1. Auscultatory BP measurement2,12,22-25

- The mercury sphygmomanometer is still considered the reference standard device for BP measurement, in addition to being useful for validating oscillometric and aneroid devices. However, in Brazil, its use in the health care setting has been banned by the Ministry of Labor (Regulatory Standard 15 125.001-9/I4).
- In many countries, mercury devices have been largely replaced by aneroid manometers. However, they are easily damaged and require frequent recalibration (at least every 12 months) to ensure their accuracy.
- Auscultatory BP measurement remains a common practice, with the use of aneroid sphygmomanometers and stethoscopes.
- The sphygmomanometer consists of a cuff, an inflatable rubber bladder, a manometer for BP measurement, and a system of valves, tubes, and an inflation bulb.
- The cuff is placed over the brachial artery and inflated, interrupting blood flow. The cuff is then gradually deflated and, as cuff pressure is lowered further, pressure generated by left ventricular contraction will pump blood into the artery and characteristic sounds known as Korotkoff sounds (Chart 1) will be heard through the stethoscope.
- The auscultatory technique requires a combination of good hearing and the ability to visually track the manometer’s values to accurately identify sounds corresponding to systolic and diastolic blood pressure.

### 2.2. Oscillometric BP measurement with automated and semiautomated devices4,13,24-27

- Oscillometric BP measurement with automated or semiautomated monitors offers several advantages over the auscultatory method, mainly by eliminating or reducing observer errors and systematic rounding errors, as well as poor technique (Chart 2).
- Another advantage of these devices is that patients can take their own BP measurements without the presence of a health care professional; this is referred to as “unattended automated office BP measurement” (Chart 3). This approach improves measurement reproducibility and helps minimize the white-coat effect.
- Automated and semiautomated monitors should be validated by specific protocols. A list of validated devices can be found at the following websites: British and Irish Hypertension Society (www.bihsoc.org/bp-monitors); European Society of Hypertension – International Society of Hypertension – World Hypertension League (www.stridebp.org); and Hypertension Canada (www.hypertension.ca/bpdevices). Most devices available on the market have not been validated.
- All devices, including those validated for use in adults, should be validated for use in special populations, including children, pregnant women, older adults, patients with an arm circumference greater than 42 cm, and patients with arrhythmias.
- Automated and semiautomated monitors should be calibrated every 12 months.
- Cuffs of different dimensions should be from the same manufacturer and model as the device.

### 2.3. Steps for in-office BP measurement 1 , 10 , 11 , 16 , 28 , 29

Special care should be taken to correctly prepare the patient and execute the procedure when taking BP measurements using either the auscultatory or oscillometric method, as detailed in Chart 4 .

**Chart 4:** – Steps for office BP measurement

Patient preparation
Have the patient rest for 5 minutes in a calm and comfortable environment and advise him/her not to talk or move during measurements
Make sure the patient has: • Emptied his/her bladder • Not practiced physical activity in the past 90 minutes • Not smoked or consumed alcohol, coffee, or food in the past 30 minutes
Have the patient sit with the feet flat on the floor and legs uncrossed, back relaxed and supported by chair
Position the patient’s arm at the heart level, supported, with the palm facing up and remove any restrictive clothing around the patient’s arm
If this is the patient’s first visit, record BP in both arms (preferably simultaneously), then use the arm with the higher reading for all measurements. Record which arm should be used for BP measurement
**Measurement steps**
Wrap the cuff snugly around the arm, 2 to 3 cm above the antecubital fossa, positioning the center of the cuff bladder directly over the brachial artery
Estimate systolic BP level* (Chart 5)
Palpate the brachial artery on the antecubital fossa and place the stethoscope’s bell or diaphragm over it without applying too much pressure.* Do not place the stethoscope over the cuff
Rapidly inflate the cuff to 20-30 mm Hg greater than the estimated systolic value*
Slowly deflate the cuff (approximately 2 mm Hg/s)*
Determine systolic BP upon hearing the first sound (Korotkoff phase I)
Determine diastolic BP upon disappearance of sounds (Korotkoff phase V)*
Continue to auscultate as you deflate the cuff for another 20-30 mm Hg below the last sound to confirm it has disappeared, then proceed to complete and rapid deflation. If the tapping sound persists after the pointer has reached the zero mark, determine diastolic BP upon muffling of sounds (Korotkoff phase IV) and record zero/diastolic/systolic BP values*
Take 3 measurements, 1 minute apart, and use the average of the last 2 measurements. If there is a difference of > 10 mm Hg, take more measurements.

BP: blood pressure. *Applied to the auscultatory technique

Assessing postural hypotension is an important part of patient evaluation, particularly for older adults, those with dysautonomia, and patients taking antihypertensive medication. In this case, BP should be measured after the patient has been in the supine position for 5 minutes, and then after the patient has been in the standing position for 1 minute and again after 3 minutes. When measuring standing BP, the patient’s arm should preferably be supported by the examiner at the heart level. Postural hypotension is defined as a drop of at least 20 mm Hg in systolic BP or a drop of at least 10 mm Hg in diastolic BP in the first and/or third minute after standing from a supine position.

### 2.4. Wrist and finger BP measurement

As previously discussed, the most commonly used and validated devices for BP measurement consist of an inflatable arm cuff used with either the auscultatory or oscillometric method, the latter including semiautomated and automated monitors. However, some devices are designed to wrap the cuff around the wrist, and these may also be used with the oscillometric method (automated devices). Compared with other devices, wrist BP monitors are smaller, easier to carry, and have been increasingly used by patients for out-of-office BP monitoring. These monitors are particularly indicated for patients with severe obesity, as errors in arm BP measurement and difficulty finding appropriately sized cuffs are more common in this population. Nevertheless, wrist monitors may introduce systematic errors, as their reliability is affected by the wrist’s position (height in relation to the heart) at the time of BP measurement and the frequent occurrence of artifacts. Although these devices have been improved to take measurements only when the position of the wrist is adequate, more consistent validation and correlation studies are still scarce.^30 - 32^

Recently, new technologies for measuring BP without the use of a cuff have emerged. These devices, called “wearables”, can be attached to bracelets or watches and use different technologies to measure BP, such as photoplethysmography and applanation tonometry. Although there is a lack of studies validating wearables in relation to traditional BP measurement methods, numerous devices are already available on the market. Advantages such as ease of use, nearly continuous BP monitoring, and ease of data sharing and transmission of values (which are obtained almost in real-time), in addition to the fact that some do not require calibration, have attracted a significant number of users. However, current clinical practice lacks specific recommendations for the use of these new technologies and devices in patient monitoring. We will await the conclusion and publication of clinical studies that provide scientific evidence supporting the use of these monitoring devices.^30 - 32^As for finger monitors, they are inaccurate and should be avoided.

### 2.5. BP measurement in older patients33-35

- BP measurement in older patients may be affected by changes associated with the aging process, such as pseudohypertension, auscultatory gap, arrhythmia, and postural hypotension.
- Pseudohypertension occurs when indirect BP measurement is overestimated compared with direct BP measurement, often due to excessive atheromatosis in association or not with medial hypertrophy. In such cases, calcification causes such pronounced stiffening of the arterial wall that cuff inflation cannot collapse the brachial artery. It should be noted that the diagnosis of pseudohypertension is suspected when systolic BP is very high in the absence of target organ impairment or when the patient presents signs of hypotension. The presence of calcium on radiography can further support this diagnosis.
- Osler’s maneuver may help assess pseudohypertension. It involves inflating the arm cuff until the radial pulse disappears. If the artery remains palpable after the procedure, the patient is considered Osler-positive.
- Auscultatory gap refers to the disappearance of Korotkoff sounds between the end of phase I and the beginning of phase II during cuff deflation. This gap may lead to underestimation of systolic BP or overestimation of diastolic BP. To identify and correct for an auscultatory gap, systolic BP must be estimated by the palpatory method.
- Postural, or orthostatic, hypotension is common in older adults with symptoms such as dizziness, blurred vision, scotoma, asthenia, or syncope when standing from a supine position. BP measurement in older patients should be performed with the patient in the sitting, lying, and standing position. Atherosclerotic changes in the carotid sinus region can reduce baroreceptor sensitivity, resulting in greater BP variability and impaired postural reflexes, making these patients more prone to postural hypotension. The use of medications such as diuretics, antidepressants, vasodilators, and beta-blockers can also contribute to postural hypotension. Its prevalence in older adults over 75 years of age has been reported as 34%, and the condition becomes clinically significant when it manifests as postural dizziness, particularly in patients taking hypotensive medication.
- The presence of arrhythmias, such as atrial fibrillation and extrasystoles, can pose challenges when measuring BP with oscillometric devices, unless the devices are equipped with mechanisms to detect atrial fibrillation and other arrhythmias.

### 2.6. BP classification according to in-office measurements

The classification is defined according to in-office BP measurements and the highest systolic or diastolic level. Isolated systolic hypertension is defined as systolic BP ≥ 140 mm Hg and diastolic BP < 90 mm Hg and is classified into stages 1, 2, and 3 ( Chart 8 ).

**Chart 8:** – Classification of blood pressure (BP) according to in-office measurements in patients aged 18 years and older (GR: I – LE: C)

Classification	Systolic BP (mm Hg)	Diastolic BP (mm Hg)
Excellent	< 120 and	< 80
Normal	120-129 and/or	80-84
Prehypertension	130-139 and/or	85-89
Stage 1 hypertension	140-159 and/or	90-99
Stage 2 hypertension	160-179 and/or	100-109
Stage 3 hypertension	≥ 180 and/or	≥ 110

GR: grade of recommendation; LE: level of evidence. Adapted from the Brazilian Guidelines for Hypertension (2020). ^1^

It is important to emphasize that the recognition of hypertension—that is, the final diagnosis—should not be based on a single BP measurement, as BP levels may vary a lot. Therefore, in-office BP values that do not fall within the stage 3 range should be reassessed in subsequent measurements to confirm the diagnosis and determine the stage of hypertension.

For a more accurate assessment, it is recommended to perform multiple in-office BP measurements on different days, with an interval of 1 to 2 minutes between each measurement.^36 - 39^

Nonetheless, in-office (casual) measurements may sometimes be insufficient for characterizing hypertension, as they are subject to numerous biases (systematic errors) and provide fewer measurements. In cases of doubt, alternative measurement methods should be used for diagnosis and follow-up, which were mentioned in this chapter and will be further detailed in the following chapters.

## 3. Out-of-office BP measurement

### 3.1. Self-measured blood pressure (SMBP)

According to the 6th ABPM Guidelines and the 4th HBPM Guidelines, SMBP refers to BP measurements taken by the patient or a family member at home. This method does not follow any pre-established protocols—measurements are taken at the patient’s discretion or at the physician’s request.^18^Unlike ABPM and HBPM, which are performed using automated devices belonging to health institutions whose accuracy and reproducibility has been validated, SMBP is performed with the patient’s own automated device.^17^

The 2020 Brazilian Guidelines for Hypertension made a substantial contribution to the concept of SMBP by recommending a minimum of 7 measurements to be taken over a period of 16-72 hours using quality, validated oscillometric devices. However, studies validating SMBP values in relation to cardiovascular (CV) and renal risk, as well as studies investigating the impact of random BP measurements taken without methodological rigor on medical decision-making, are lacking.^1^

Given the essential role of accurate BP measurements in decision-making, these Guidelines recommend applying the same care and procedures described in Chart 4 to SMBP. Nevertheless, it is important to acknowledge that there is no conclusive evidence supporting the adoption of specific protocols (regarding number of measurements, monitoring schedules, and days) or the establishment of normality thresholds for this method. Therefore, these Guidelines recommend using SMBP only for screening purposes, and confirmatory tests should be requested (HBPM or ABPM) when necessary ( Chart 9 ).

**Chart 9:** – Clinical indications for in-office and out-of-office BP measurement (GR: I – LE: C)

Clinical use	Office	HBPM	ABPM	SMBP	Pharmacy	Public spaces
Screening	+++	+	_	++	++	+
Initial diagnosis	+	++	+++	-	-	-
Treatment titration	+	++	++	+ (?)	-	-
Follow-up	++	+++	+	+ (?)	+ (?)	-
Main indication	Follow-up of treated patients	Follow-up	Initial diagnosis	Screening	Follow-up of treated patients (?)	Opportunistic screening
Values, mm Hg	≥ 140 × 90	≥ 130 × 80	≥ 130 × 80	?	?	?

GR: grade of recommendation; LE: level of evidence; HBPM: home blood pressure monitoring; ABPM: ambulatory blood pressure monitoring; SMBP: self-measured blood pressure. Adapted from Stergiou et al. ^5^

### 3.2. BP measurement in pharmacies

BP measurement in pharmacies is a common practice in Brazil and many other countries. This approach, which is inexpensive for patients, may be useful for hypertension screening and treatment follow-up. However, it lacks established measurement protocols that ensure the validation of devices and measurement methods. Additionally, there is a scarcity of validation studies ( Chart 9 ) and no defined criteria for abnormality thresholds.^1 , 5^

### 3.3. BP measurement in public spaces

Often used in educational campaigns, BP measurement in public spaces has the main advantage of screening for hypertension in the general population for subsequent diagnostic confirmation with in-office BP measurements ( Chart 9 ). Similar to pharmacy measurements, there are no defined criteria for abnormality thresholds.^1 , 4^Therefore, individuals identified as hypertensive who were unaware of their condition, or those who are hypertensive and have not reached their BP goal, should be referred for medical follow-up.

### 3.4. Ambulatory blood pressure monitoring (ABPM)

See Chapters 2 and 3.

### 3.5. Home blood pressure monitoring (HBPM)

See Chapters 2 and 4.

## 4. Central BP measurement and derived parameters

See Chapter 5.

## 5. BP measurement during exercise

In patients with poorly controlled hypertension (CH) and/or an exaggerated BP response to exercise, it is recommended to monitor BP before and during exercise.^1 , 40^

Before initiating exercise, BP measurements should be conducted with the patient in either a seated or standing position, depending on the type of exercise that will be performed. These measurements should be performed using either the auscultatory or oscillometric method, following the previously mentioned recommendations regarding body and arm positioning, prior rest time, cuff dimensions, and measurement technique.^2 , 5^Of note, exercise testing should only be conducted if systolic/diastolic BP values are equal to or lower than 160/105 mm Hg.^1 , 40^

Measuring BP during aerobic exercises may pose challenges in some instances, as accurate readings can only be obtained when the patient can maintain their arm stable at heart level. Ideally, measurements should be taken during metabolic balance, that is, after maintaining intensity for at least 3 minutes. Given the scarcity of automated monitors validated for this purpose, the auscultatory method is recommended.^41^If systolic/diastolic BP values during aerobic exercise are greater than 180/ 105 mm Hg, exercise intensity should be reduced.^1 , 40^

In exercise testing, due to the progressive increase in intensity at different intervals, BP measurement should be performed as recommended in each protocol.^40 , 41^

It is not recommended to measure BP during dynamic resistance training, as neither the auscultatory nor oscillometric method has been validated for this purpose, potentially leading to inaccurate measurements.^41^

## Part 2 – Aspects and concepts common to ABPM and HBPM

## 1. Essential aspects for establishing an ABPM and HBPM service

To create or continue an ABPM and HBPM service, regardless of its management or location, some basic principles must be met, as defined in Chart 10 .

**Chart 10:** – Essential conditions for creating ABPM and/or HBPM services

The BPM-responsible physician is familiar with the method and has technical and scientific knowledge of the examination
The team has a technician qualified to install the BP monitor and provide the appropriate and necessary guidance to the patient
Validated, calibrated automated oscillometric arm BP monitors with memory function; all cuff sizes; charged batteries
Preparation of standardized reports in spreadsheets, software, or online platforms
Appropriate place or site

HBPM: home blood pressure monitoring; ABPM: ambulatory blood pressure monitoring.

ABPM and/or HBPM devices must be automated, digital, and use a validated oscillometric method, with data storage that allows issuing a report. These monitors must have a certificate of validation (see www.stridebp.org), corroborated by the Brazilian National Institute of Metrology, Standardization and Industrial Quality (INMETRO, for the acronym in Portuguese).^27 , 42^For both methods, arm BP monitors are recommended. Exceptionally in HBPM, in patients with an arm circumference greater than 42 cm, the use of a validated wrist BP monitor may be considered, or preference may be given to ABPM using an appropriate cuff size (42 to 50 cm). Furthermore, BP monitors should be checked and calibrated at least every 12 months or whenever decalibration is suspected.

## 2. Abnormality thresholds for in-office and out-of-office BP measurements

BP values considered abnormal are shown in Table 1 . With the advent of out-of-office BP measurement, 8 patterns of BP behavior were defined: normotension (NT), CH, sustained hypertension (SH), sustained uncontrolled hypertension (SUH), WCH, white-coat uncontrolled hypertension (WCUH), MH, and masked uncontrolled hypertension (MUH) (Figures 1 and 2).^1 , 5 , 43^The use of ABPM added a ninth pattern: nocturnal hypertension.

**Table 1:** – Abnormality thresholds for in-office (casual) BP measurements, ABPM (24-hour, daytime, and nighttime), and HBPM to define diagnoses (GR: I – LE: B) **1,18,43**

	SBP (mm Hg)		DBP (mm Hg)
Office	≥ 140	and/or	≥ 90
24-hour ABPM	≥ 130	and/or	≥ 80
Daytime ABPM	≥ 135	and/or	≥ 85
Nighttime ABPM	≥ 120	and/or	≥ 70
HBPM – 5-day ABPM*	≥ 130	and/or	≥ 80

GR: grade of recommendation; LE: level of evidence; BP: blood pressure; HBPM: home blood pressure monitoring; ABPM: ambulatory blood pressure monitoring; SBP: systolic blood pressure; DBP: diastolic blood pressure. *On May 8, 2019, HBPM was introduced into the Brazilian Hierarchical Classification of Medical Procedures (CBHPM for short, in Portuguese) and called 5-day ABPM, code: 2.01.02.16-0. ^44^

### 2.1. Normotension and controlled hypertension

NT is defined as in-office BP < 140/90 mm Hg and average 24-hour ABPM or HBPM < 130/80 mm Hg, without antihypertensive medication use. Individuals with antihypertensive medication use are defined as having CH.^5 , 46^

### 2.2. Sustained hypertension and sustained uncontrolled hypertension

SH is defined as abnormal in-office BP (≥ 140/90 mm Hg) and abnormal average 24-hour ABPM or HBPM (≥ 130/80 mm Hg). Individuals with antihypertensive medication use are defined as having SUH.^5 , 46^

### 2.3. White-coat hypertension and white-coat uncontrolled hypertension

WCH is characterized by abnormal in-office BP (≥ 140/90 mm Hg) and normal 24-hour ABPM (< 130/80 mm Hg) or HBPM (< 130/80 mm Hg) in untreated individuals. When occurring in patients receiving treatment, it is called WCUH.

#### 2.3.1. White-coat effect

The white-coat effect is characterized by elevated BP in the presence of a physician, which can occur within the range of NT or hypertension. The presence of this effect does not change the diagnosis of the patient’s pattern of BP behavior.^47 - 49^In ABPM, a white-coat effect has been empirically considered significant for systolic BP ≥ 20 mm Hg and/or diastolic BP ≥ 10 mm Hg. In HBPM, a white-coat effect, also called alarm reaction, is considered significant for systolic BP ≥ 15 mm Hg and/or diastolic BP ≥ 9 mm Hg.^50^

#### 2.3.2. Investigation

In the absence of pathognomonic data, the characteristics that guide the investigation for the diagnosis of WCH are as follows: office isolated systolic or diastolic hypertension, older people, women, pregnant women, non-smokers, individuals without hypertension-mediated organ damage (HMOD), and particularly, patients with a diagnosis of stage 1 hypertension.^50 - 53^

#### 2.3.3. Prognosis

The risk attributable to patients with WCH has long been debated. Some studies indicate that patients with WCH have an intermediate CV risk between NT and SH, but closer to the risk attributable to normotensive patients.^54 - 56^However, patients with WCUH do not appear to have greater CV risk than those with CH.^57^

#### 2.3.4. Follow-up and treatment

Patients with WCH are at increased risk of developing SH compared with normotensive patients. Therefore, it is recommended that the diagnosis of WCH be confirmed and patients be followed annually, or more frequently in high-risk patients, using ABPM and/or HBPM to detect progression to SH, as they are more likely to become hypertensive.^54^There is no evidence of benefit from drug therapy, and non-drug treatment should be considered for all patients with WCH.^49^

## 2.4. Masked hypertension and masked uncontrolled hypertension

MH is characterized by normal in-office BP (< 140/90 mm Hg) and abnormal 24-hour ABPM (≥ 130/80 mm Hg) or HBPM (≥ 130/80 mm Hg). When occurring in patients with antihypertensive medication use, it is called MUH. It is important to emphasize that, in MH, there is a change in diagnosis from NT in the office to hypertension outside the office.

### 2.4.1. Masking effect

A significant masking effect, or masking reaction, is defined as a difference ≤ −1 mm Hg between the average systolic BP and/or diastolic BP measured in the clinic and average HBPM. However, the presence of this effect does not change the diagnosis of the patient’s pattern of BP behavior.^18 , 58 , 59^

### 2.4.2. Investigation

The characteristics that may suggest the diagnosis of MH and, therefore, deserve investigation are as follows: reports of elevated out-of-office BP measurements and individuals with office prehypertension, HMOD (left ventricular hypertrophy, hypertensive retinopathy, microalbuminuria, altered renal function), or high CV risk, including diabetes and chronic kidney disease (CKD).^52^Individuals with prehypertension have a 3-fold higher risk of developing MH than those without prehypertension.^60^However, the strategy of selecting patients with prehypertension in the office has only modest accuracy in predicting the presence of MH.^60^

### 2.4.3. Prognosis

Compared with normotensive individuals, those with MH have a worse prognosis, which is similar to that of SH. Likewise, MUH has a worse prognosis than CH, tending to be similar to SUH.^53 , 61^

### 2.4.4. Treatment

At first, it can be assumed that the worse prognosis associated with MH may support its treatment, such as in SH.^62^However, to date, there are no results from prospective clinical trials demonstrating an effect of MH treatment on the risk of CV events and mortality.

Given the presence of CV risk similar to that of SH, recommendations on lifestyle changes may be appropriate.^9^Even in the absence of conclusive evidence, if drug treatment is initiated, especially in patients with established HMOD, it is necessary to evaluate the response based on out-of-office BP measurements.^9 , 63^

A flowchart adapted from Huang et al.^63^is suggested in these Guidelines in Figure 3 for evaluation, diagnosis, and management of MH and WCH if ABPM or HBPM are available.

**Figure 3:** – Flowchart for evaluation, diagnosis, and management of masked hypertension and white-coat hypertension. Adapted from Huang et al. ^63^ HBPM: home blood pressure monitoring; ABPM: ambulatory blood pressure monitoring.

## 2.5. Resistant hypertension

Hypertension is defined as resistant to treatment when BP remains above the recommended targets with the use of 3 antihypertensives from different classes, including a renin-angiotensin system blocker (angiotensin-converting enzyme inhibitor or angiotensin receptor blocker), a long-acting calcium channel blocker (at optimal or best-tolerated doses), and a diuretic (preferably a long-acting thiazide diuretic).^64^Resistant hypertension (RH) is a well-defined indication for out-of-office BP measurement.^5 , 18^HBPM and ABPM are mandatory in the diagnosis and monitoring of RH due to the great magnitude of the white-coat effect found in these clinical conditions, becoming essential to rule out pseudo-RH and, therefore, define the 4 patterns of RH behavior: true RH, white-coat RH, masked RH, and controlled RH.^5 , 18 , 64^Although most studies are based on ABPM, there is good agreement between HBPM and ABPM in these patients, with HBPM being the preferred method for monitoring patients with RH, especially those with more elevated in-office BP levels.^65^

## 3. Indications, advantages, and disadvantages of ABPM and HBPM

Indications for ABPM and HBPM are shown in Chart 11 , while the advantages and disadvantages of both methods are shown in Chart 12 .

**Chart 11:** – Indications for ABPM and HBPM (GR: I – LE: C)

Indications	ABPM	HBPM
Suspected WCH – white-coat effect	X	X
Stage 1 hypertension (140-159 and/or 90-99 mm Hg) in the office	X	X
BP > 140/90 mm Hg in the office without HMOD and low CVR	X	X
Isolated systolic or isolated diastolic hypertension in the office	X	X
Suspected MH – masking effect	X	X
BP in the prehypertension range (130-139 and/or 85-89 mm Hg)	X	X
BP < 140/90 mm Hg in the office with HMOD and high CVR	X	X
Suspected white-coat effect	X	X
Elevated in-office BP or suspected pre-eclampsia in pregnant women	X	X
Identifying UH, RH, and excessive decline in BP	X	X
Adjusting antihypertensive medication	X	X
Ensuring adequate BP control	X	X
Evaluating BP control over 24 hours, during sleep and daily activities	X	
Identifying postural, postprandial, and siesta hypotension	X	
Evaluating BP variations in dysautonomia	X	
Evaluating symptoms, especially hypotension	X	
Monitoring long-term treatment efficacy and improving adherence		X

GR: grade of recommendation; LE: level of evidence; WCH: white-coat hypertension; BP: blood pressure; HMOD: hypertension-mediated organ damage; MH: masked hypertension; UH: uncontrolled hypertension; RH: resistant hypertension.

**Chart 12:** – Limitations and advantages of ABPM and HBPM

Limitations	ABPM	HBPM
Difficulty fitting the cuff properly to the patient’s arm	X	X
Clinical conditions associated with movement disorders, such as Parkinson’s disease	X	X
Cardiac arrhythmias such as atrial fibrillation, atrial flutter, and frequent ventricular extrasystoles	X	X
Very high SBP values	X	
Patient’s and/or caregiver’s difficulty performing the measurements		X
**Advantages**	**ABPM**	**HBPM**
Out-of-office BP measurement without the presence of a physician	**X**	**X**
Correlation with better prognosis than in-office BP measurement	**X**	X
Potential reduction in health care costs	**X**	**X**
Current gold standard for BP assessment	**X**	
Evaluation of BP control over 24 hours, during sleep and daily activities	**X**	
Evaluation of morning BP surge	X	
Best method for long-term monitoring		X
Promotion of BP control and adherence to treatment		X
Less expensive than ABPM		**X**
**Disadvantages**	**ABPM**	**HBPM**
Availability is occasionally limited	**X**	**X**
Some patients are reluctant to use and repeat the measurement	**X**	X
It may be uncomfortable, especially while sleeping	X	
Often, nighttime BP is not calculated according to the patient’s sleep schedule	X	
BP measurement only at rest during daytime		X
Guidance and training are required; potential for measurement error: measurement at inappropriate times, excessive number of measurements, induction of anxiety, change of medication by the patient, patient underreports the BP values		X

HBPM: home blood pressure monitoring; ABPM: ambulatory blood pressure monitoring; SBP: systolic blood pressure; BP: blood pressure.

The diagnosis and treatment of hypertension are currently based on in-office and out-of-office BP measurements. Figure 4 presents an algorithm to guide the interpretation of BP patterns in this scenario.

**Figure 4:** – Interpretation of the patterns of in-office and out-of-office blood pressure behavior (GR: IIa – LE: C). GR: grade of recommendation; LE: level of evidence; HBPM: home blood pressure monitoring; ABPM: ambulatory blood pressure monitoring.

## Part 3 – Ambulatory blood pressure monitoring (ABPM)

## 1. Definition

The 24-hour ABPM is a method that allows BP to be measured indirectly and intermittently for 24 hours or more when patients are engaged in their usual activities while awake and during sleep.^18^

ABPM was described more than 50 years ago and is currently performed using fully automated monitors.^66^

## 2. ABPM protocols

It is recommended that the monitor be programmed to record BP at 15- to 20-minute intervals for daytime and 20- to 30-minute intervals for nighttime.^5 , 9 , 18 , 67^The nighttime period can be programmed in advance according to the patient’s usual sleep schedule or defined later as recorded on the activity diary. An appropriately sized arm cuff should be placed always on the nondominant arm, except in special situations or if there is a marked difference in BP between arms (> 10 mm Hg).

### 2.1. ABPM reproducibility

ABPM has good reproducibility as long as it is performed in short time intervals under the same technical and clinical conditions. Average 24-hour, daytime, and nighttime systolic BP and diastolic BP readings present similar values in consecutive measurements performed in short time intervals.^68 , 69^

Nighttime BP dipping also has good reproducibility, with no significant difference between the first and second measurements. The reproducibility of BP pattern between the daytime and nighttime periods is questioned in the literature because of the probability that 30% to 50% of individuals will change status in subsequent recordings, especially in the presence of variables that may interfere with the measurements, such as stress, pharmacological changes, and sleep quality.^18^

## 3. Instructions for patients and health professionals

### 3.1. Activity diary during BP measurements

Patients should be instructed to write brief notes on the activity diary to allow a correlation between activities, symptoms, and medication use during the monitoring period. To ensure the correct completion of the diary, the instructions should be explained to the patient upon removing the BP monitor. An activity diary template is suggested in Figure 5 , and the instructions for completion are shown in Chart 13 .

**Figure 5:** – Diary template to record activities during BP measurements.

**Chart 13:** – Instructions for completion of the activity diary

General instructions
***1.*** All entries on the activity diary must be synchronized with the time on the device’s display.
***2.*** Record the times and activities performed over 24 hours: professional, domestic, school, and physical activities and rest time.
***3.*** *Specific instructions*
**Record:**
***a)*** Name, dose, and time of administration of medications used during the monitoring period.
***b)*** The time of meals, including coffee consumption.
***c)*** If consuming alcohol, cigarettes, and other drugs; record the time and quantity.
***d)*** Time spent in traffic and means of transportation.
***e)*** Occurrence and the time of stressful events.
***f)*** Presence of symptoms, preferably with start and end times, intensity (strong, medium, or weak), type (dizziness, pain, shortness of breath, etc.), and measures taken to resolve them.
***g)*** The time of going to bed and rising, including daytime naps (siesta), and perceived quality of sleep, rating it as good, regular, or poor and comparing the quality of sleep on the recording day with your usual quality of sleep.

### 3.2. General instructions

For successful BP measurements, some essential instructions must be explained to the patient when scheduling ( Chart 14 ) and fitting the BP monitor ( Chart 15 ). The activities of the health professional in charge of fitting and removing the monitor are shown in Chartss 16 and 17, respectively.

**Chart 14:** – Instructions for the patient when scheduling ABPM

On the day of ABPM scheduling, patients must receive the instructions described below:
***1.*** Instruct patients to schedule ABPM preferably on a day representative of their usual activities.
***2.*** Instruct patients to wear a wide-sleeved or sleeveless shirt to avoid limiting arm movement and interfering with cuff fitting; women should avoid wearing dresses.
***3.*** Instruct patients to follow their doctor’s advice about long-term medications.
***4.*** Instruct patients to bring a list of their regular medications with the prescribed dose and time of administration.
***5.*** Instruct patients to take a shower before coming to the clinic, as they will not be allowed to do so during the monitoring period.
***6.*** Instruct patients to bring a belt to facilitate fitting the monitor around their waist.

**Chart 15:** – Instructions for the patient when fitting the ABPM monitor

Patients must be instructed about the aspects described below:
***1.*** Instruct patients not to take a shower or bath during the monitoring period.
***2.*** Instruct patients not to engage in physical training during the monitoring period.
***3.*** Instruct patients how to activate the monitor manually if necessary or in the presence of symptoms.
***4.*** Instruct patients to remain still during measurement with the arm hanging relaxed along the body.
***5.*** Instruct patients how to refit the cuff if it becomes loose during the day and to place the monitor beneath the pillow at night.
***6.*** Instruct patients how to switch off the monitor in case of malfunctioning or an urgent need.
***7.*** Warn patients not to lie down on the arm with the cuff.
***8.*** Instruct patients how to correctly complete the diary, highlighting its importance.
**Recommendations:**
***1.*** The monitor should not be removed, and the cuff should not be switched between arms.
***2.*** The monitor should not be exposed to water, ice, excessive dust, or heat.
***3.*** Patients should follow their usual daily activities during the monitoring period.
***4.*** Patients should contact the clinic in case of emergency.

## 4. Interpretation of results

### 4.1. Criteria for interpreting BP measurements

A minimum of 16 valid daytime and 8 nighttime BP measurements are required for a valid ABPM session.^18^Ideally, the monitoring period should not have time intervals between measurements longer than 2 hours or total duration less than 22 hours. Depending on the purpose of ABPM and in special situations, based on clinical judgment, fewer measurements than recommended may be accepted or the period with an appropriate number of measurements may be analyzed separately, whether daytime or nighttime.

### 4.2. Abnormality thresholds

Despite the continuous relationship between BP measured by ABPM and CV risk, in clinical practice, it is necessary to establish abnormality thresholds. Hypertension diagnostic thresholds for BP measured by ABPM are currently defined by few cohort studies and international guidelines (see Table 2 in Part 2).^54 , 56 , 70 , 71^

**Table 2:** – Classification of blood pressure patterns in transition periods between daytime and nighttime (GR: I – LE: B)

Blood pressure (BP) pattern	BP dipping (%)
Present dipping	≥ 10 ≤ 20
Absent dipping or BP surge	≤ 0
Attenuated dipping	> 0 < 10
Blunted dipping	>20

GR: grade of recommendation; LE: level of evidence.

### 4.3. BP patterns in transition periods between daytime and nighttime

Among the BP measurement methods, ABPM has the unique characteristic of recording BP during sleep. The sleep-wake pattern is characterized by evaluating the percentage difference between daytime and nighttime average BP values ( Table 2 ).^72^There is no evidence that these patterns of BP dipping have any therapeutic value, thus being considered only a risk marker.

Patients considered to be at highest risk are those whose nighttime average BP does not fall by 10% to 20% of daytime values.^73^

### 4.4. Morning BP surge

Morning BP surge reflects an increase in BP in early morning hours as a result of sympathetic activity. Several methods to determine morning BP surge have been proposed in different studies.^74 - 77^However, difficulty in standardizing the calculation of this parameter and its poor reproducibility indicate that it should not be used in clinical practice.^72^

### 4.5. BP load, areas under the curves, BP variability, and heart rate

#### 4.5.1. BP load

This index is defined as the percentage of abnormal readings in a given measurement period (daytime, nighttime, or 24-hour). Figure 6 exemplifies the calculation of BP load. Because its calculation only indicates that there were readings with elevated BP values (without quantifying them), there may be patients with the same BP load but completely different average BP values. The weak correlation of BP load with clinical outcomes makes its use irrelevant to clinical practice.^78^

**Figure 6:** – Example of how to calculate blood pressure (BP) load. SBP: systolic blood pressure; DBP: diastolic blood pressure; BP: blood pressure.

#### 4.5.2. Areas under the curves

This index is defined as the area under the recorded BP curve that exceeds the BP normality threshold in a given measurement period.^79^

This parameter can be considered to have greater clinical relevance than BP load alone because it takes into account, in addition to the number of readings exceeding the normality threshold value, the level of the recorded values.^79^This two-dimensional assessment provides a more accurate estimate of the impact of 24-hour BP. However, this planimetric measurement is not available in commercial software packages used for generating ABPM reports. BP load and average 24-hour, daytime, and nighttime BP values have shown to be strongly correlated with the areas under the BP curves. Likewise, the areas under the curves have shown a good correlation with left ventricular hypertrophy. However, due to the lack of normality parameters and evidence of a correlation between this index and clinical outcomes, there is currently no indication for its use in clinical practice.

#### 4.5.3. BP variability

This index is defined as systolic and diastolic BP variations over the 24-hour recording period. It can be assessed individually over 24 hours, daytime, or nighttime. In clinical reports, BP variability is expressed as the standard deviation of BP values, although it can also be obtained by the coefficient of variance and “average real variability.”^80^

Based on studies of continuous BP measurement, evidence has accumulated correlating increased BP variability with undesirable CV, renal, and neurological outcomes and with increased risk of WCH and MH.^63^However, because of the lack of reference values for normality in BP variability and the lack of data supporting that the treatment acting on variability would bring any clinical advantage, its application is impaired.

#### 4.5.4 Heart rate

Heart rate (HR) is estimated from the value of heartbeat frequency derived from pulse rate acquired over the 24-hour BP recording period.^81^This parameter is little studied in terms of clinical significance based on ABPM recordings, since conventional devices have poor sensitivity to estimate it.

Because HR obtained with ABPM is a highly error-prone indicator and lacks reference values for normality, it has no clinical application.

## 5. Interpretation of ABPM and technical report

### 5.1. Technical report

The ABPM report must contain the items listed in Chart 18 .^82^The different patterns of BP behavior, such as NT, hypertension, WCH, or MH, should not be established, as they are clinical diagnoses.

**Chart 18:** – I Required items in the ABPM report (GR: I – LE: C)

Date with start and end time of the ABPM session
Number and percentage of BP measurements recorded and valid BP readings
Average 24-hour, daytime, and nighttime systolic BP values
Average 24-hour, daytime, and nighttime diastolic BP values
BP pattern between daytime and nighttime
Hypotensive episodes
Correlation between activities, symptoms, and medication use
Conclusion

GR: grade of recommendation; LE: level of evidence; ABPM: ambulatory blood pressure monitoring.

### 5.2. Systolic and diastolic BP patterns over 24 hours, daytime, and nighttime

The report should include the average 24-hour, daytime, and nighttime systolic and diastolic BP values. In both treated and untreated patients with hypertension, there is a positive correlation of 24-hour, daytime, and nighttime systolic BP and/or diastolic BP values with CV morbidity and mortality as well as HMOD.^71 , 83 - 85^

### 5.3. Systolic and diastolic BP patterns in transition periods between daytime and nighttime

The classification of BP patterns in transition periods between daytime and nighttime is shown in Table 2 . The report should describe the classification of the morning surge in systolic BP and diastolic BP jointly when the classifications are the same, and separately when the classifications are different.^86^

It is essential to describe the time of going to bed and rising of each individual to define the daytime and nighttime periods. This information should be clearly recorded in the activity diary. Self-reported quality of sleep during the monitoring period should also be considered when interpreting the results.

It is worth noting that reverse dipping or rising, non-dipping, or BP surge may be related to certain conditions, such as sleep disturbances caused by the measurements, inadequate BP control in treated patients, some forms of secondary hypertension, individuals with sleep apnea, dysautonomia, and use of some medications such as cyclosporine.

### 5.4. BP spikes and hypotension

BP spikes are defined as 2 or 3 progressive elevations in BP measurements, all of which are well above the average BP values observed before and after the event. However, in most cases, isolated elevated BP readings correspond to artifacts and should not be characterized as BP spikes. Due to the lack of prospective studies evaluating prognosis after BP spikes, their significance is unclear and, therefore, they should not be described in the report.

Hypotension is characterized by episodes of a marked fall in BP below the average BP values observed before and after the event, as long as they are accompanied by symptoms ( Figure 7 ). BP falls that are not accompanied by symptoms should not be described as hypotension.

**Figure 7:** – Episode of a fall in blood pressure on ABPM.

Symptomatic episodes of a BP fall may occur in the following situations: medication use, syncope, lipothymia, postural hypotension, and autonomic neuropathy. Isolated BP readings not accompanied by symptoms, even with a marked fall in BP, may also result from technical artifacts.

### 5.5. Correlation between activities, medication use, and symptoms

Correct completion of the activity diary by the patient is crucial to correlate changes in BP with medications used, activities performed, and symptoms experienced during the ABPM session. Medication doses, the time of administration, and the time of main activities (going to bed, rising, breakfast, lunch, and dinner) should be recorded.

Symptoms should be recorded along with the time they have occurred and their intensity. The report should inform whether the described symptom caused any variation in BP. It should be recorded in the diary whether the medication was taken on the day of ABPM, with a list of the antihypertensive medications used, the doses and time of drug intake.

### 5.6. Conclusion

The conclusion should include the following information (GR: I – LE: C):

- When the patient reports good or regular sleep quality:
  1. The pattern of systolic BP and/or diastolic BP was normal or abnormal over the 24-hour recording period.
  2. Nighttime systolic BP and/or diastolic BP dipping was present, absent, blunted, or attenuated.
  3. Systolic BP and/or diastolic BP was controlled or uncontrolled during the daytime and nighttime periods if antihypertensive medication use was reported.
- When the patient reports poor sleep quality, nighttime BP dipping should not be analyzed, and the pattern and control of BP should only be described for the daytime period:
  1. The pattern of systolic BP and/or diastolic BP was normal or abnormal during the **daytime** period.
  2. Systolic BP and/or diastolic BP was controlled or uncontrolled during the **daytime** period.

Example of how to write a conclusion when the patient reports good or regular sleep quality:

1. Abnormal systolic blood pressure pattern and normal diastolic blood pressure pattern based on the 24-hour average blood pressure value.
2. Absent dipping of nighttime systolic blood pressure and blunted dipping of nighttime diastolic blood pressure.
3. The reported medication controlled diastolic blood pressure but not systolic blood pressure during the daytime and nighttime periods.

Example of how to write a conclusion when the patient reports poor sleep quality:

1. Abnormal systolic and diastolic blood pressure patterns based on the daytime average blood pressure value.
2. The reported medication did not control systolic or diastolic blood pressure during the daytime period.

Example of how to describe night-to-day BP variations when the patient reports poor sleep quality.

1. Night-to-day blood pressure variations.

Patient reports poor sleep quality. Therefore, the analysis of nighttime blood pressure pattern cannot be performed.

The following note should be placed at the end of the report:

ABPM, as well as other complementary medical examinations, should be assessed at the discretion of the attending physician.

Finally, it is important to highlight that the report template suggested here is only a guide to what can be done. Physicians can create their own report template as long as it provides essential information for the physician ordering the ABPM.

Another important factor is that the report should be limited to the information provided by ABPM, without containing deductions or clinical conclusions that are not supported by the data from the ABPM session.

## 6. Clinical applications of ABPM

### 6.1. Assessment of prognosis in patients with hypertension

The different BP components obtained with ABPM provide relevant information for the assessment of CV risk and prognosis in patients with hypertension.

Average 24-hour, daytime, and nighttime BP values correlate more strongly with HMOD, morbidity, and mortality than in-office (casual) BP measurements.^87^

Nighttime BP pattern, a parameter only obtainable by ABPM among all indirect BP measurement methods, also determines CV risk, with an independent association between elevated nighttime systolic BP and CV mortality.^88^Patients with attenuated nighttime systolic BP dipping have shown a higher incidence of lacunar stroke than patients with normal BP dipping.^89^

Attenuated nighttime BP dipping is associated with an increased risk of CV events also in individuals with CKD, in older adults, and in patients with CAD.^89 - 91^

An independent association between BP dipping and CV events was also demonstrated in a cohort of patients with RH. A BP reduction of less than 10%, or nocturnal BP elevation, was associated with a composite endpoint of CV events and all-cause mortality after a mean follow-up of 4.8 years.^92^

### 6.2. Assessment of antihypertensive treatment efficacy

Guidelines have placed greater emphasis on the role of ABPM in diagnosis and its considerable prognostic power than on the evaluation of treatment efficacy. Some studies have demonstrated a discrepancy in response to treatment between ABPM and in-office BP.^93 , 94^BP control was demonstrated in only 12% of cases by in-office BP assessment, against 33% with ABPM. Furthermore, 38% of patients had their prescription changed by ABPM, 32% had to add another medication, and 14% of patients with newly diagnosed hypertension in the office were maintained without medication after ABPM.^93^Longitudinal studies using ABPM, specifically designed to evaluate treatment efficacy, are needed before generalizing the method’s indications to all patients with hypertension.

## 7. ABPM in special situations

### 7.1. Children and adolescents

Substantial data exist that link elevated BP levels measured in childhood and adolescence to current and future HMOD.^95^

Normative ambulatory definitions for ABPM values in the pediatric population are derived from studies of healthy populations, and recommendations for the use of ABPM in this population are based on expert opinions rather than on evidence arising from well-designed studies for this purpose.^96^

ABPM is mandatory to confirm the diagnosis of hypertension in children and adolescents with in-office BP measurements in the elevated BP category for 1 year or more or with stage 1 hypertension over 3 consecutive clinic visits. ABPM is also mandatory to assess hypertension severity and determine if abnormal circadian BP patterns are present in children and adolescents with high-risk conditions, such as CKD, type 1 and 2 diabetes, preoperative and postoperative periods of aortic coarctation, solid-organ transplant, obstructive sleep apnea syndrome (OSAS), obesity, suspected MH or WCH, and genetic syndromes associated with hypertension (Williams syndrome, Turner syndrome, and neurofibromatosis).^97^

The recently published new normative data for pediatric ABPM presents an updated classification scheme for ABPM measurements, which, in addition to favoring the transition of care from adolescents to young adults, eliminates the use of BP load ( Table 3 ).^98^

**Table 3:** – Classification for in-office BP by ABPM in pediatric patients (GR: IIa – LE: B)

	SBP/DBP office		SBP/DBP ABPM	
	< 13 years of age	≥ 13 years of age	< 13 years of age	≥ 13 years of age
Normal BP	< p95	< 130/80	< p95 OR adolescent cutoff points*	< 125/75 mm Hg 24 hours AND < 130/80 mm Hg daytime AND < 110/65 mm Hg nighttime
WCH	≥ p95	≥ 130/80		
MH	< p95	< 130/80	≥ p95 OR adolescent cutoff points*	≥ 125/75 mm Hg 24 hours OR ≥ 130/80 mm Hg daytime OR ≥ 110/65 mm Hg nighttime
Ambulatory hypertension	≥ p95	≥ 130/80		

GR: grade of recommendation; LE: level of evidence; BP: blood pressure; SBP: systolic blood pressure; DBP: diastolic blood pressure; WCH: white-coat hypertension; MH: masked hypertension; p95: 95th percentile. *In children < 13 years of age, select the lower of either the 24-hour 95th percentile or the respective adult cutoff point in the ≥ 13 years of age column.

### 7.2. Pregnant women

The role of ABPM in pregnancy has not been clearly defined. ABPM is particularly useful in the first half of pregnancy.^99^WCH or MH can occur in up to one-third of pregnant women. Their identification is essential to avoid unnecessary and potentially harmful treatment to the fetus.^99^

WCH has a more favorable prognosis than gestational hypertension, persisting in 50% of cases throughout pregnancy without being associated with complications. However, 40% of pregnant women develop gestational hypertension, and 8% of pregnancies progress to pre-eclampsia.^100^On the other hand, a study showed that 22% of 158 pregnant women with hypertension confirmed by ABPM developed pre-eclampsia.^100^In this study, sleep hypertension occurred in 60% of cases, being more commonly associated with the risk of pre-eclampsia and fetal complications. A suspected diagnosis of MH is more challenging, and it should be investigated in the presence of HMOD in pregnant women.

These Guidelines suggest that:

- ABPM is indicated to evaluate suspected WCH and MH during pregnancy.
- The diagnostic threshold for gestational hypertension should be identical to that used for the general population (ABPM ≥ 130/80 mm Hg).^101^

### 7.3. Older people

ABPM can provide valuable clinical information in older patients, such as in cases of suspected postural, postprandial, drug-induced, and episodic hypotension, as well as in the assessment of dysautonomia and syncope. Some limitations should be highlighted in older patients: a) the same ABPM normality thresholds adopted for non-older adults are accepted for older adults; and b) reduced nighttime BP dipping, increased pulse rate, and early morning BP surge, common in older people, are associated with increased CV risk.^84 , 102 , 103^

Patients with a blunted nocturnal fall in BP, with a > 20% fall in systolic BP, had a higher incidence of ischemic stroke, whereas a nocturnal rise in systolic BP was associated with a higher risk of hemorrhagic stroke.^104^

### 7.4. Diabetes

The absence of nighttime BP dipping, an increase in nighttime BP relative to daytime BP, sleep hypertension, MH, and BP variability are highly prevalent in patients with type 2 diabetes, with or without a known history of hypertension.^105^In patients with diabetes, ABPM can contribute to the assessment of hypotension secondary to CV autonomic neuropathy, often related to symptoms such as syncope, dizziness, and sweating, thus assisting in the differential diagnosis with hypoglycemia.^106^Nighttime systolic hypertension stands out as a predictor of HMOD in patients with type 2 diabetes.^105^However, the absence of nighttime BP dipping has been associated with cardiovascular disease (CVD), neuropathy, and retinopathy, whereas morning BP surge has been associated with neuropathy.^107^

### 7.5. Chronic kidney disease

At any stage of CKD, ABPM can identify changes in the sleep-wake pattern and detect episodes of arterial hypotension, MH, and WCH.^51^Furthermore, in individuals with CKD, BP values obtained with ABPM are independently correlated with left ventricular mass, glomerular filtration rate, proteinuria, and other HMOD.^108 , 109^In hemodialysis patients, 24-hour ABPM may not encompass BP measurements throughout the dialysis cycle. Therefore, performing a 44-hour ABPM, fitting the device after a midweek dialysis session (between the second and third sessions of the week) and removing it immediately before the next session, provides a more adequate assessment.^90^In practical terms, if the software used does not include the 44-hour protocol, we suggest performing two 22-hour ABPM sessions on two consecutive days. These Guidelines do not recommend the use of 24-hour ABPM in hemodialysis patients. In this population, the cuff should not be placed on the arm with arteriovenous fistula. Patients treated with continuous or automated ambulatory peritoneal dialysis have also shown changes in the nighttime BP pattern.^110^

### 7.6 Obstructive sleep apnea syndrome

In the pathophysiology of OSAS, episodes of micro-arousals and intermittent hypoxia trigger, among other events, the activation of the sympathetic nervous system, leading to an increase in BP, especially during sleep.^111^

These pathophysiological changes determine significant changes in the BP pattern measured by ABPM, significantly increasing the rate of patients with attenuated or absent nighttime BP dipping.^112^

A meta-analysis showed a prevalence of 59% of attenuated nighttime BP dipping measured by ABPM in patients with OSAS, and those with at least moderate OSAS (apnea/hypopnea index > 15 events/hour) were at least 1.67 times more likely to have attenuated dipping. This observation may explain, at least in part, the increased CV risk in patients with OSAS.^113^

A cross-sectional study including 153 patients showed that patients with absent nighttime systolic BP dipping on ABPM were 3.5 times more likely to have moderate OSAS when undergoing polysomnography, thus showing that ABPM data can screen patients who are more likely to be diagnosed with OSAS when undergoing polysomnography.^114^

### 7.7. Heart failure

ABPM may be indicated to improve the treatment of patients with heart failure (HF) whose symptoms are related to changes in BP, such as in cases of paroxysmal nocturnal dyspnea or HF with preserved ejection fraction (EF). Likewise, ABPM can be useful to guide the treatment of patients with symptoms caused by hypotension, since many of those with advanced HF have fatigue, symptoms of coronary insufficiency, or cerebral manifestations. ABPM can also be used to evaluate patients with HF who will undergo physical training programs.^115^

The absence of nighttime BP dipping is more common in patients with HF.^116^Changes in the sleep-wake pattern have been associated with the severity of systolic dysfunction.^117^

More recently, a study evaluating all-cause and CV mortality in patients with HF with reduced EF, HF with mildly reduced EF, and HF with preserved EF showed that elevated systolic BP was associated with an increased risk among patients with HF with preserved EF, but not in patients with HF with reduced or mildly reduced EF.^118^In patients with HF with reduced EF, lower systolic/diastolic BP and the presence of nighttime BP dipping were predictors of high mortality compared with those with higher systolic/diastolic BP and nighttime non-dipping.^119^

### 7.8. Physical activity

Engaging in physical activity during ABPM may lead to inaccurate readings or missing measurements.^120^After a physical exercise session, BP decreases for several hours, which is more pronounced in patients with hypertension and can change the patient’s average ambulatory BP values.^121^Therefore, physical activity should be avoided during ABPM, and also on the day before the ABPM session in patients who do not exercise regularly.

## 8. Cost-effectiveness

ABPM is the strategy of choice for diagnosis and initiation of treatment in most adults in primary care. The correct diagnosis of WCH and MH reduces the total cost of treating hypertension and future CV events.^67^The National Institute for Health and Care Excellence (NICE) conducted a rigorous cost-effectiveness analysis that demonstrated that ABPM would not only be a more effective means of diagnosing hypertension, but it would also provide a more cost-effective approach than in-office BP measurement or HBPM across all age and sex subgroups, leading to an improvement in quality health outcomes and cost savings when long-term costs were taken into account. The key cost-saving factor in this analysis was that the cost of antihypertensive treatment would be avoided due to improved specificity in making a diagnosis with ABPM. The model suggested that antihypertensive therapy would be needed in approximately 25% fewer patients than if the diagnosis were made based on in-office BP alone. The drug cost savings outweighed the cost increases associated with the use of ABPM. The analyses were based on the United Kingdom model of health care delivery and may not be valid for use in other countries.^122^Using ABPM reduces the costs with drugs and medical appointments compared with using in-office BP measurements.^123^The benefits of ABPM are unquestionable, especially in primary care. ABPM has been implemented in the Brazilian public health system (SUS for short, in Portuguese) and is already fully incorporated into the private health insurance system. In Latin America, many scientific societies of hypertension and/or cardiology have given special attention in their guidelines to the use of ABPM for the diagnosis and control of hypertension in order to encourage health authorities to regulate access and allow more patients to have access to its benefits.

## 9. Perspectives

Since the introduction of 24-hour ABPM, in 1964, there has been great progress in the devices used for BP measurement. These technological advances have improved comfort for patients during monitoring and enabled cost savings.

Further advances are expected in the near future, as well as the development of devices capable of recording peripheral BP measurements, beat-to-beat monitoring, using a chip that can detect mechanical phenomena arising from hemodynamics and transform them into systolic and diastolic BP values.

Likewise, 24-hour recording of central hemodynamic parameters, including aortic BP, pulse wave velocity (PWV), and augmentation index, has become a reality.

Over time, we will be able to understand the broader applicability of the parameters obtained and, certainly, at that point, ABPM will have become widely accessible to the public in daily clinical practice.

Future applications and possibilities for using ABPM include:

- ABPM monitors with an attached actigraph.
- Adjustable cuffs.
- Assessment of parameters other than systolic BP and diastolic BP, such as HR, pulse rate, PWV and pulse waveform, BP variability over 24 hours or in subperiods, and morning BP surge.
- Reference values for 24-hour ABPM derived from studies of different populations worldwide.
- Prospective studies to evaluate the prognosis and effectiveness of antihypertensive treatment in populations followed by ABPM.
- Development of low-cost devices for noninvasive beat-to-beat BP monitoring.

## Part 4 – Home blood pressure monitoring (HBPM)

## 1. Introduction

HBPM assists in the diagnosis and monitoring of hypertension by taking multiple out-of-office BP readings over days in the individual’s usual environment. It should be performed by a person trained in oscillometric BP measurement, preferably the patient himself/herself. In cases where self-measurement is not possible, another trained individual may assist. A fundamental aspect of HBPM is adherence to a previously validated, established, and standardized protocol.^1 , 5^

## 2. Instructions for patients

The effectiveness of HBPM fundamentally depends on the instructions provided to the patient, which should address factors that can influence BP levels or produce artifacts, such as the environment, patient preparation, and positioning.^1 , 5^Home measurements should follow the procedural steps as in-office measurements and are also subject to transient variations ( Chart 19 ).

**Chart 19:** –General HBPM instructions for patients

Conditions
Quiet room with comfortable temperature
No smoking, caffeine, food, or exercise for 30 minutes before measurement
Remain seated and relaxed for at least 3 minutes (ideally 5 minutes), with an empty bladder
No talking during or between measurements
**Posture**
Sitting with back supported by chair
Legs uncrossed and feet flat on floor
Bare arm resting on table, palm facing up, mid-arm at heart level
Wrap the cuff around the arm according to the device instructions
Use the same arm to take all measurements—the arm with the highest in-office reading should be chosen (measurements should ideally be performed simultaneously)
Educate the patient on BP variability: “BP varies with each heartbeat”
Explain that, in some people, out-of-office BP may be lower, whereas others might present higher BP measurements at home
Instruct the patient to take measurements on the days and times recommended by the protocol, without changing daily routine
Emphasize that is not permitted to take BP readings of other people
Explain that the therapeutic regimen should not be altered as a result of BP measurements observed during HBPM—occasional high or low values are not a cause of concern

HBPM: home blood pressure monitoring; BP: blood pressure.

## 3. HBPM protocol

The HBPM protocol aims to capture a patient’s usual level of BP, assisting the physician in clinical decision-making.^124^HBPM reproducibility is directly associated with the number of measurements that are averaged.^125^Based on several studies and published evidence, our recommendations are as follows (GR: I – LE: C):^1^

- **Number of measurements:** ideally, the patient should take 24 to 36 valid readings.
- **Monitoring period:** 4 to 6 days.
- **Day 0 or fitting day:** on this day, BP measurements are initially taken at the doctor’s office (ideally 3), with the average of the last 2 readings used to account for a masking or alarm reaction, then at home in the evening to correct for a potential white-coat effect. Home and in-office BP measurements taken on day 0 must be discarded, and the average should be calculated using measurements collected from day 1 onwards.
- **Days 1-6:** home readings are taken for 4 to 6 days. The patient should take 3 readings in the morning and another 3 in the evening/night, always after 5 minutes of rest, before meals, with an empty bladder, and before intake of hypertensive medication (if applicable). If the patient has recently eaten, measurements should only be taken 2 hours after the meal.
- **Discarded measurements:** values that fall outside the following ranges should be excluded, unless there is clinical justification for their inclusion: diastolic BP 40-140 mm Hg, systolic BP 70-250 mm Hg, and pulse pressure 20-100 mm Hg, as well as systolic BP values lower than the previous or next diastolic BP reading and/or diastolic BP values higher than the previous or next systolic BP reading.

Keeping a record of BP measurements on a BP diary is extremely valuable. In addition, the diary should also include information on current medication. This helps the patient to correctly adhere to the protocol and facilitates the transcription of BP values (for devices lacking data transmission) to elaborate the technical report. A BP diary template is shown in Figure 8 .

**Figure 8:** – Diary for home blood pressure monitoring (HBPM).

## 4. Abnormality thresholds

In the 2020 Brazilian Guidelines for Hypertension,^1^average HBPM measurements ≥ 130 mm Hg for systolic BP and/or ≥ 80 mm Hg for diastolic BP were considered abnormal.^1 , 50 , 126 - 129^These values differ from the ones recommended by the 7^th^Brazilian Guidelines for Hypertension^130^(published in 2016) and by the 4th HBPM Guidelines (published in 2018),^18^in which average HBPM ≥ 135 mm Hg for systolic BP and/or ≥ 85 mm Hg for diastolic BP was considered abnormal. The estimated prevalence of hypertension in the Brazilian population based on these two thresholds is shown in Table 4 , while the estimated correspondence between HBPM and in-office BP values is presented in Table 5 .

**Table 4:** – Prevalence of hypertension and hypertension phenotypes based on the abnormality thresholds recommended by the 4th HBPM Guidelines and the 2020 Brazilian Guidelines for Hypertension **1 , 18**

Hypertension	HBPM ≥ 135/85 mm Hg*	HBPM ≥ 130/80 mm Hg**
Diagnosis	37%	57%
Normotension	47%	36%
WCH	16%	7%
MH	10%	22%
SH	27%	35%
Control	40%	58%
Controlled hypertension	45%	34%
White-coat uncontrolled hypertension	15%	8%
Masked uncontrolled hypertension	11%	22%
Uncontrolled SH	29%	36%

HBPM: home blood pressure monitoring; WHC: white-coat hypertension; MH: masked hypertension; SH: systolic hypertension.

**Table 5:** – Estimated correspondence between HBPM and in-office BP values (GR: IIa – LE: B) ^**128**^

In-office Systolic/diastolic BP (mm Hg)	HBPM Systolic/diastolic BP (mm Hg)
< 120/< 80	< 120/< 75
120-129/80-84	120-124/< 75
130-139/85-89	125-129/75-79
140-159/90-99	130-139/80-89
160-179/100-109	140-149/90-95
≥ 180/≥ 110	≥ 150/≥ 95

GR: grade of recommendation; LE: level of evidence; HBPM: home blood pressure monitoring. *The classification is defined according to in-office BP measurements or HBPM and the highest systolic or diastolic value (depending on the technique of choice).

The normality thresholds are defined based on the average BP values obtained in the entire HBPM period. however, calculating separate averages for the morning and evening periods may be useful for establishing more individual strategies for drug therapy.

## 5. Technical report and interpretation of results

The HBPM report should include the following information (GR: I – LE: C):

- Reason for requesting HBPM: state the indication for HBPM.
- Protocol description: state the number of valid monitoring days, times, and number of measurements per day.
- Session quality: the HBPM session will only be considered valid when at least 14 readings have been taken in the 4-day protocol, 15 readings in the 5-day protocol, and 18 readings in the 6-day protocol, every day, in the morning and in the evening.
- BP averages: report the overall, daily, and morning and evening averages, especially for patients on antihypertensive medication. The averages should be calculated from valid readings obtained over at least 4 days (ideally 6). Readings taken on day 0 should be discarded, as they were taken in the office and the device was handed to the patient.
- White-coat and masking effects: calculate by subtracting HBPM from in-office BP.
- Abnormality thresholds: averages ≥ 130 mm Hg and/or ≥ 80 mm Hg should be considered abnormal.
- Conclusion:

- BP behavior during HBPM was normal or abnormal (according to the overall HBPM average).
- A masking and/or alarm reaction was observed, as indicated by the difference between in-office BP and HBPM (asses systolic and diastolic BP separately).
- The use of hypertensive medication was reported (or not).

The following note should be placed at the end of the report:

HBPM, as well as other complementary medical examinations, should be assessed at the discretion of the attending physician.

## 6. Applications of HBPM

### 6.1. Determination of in-office and out-of-office BP behavior

BP varies continuously in response to internal and external factors, which may lead to misdiagnosis of hypertension, resistance to treatment, or NT.^131^HBPM is superior to in-office BP measurement in the evaluation of BP behavior, particularly as it allows the diagnosis of WCH and MH.^132 - 134^Furthermore, HBPM can evaluate day-to-day and seasonal BP variability, in addition to separately determining BP behavior in the morning and in the evening/night. However, the incremental value of these measurements compared with the average BP value in predicting CV and renal risk remains uncertain.^135 - 137^

### 6.2. Assessment of prognosis

Several studies have consistently demonstrated that HBPM is strongly associated with HMOD prediction, particularly left ventricular hypertrophy, and has a greater capacity to predict CV and renal events than in-office BP.^138 - 140^Results from a meta-analysis including approximately 18,000 patients showed that home BP was superior to in-office BP in predicting CV mortality: the hazard ratio for home BP was 1.29 (95% CI = 1.02-1 .64) per 10 mm Hg increase in systolic BP compared with 1.15 (95% CI = 0.91-1.46) for in-office BP.^138^

HBPM also proved to be similarly reliable in predicting HMOD as ABPM.^139^Moreover, a meta-analysis of studies that exclusively performed HBPM or ABPM showed that MH and MUH detected by these methods had similar predictive value for CV events and mortality, irrespective of the diagnostic method.^141^Taken together, these findings suggest that ABPM and HBPM may be equally effective in predicting CV risk. However, studies directly comparing the capacity of HBPM and ABPM to predict CV events in the same population are scarce, precluding more definitive conclusions on this topic.^142^A recent analysis of the PAMELA study showed that HBPM and ABPM were superior to in-office BP measurement in predicting CV risk and even suggested that HBPM could be more accurate than ABPM in predicting CV and total mortality.^143^Furthermore, the day-to-day variability of home BP has been shown to have predictive value for cerebrovascular, renal, and CV diseases.^140^

### 6.3. Assessment of antihypertensive treatment

The main contributions of HBPM to the treatment of hypertension are the characterization of hypertension phenotypes, particularly WCUH and MUH, and the confirmation of controlled, uncontrolled, and resistant hypertension.^1 , 5 , 18 , 144^Identifying these phenotypes allows a more personalized approach, with individualized therapeutic adjustments.^5 , 18 , 144^

Because of its wide accessibility, affordability, and user-friendliness, HBPM is well-tolerated by both the patient and physician. Therefore, HBPM is the preferred method for monitoring treated hypertensive patients, allowing drug titration to achieve BP control and long-term monitoring.^5 , 144 - 146^

Regarding BP goals to be achieved in HBPM with treatment, systolic BP values < 130 mm Hg and diastolic BP values < 80 mm Hg are recommended.^145 , 147^Studies have shown that performing HBPM can increase patient engagement and adherence to long-term treatment, and it can be used for BP telemonitoring. The combination of these factors can help improve BP control, particularly when combined with patient education and counseling.^148 - 150^

### 6.4. In special populations and situations

#### 6.4.1. Children and adolescents

The practice of out-of-office BP measurement is also encouraged in children and adolescents, as they may also present with WCH and MH. Measurements should be taken using appropriately sized cuffs and devices validated for use in this population. Studies investigating reference values for HBPM in children and adolescents are scarce. In adolescents, these Guidelines recommend using values ≥ 95th percentile of the normality thresholds obtained from a Brazilian population to diagnose hypertension with HBMP.^151 , 152^

#### 6.4.2. Pregnant women

In pregnant women, it is recommended to measure BP during each prenatal care visit. However, even regular prenatal checkups may not be sufficient to identify conditions such as pre-eclampsia or WCH, the latter which is common at the end of pregnancy.^144 , 153^The use of HBPM during pregnancy has some advantages, as it is well-accepted by women and facilitates treatment monitoring, thereby reducing the number of medical appointments. When performing HBPM during pregnancy, the patient should be in a sitting position^144 , 153 , 154^and use devices that were specifically validated for this population.

During pregnancy, we recommend the use of ABPM and HBPM to assess WCH and MH, in order to avoid unnecessary and potentially harmful treatment to the fetus.^155^ABPM should be used before 20 weeks of pregnancy and HBPM after 20 weeks.^153^

#### 6.4.3. Older patients

HBPM is an extremely important tool in the initial assessment and periodic therapeutic monitoring of older patients, contributing to a better prognosis.^156 , 157^This population usually has a high CV risk, greater BP variability, and less tolerance to inadequate treatment, such as antihypertensive use for WCH.^158^

There is no consensus on whether age is a risk factor for a higher prevalence of WCH and MH.^50 , 159^HBPM is feasible with minimal training in older patients, but extra care should be taken when initially training those over 80 years old, with low educational level, cognitive decline, or physical restrictions requiring assistance from others.^160^An alternative for achieving better BP control among older patients with hypertension is the use of telemonitoring.^161^

#### 6.4.4 Diabetes

Diabetes is a disease with different pathophysiological mechanisms that, in general, doubles the risk of CV and renal outcomes, including stroke, CAD, CKD, and CV death.^162 , 163^Patients with diabetes, particularly due to visceral fat and insulin resistance, are more prone to having MH than those without diabetes.^43^In a small observational study involving 170 patients with type 2 diabetes, HBPM was superior to in-office BP in identifying microvascular complications.^164^This finding suggests that HBPM has great potential in the clinical management of patients, emphasizing the need for clinical trials to further investigate its clinical relevance in this population.

#### 6.4.5. Chronic kidney disease

WCH and MH are very common in patients with CKD.^165 , 166^The usefulness of HBPM is indisputable in patients with CKD undergoing conservative treatment, peritoneal dialysis (PD), or hemodialysis (HD) and in patients who received a kidney transplant, as it predicts disease progression and the risk of CV events and death.^167^However, the main limitation of HBPM lies in its inability to assess changes in the nocturnal BP pattern, which are common in patients with CKD. Nighttime telemonitoring might be an alternative and is currently under consideration for patients with CKD or who received a kidney transplant.^168^

In patients undergoing conservative treatment and in those who received a kidney transplant, HBPM should be performed according to the usual recommendations.^169^In patients undergoing dialysis, we recommend the following protocol:

- **Number of measurements:** ideally, the patient should take 36 readings.
- **Monitoring period:** 6 days.
- **Day 0 or fitting day:** on this day, BP measurements are initially taken at the office or hemodialysis clinic (ideally 3), with the average of the last 2 readings used to account for a masking/alarm reaction, then at home in the evening to correct for a possible white-coat effect. BP should never be measured on the fistula arm. Home and in-office BP measurements taken on day 0 must be discarded from the average calculation.
- **HBPM days:** at home, BP readings are taken for 6 days. The patient should take 3 readings in the morning and another 3 in the evening/night, always after 5 minutes of rest, before meals, with an empty bladder, and before taking hypertensive medication (if applicable). If the patient has eaten, measurements should be taken only 2 hours after the meal. In patients undergoing typical hemodialysis (2 to 3 times a week), measurements taken on these days should also be excluded from the average calculation; in cases of daily hemodialysis and PD, all measurements will be included.

#### 6.4.6. Obesity

The assessment of BP in patients with obesity presents challenges in clinical practice, as BP variability and the prevalence of WCH and MH is higher in this population than in patients without obesity. Therefore, HBPM is a fundamental tool for identifying hypertension phenotypes in these patients.^170 , 171^However, accurate BP measurement is often hindered by factors such as large arm circumference and/or conical arm shapes, as well as limited availability of appropriately sized cuffs. In this setting, the use of inadequate cuffs may lead to overestimation of BP.^172^In the absence of appropriately sized or shaped cuffs, validated and calibrated wrist devices may serve as an alternative for HBPM in patients with obesity.^144^

#### 6.4.7. Arrhythmias

HBPM devices, especially those currently available on the market, are validated for BP measurement in patients with cardiac arrhythmias, particularly atrial fibrillation.^173^Some automated oscillometric devices are equipped with a specific algorithm that can identify the presence of atrial fibrillation, assisting in the diagnosis of these conditions, especially in older adults.^168 , 174 , 175^

## 7. Cost-effectiveness

Health care costs are a global concern, prompting widespread efforts to contain them. Cost-effectiveness analysis assesses cost (monetary value) in relation to outcomes (effectiveness, eg, lives saved) using various intervention methods.^176 , 177^

A recent cost-effectiveness analysis concluded that HBPM is more effective than conventional in-office BP measurement and requires less financial and human investment than ABPM.^123 , 178^

## 8. Perspectives

In recent years, numerous cuffless devices claiming to accurately measure BP have been made available for use. In general, these devices are equipped with a sensor that assesses arterial pulse and estimates BP mostly through pulse wave analysis (PWA).^179^

This new method is believed to have great potential for allowing multiple or even continuous BP measurements over days or weeks without the discomfort associated with cuff inflation. However, the accuracy and usefulness of cuffless devices are uncertain and require validation in large randomized clinical trials in different clinical settings compared with the gold standard, such as ABPM or invasive measurements. Therefore, at the moment, these devices should not be used for diagnostic or treatment decisions in Brazil.

Nighttime HBPM is already a reality in some countries around the world. This method measures BP during sleep and records nocturnal dipping for a period of days or weeks.^180^However, no such equipment validated by the Brazilian National Health Surveillance Agency is available in Brazil.

## Part 5 – Central blood pressure, pulse wave velocity, and augmentation index

## 1. Introduction

BP values differ significantly between the central and peripheral regions of the arterial tree. SBP is higher in peripheral arteries than in central arteries, while DBP and mean BP differ only slightly. Age and genetics greatly influence the difference between peripheral and central BP curves, and this difference can reach 20 mm Hg for SBP in young individuals. This effect is known as SBP augmentation or PP augmentation. PP augmentation is lower in older people due to increased arterial stiffness (AS) and the early return of the reflected waves, so that central SBP (cSBP) may be close to peripheral SBP. Therefore, the time interval relative to the forward and backward pressure waves in the aorta is considered an important parameter for defining central blood pressure (CBP).^181 , 182^

## 2. Definitions

An increase in BP is directly related to an increase in CV risk due to endothelial dysfunction and damage and increased AS resulting from aggression in the vascular media.^183 - 187^AS is defined as a set of vessel properties that determine their biophysical characteristics, including distensibility, elasticity, and compliance, which interfere with the dynamics of blood flow in each cardiac cycle.^183^Changes in microvessels, arteriosclerosis, and endothelial dysfunction as well as increased AS are injuries resulting from hypertension and other diseases, such as diabetes, obesity, and dyslipidemia, being also influenced by genetic factors and age.^184 , 185 , 188 - 192^Increased AS is the main cause of increased SBP observed with aging.^193^

### 2.1. Pulse wave velocity

Defined as the ratio of the distance traveled between two points in the arterial system to the ventricular ejection wave generated in a unit of time, PWV is measured in meters per second (m/s).^184 , 194 , 195^PWV is the gold standard method for quantifying AS, and an increasing intensity of AS is associated with an increasing PWV.^196^PWV measurement is simple, noninvasive and can be performed in an outpatient setting.^183 , 197^The current reference method in clinical research is carotid-femoral PWV (cfPWV).

### 2.2. Augmentation index

The augmentation index (AIx) is defined as the ratio of the pressure caused by the reflected wave to the ejection wave. This measure is directly related to PWV and inversely related to HR,^192 , 198^and it has the advantage of taking into account the timing of the forward and backward waves, which are the main determinants of CBP.^199 , 200^AIx describes the relationship between CBP and the reflected pulse wave, incorporating the magnitude and velocity of the reflected waves. Consequently, this index can be defined as a measure of the intensity of pulse wave reflections.^201 , 202^

HR should be taken into account to correct AIx, so AIx@75 is the AIx corrected for HR of 75 beats per minute (bpm). This index is reported by AS measurement devices, as a result of applying the following formula:  Alx@75 = Aix - 0,39× (75 - FC) .^203^

### 2.3. Central blood pressure

CBP is the pressure exerted by the blood column, at each beat, on the aorta and carotid arteries, approximating the BP exerted on the heart and brain. For this reason, this marker is often more closely related to CV morbidity and mortality than peripheral BP.^204 , 205^Currently, CBP can be measured noninvasively using several validated methods and devices.^206^

## 3. Indications

Possible indications for CBP, PWV, and AIx are summarized in Table 6 .

**Table 6:** – Possible indications for measuring central blood pressure, augmentation index, and pulse wave velocity (GR: IIa – LE: C)

Parameter	Possible indications
Central systolic blood pressure (cSBP)	Evaluation of spurious hypertension in isolated systolic hypertension in young people
Pulse wave velocity (PWV)	Re-stratification of patients with prehypertension and low-to-intermediate risk hypertension Patients evaluated by SAGE score ≥ 8
Augmentation index (AIx)	Reserved for clinical research setting only

GR: grade of recommendation; LE: level of evidence.

## 4. Advantages of measuring CBP, AIx, and PWV

- Identification of subclinical HMOD: PWV ≥ 10 m/s.^207 , 208^

**Prognostic assessment:** an increase of 1 m/s in PWV is associated with a 14% increase in the occurrence of CV events and a 15% increase in mortality.^205^

- AIx is a predictor of outcomes and HMOD.^209 , 210^
- These measures can improve precision in the diagnosis of hypertension, safety in therapeutic decisions, and definition of prognosis.^211^

## 5. Limitations of measuring CBP, AIx, and PWV

- Poor availability in health centers.
- High-cost devices.
- Measurements obtained by the oscillometric method still require further epidemiological studies, especially regarding their prognostic value.

## 6. Techniques available for checking central and arterial stiffness parameters

Applanation tonometry, as well as piezoelectric mechanoreceptors, sensitive to intravascular pressure, can be used as a substitute for CBP due to the anatomical proximity to the ascending aorta. Applanation tonometry in the radial artery can measure CBP from a generalized mathematical transfer function, but a major drawback of the method is operator dependence. The brachial oscillometric technique has the advantage of being practical for use in the office, in addition to being operator independent. However, it has been criticized for how these devices are calibrated, as they tend to underestimate the true brachial intra-arterial pressure and, consequently, CBP.^1 , 212 - 219^

### 6.1. Methods for indirect measurement of CBP

- **Direct arterial tonometry** : method used in the Complior Analyse® device. This method directly measures pressure waveform in common carotid artery by applanation tonometry and corresponds to the pressure waveform of the ascending aorta.^212 , 213^
- **Indirect arterial tonometry** : method used in the SphygmoCor® device based on brachial artery BP measurement and radial artery applanation tonometry. This method indirectly reconstructs pressure waveform using an algorithm.^212 , 213^
- **Brachial oscillometric method** : method used in the Mobil-O-Graph®, Dyna-MAPA AOP®, and Arteris® devices based on brachial artery BP measurement using an algorithm and transfer function. This method indirectly reconstructs pressure waveform.

Although indirect arterial tonometry is considered the gold standard for the evaluation of AS assessed by cfPWV, oscillometric measurement is a simpler method that provides more reproducible and reliable results.^214 , 215 , 220 , 221^

## 7. Protocols for CBP, PWV, and AIx measurements

### 7.1. Protocol for measuring central parameters using tonometry

In the direct method, piezoelectric sensors are placed in the carotid and femoral arteries to record arterial diameter change curves secondary to changes in intra-arterial pressure with simultaneous recording of central and peripheral signals. CBP is obtained directly from the carotid pressure waveform. It is also possible to evaluate PWV in 3 different arterial segments from a single-point measurement for the study of peripheral arteries.^183 , 219^

In the indirect method, the system consists of an applanation tonometer and an ECG, PWV is measured at 2 points and aligned with the R wave of the ECG QRS complex as a reference point. The central pressure waveform and ascending aortic pressure values are defined using a transfer function.^183 , 219^

### 7.2. Protocol for the triple-trigger procedure (in-office measurement) using the oscillometric method

Depending on the device used, central BP measurements (CBP, PWV, and AIx) obtained by the oscillometric method can be performed at preset time intervals. The preparation steps for in-office BP measurement should be followed, including the choice of appropriate cuff size and arm, as recommended by the 2020 Brazilian Guidelines for Hypertension.^1^

After choosing the arm, a cuff connected to the device programmed to perform a 15-minute monitoring should be applied to the arm, with triggers that assess brachial pressure and central parameters every 3 minutes, considering the last 3 measurements or 3 valid measurements – protocol known as triple PWA. Some devices have a built-in triple PWA function, without the need for presetting.

### 7.3. Protocol for 24-hour central BP parameter measurements using the oscillometric method

The same preparation protocol, patient guidance, and choice of arm/cuff proposed for the 24-hour ABPM should be followed to perform the 24-hour central BP parameter measurements. For improved patient comfort during monitoring, it is recommended that the device be programmed to record BP at 30-minute intervals (daytime and nighttime). A minimum of 16 valid daytime and 8 nighttime BP readings are required. Furthermore, patients should be warned about double cuff inflation during measurements, thus preventing patients from suspecting that an error has occurred during BP measurements.

## 8. Reference values 216 , 218 , 222 - 225

### 8.1. Reference values for PWV

As age and BP have a major influence on AS, reference values for PWV are usually presented in categories derived from these variables. In Tables 7 and 8, reference values are suggested for PWV, whose measurements were obtained mainly using a tonometer or piezoelectric sensors.^226^

### 8.2. Reference values for cSBP

The reference values for cSBP using the SphygmoCor, Omron HEM-9000AI, PulsePen, and direct carotid artery tonometry devices and categorized by age, sex, and risk factors and for peripheral BP are shown in Tables 9 and 10.^227^

### 8.3. Reference values for PWV, CBP, and AIx using the oscillometric method in the Brazilian population

A Brazilian multicenter study, including 6499 individuals from 4 centers, described reference values for PWV, cSBP, and AIx in the Brazilian population measured using the oscillometric method.^212^Tables 11 and 12 show the reference values for these measurements according to age group, sex, and CV risk factors.^214^

In view of the foregoing, we can conclude that there are currently reference values for the different AS and CBP measurement devices, including for the Brazilian population. However, little is still known about the abnormality thresholds for the measurement of central BP parameters capable of predicting CV events, especially when obtained using the oscillometric method. The suggested abnormality thresholds for the measurement of central BP parameters are shown in Table 13 .

**Table 13:** – Abnormality thresholds for CBP, PWV, and AIx (GR: IIa – LE: C). 216 , 218 , 222 - 225

Parameters	In-office measurement	24-hour ABPM	Daytime ABPM	Nighttime ABPM
Central SBP	≥ 130 mm Hg	≥ 120 mm Hg	≥ 125 mm Hg	≥ 110 mm Hg
Central DBP	≥ 90 mm Hg	≥ 80 mm Hg	≥ 85 mm Hg	≥ 70 mm Hg
vPWV	≥ 10 m/s	≥ 10 m/s	≥ 10 m/s	≥ 10 m/s
AIx	Not available			

GR: grade of recommendation; LE: level of evidence; ABPM: ambulatory blood pressure monitoring; CBP: central blood pressure; PWV: pulse wave velocity; AIx: augmentation index; vPWV: venous pulse wave velocity.

## 9. Prognostic value of BP measurements derived from central parameters

Evidence indicates that CBP is more closely associated with traditional CV risk factors and HMOD. AS is one of the main determinants of CBP and has been considered to have high predictive value for CV events. Likewise, prospective observational studies have demonstrated the predictive value of central hemodynamic parameters for CV events in the general population, in older people, and in patients with coronary disease and CKD.^228^

In the Strong Heart Study, central PP (cPP) was superior to peripheral PP as a predictor of fatal and non-fatal CV events.^229^Other studies have found equal superiority in normotensive or hypertensive older people and in Asian individuals.^227 , 228^

In the Multiethnic Study of Atherosclerosis (MESA), pulse wave reflection parameters were associated with new-onset CV events and incidence of HF in individuals without evidence of prior CVD.^229 , 230^

In a meta-analysis involving more than 5000 normotensive and hypertensive individuals, some with coronary disease or CKD, for every 10 mm Hg increase in cPP and in cSBP, there was a 14% increase in CV risk and a 9% increase in CVD risk, respectively.^231^Average 24-hour ambulatory BP values have also been considered better predictors of CV risk than peripheral BP measurements in normotensive and hypertensive patients.^232^

In patients with earlier stage CKD, cPP was able to predict progression to end-stage renal disease and, in stages 2 to 4, an independent relationship between cSBP and overall mortality was demonstrated.^233^The adverse effect of increased arterial pulse wave reflection on the CV system of patients with end-stage renal disease has also been demonstrated.^234^

The clinical value of CBP measurement was initially demonstrated in the Anglo-Scandinavian Cardiac Outcomes Trial – Conduit Artery Function Evaluation (ASCOT-CAFE). The main ASCOT study had already reported a more favorable CV outcome in hypertensive patients treated with a combination treatment regimen of an angiotensin-converting enzyme inhibitor (ACEI) with a calcium channel blocker (CCB) compared with therapy based on beta-blockers and diuretics.^235 , 236^

In the CAFE substudy, although there was no difference in peripheral BP levels between the two groups, treatment with ACEI + CCB promoted a greater reduction in CBP.^45^

The REASON study was able to demonstrate that a favorable effect of the combination of ACEI + diuretics on the reflection coefficient was present even after 9 months of treatment, although without additional effect on peripheral BP.^237^

The prediction of CV events and all-cause mortality through the analysis of central hemodynamics measured with noninvasive methods allowed the calculation of the predictive value of central pressures and central hemodynamic indexes for CV events and all-cause mortality by measuring PWV, cSBP, cPP, and AIx.^205^The following could be stated: for an increase in aortic PWV of 1 m/s, the risk of total CV events, CV mortality, and all-cause mortality increases by 14%, 15%, and 15%, respectively; an increase of 10 mm Hg in cSBP determines a relative risk (RR) of 8.8% for CV events; an increase of 10 mm Hg in cPP determines an RR of 12.9% for CV events; an increase of 10% in AIx determines an RR of 29.4% for total CV events and an RR of 38.4% for all-cause mortality.^205^

PWV has predictive value in CV morbidity and mortality and is currently considered the gold standard method in the assessment of AS and arterial aging, with good correlation with the risk of CV death, CV events, and all-cause mortality.^197 , 205^

To allow a practical identification of individuals at greater risk of developing increased PWV, a clinical score that evaluates easily available variables, called SAGE score, was developed and validated: (S) *systolic blood pressure* , (A) *age* , (G) *fasting plasma glucose* , and (E) *estimated glomerular filtration rate* (estimated by CKD-EPI), summarized in Figure 9 .^242^This score was also applied to Brazilian hypertensive patients evaluated with the oscillometric method, and a SAGE score of ≥ 8 was effective in identifying patients with a high risk of PWV ≥ 10 m/s, with a sensitivity of 67.19% (95% CI, 60.1-73.8) and a specificity of 93.95% (95% CI, 91.8-95.7).^239^

**Figure 9:** – SAGE score chart. 238 , 239 In orange (SAGE ≥ 8): high probability of elevated arterial stiffness (PWV ≥ 10 m/s). In green (SAGE < 8): low probability of elevated arterial stiffness; PWV: pulse wave velocity.

## 10. Measurement of central parameters over 24 hours

The assessment of CBP and AS indicators over 24 hours is still little used in clinical practice, even with growing evidence worldwide of their predictive validity.^217^There is a need to expand scientific knowledge of 24-hour monitoring. However, evidence indicates that monitoring these parameters in daily life conditions can favor the assessment and clinical prognosis of CVD, the possibility of categorizing hypertension phenotypes, especially WCH and MH, and the specific investigation of daytime and nighttime BP.^1 , 45 , 214 , 217^

The International 24-Hour Aortic Blood Pressure Consortium (20 centers, 14 countries, and 5 continents), with central parameters obtained using a Mobil-O-Graph® monitor, conducted a pooled analysis of part of its database (2092 adults) and showed that 24-hour cSBP was more closely associated with hypertensive cardiac organ damage (left ventricular mass and hypertrophy) than 24-hour brachial cSBP. The same group, in a recent publication, using 130,804 valid cSBP measurements from 2423 untreated adults, pragmatically proposed 120 mm Hg as the upper normal limit for 24-hour cSBP (C1 calibration = systolic/diastolic).^222 , 240^

A recent meta-analysis reviewed current 24-hour noninvasive technologies for cSBP, PWV, and AIx and the evidence supporting their use in the clinical treatment of patients with hypertension or at risk of CV complications and concluded that the studies performed to date suggest that 24-hour central parameters may represent a promising tool for assessing vascular function, structure, and damage in daily life conditions and promoting early screening in individuals at increased risk.^217^There is still a paucity of studies evaluating the predictive value of 24-hour PWV. Furthermore, the precision of measurements may vary from device to device, affecting the generalization of study results. Longitudinal studies are still needed to validate the predictive value of 24-hour central parameters.

## 11. Perspectives

The assessment of AS in the range of recommendations for CV risk stratification as a way of identifying the presence of still subclinical HMOD has already been incorporated into the main guidelines.^1 , 216 , 217^In this context, the analysis of AS by PWV and central hemodynamic indexes (SBP, cPP, and AIx) are useful for clinical monitoring of patients with hypertension and low-to-intermediate CV risk, young patients with hypertension, patients with prehypertension, diabetes, or CKD, patients with a family history of early CVD, and other risk factors.^205 , 207 , 241 - 245^Although the use of PWV for the identification of HMOD is well established in the literature, as well as its cutoff point of > 10 m/s, values that define the normality threshold for PWV, corrected for sex and age, remain to be better explored.^1 , 216 , 218 , 223 - 225^

Based on the interactions of genetics and epigenetics and the ability of the biological system to deal with the results of these combinations, there may be a difference between chronological and biological age. This is the basis for new concepts in vascular aging. Accelerated vascular aging occurs in individuals whose biological vascular age is greater than their chronological age, and these individuals will present earlier disease manifestations. The term SUPERNOVA (supernormal vascular aging) is reserved for individuals who present a vascular age in which arterial injury/stiffness is significantly lower than that expected for a healthy individual of the same age. However, even though this ability to age slowly might be genetically predetermined, lifestyle and pharmaceutical interventions can slow vascular senescence and improve prognosis.^206 , 219 , 246^

Studies that guide drug treatment using cSBP, to date, have only demonstrated a reduction in the number of antihypertensive drugs, with no effect on left ventricular function.^247 - 249^In a study evaluating the use of free-dose vs fixed-dose drug combinations to achieve central and brachial SBP goals, ACEIs in a fixed-dose combination were more effective in achieving BP goals. Based on the repercussions of the CAFE study, it became clear that drugs have different effects on BP values (central and peripheral), which result in different clinical outcomes.^250 , 251^

All these aspects allow us to conclude that there is considerable potential for a true evolution toward precision medicine in its purest concept: identifying damage early or even preventing it from occurring and treating each individual with the most appropriate strategy for their clinical characteristics, thus further reducing CV outcomes.

In conclusion, these Guidelines do not recommend the routine use of cSBP, PWV, and AIx assessment. To this end, further studies with robust clinical outcomes are still required to define the normality thresholds and prognosis for these measurements.

**Figure 1:** – BP patterns and their prevalence in individuals without antihypertensive medication use. 43 , 45 HBPM: home blood pressure monitoring; ABPM: ambulatory blood pressure monitoring.

**Figure 2:** – BP patterns and their prevalence in individuals with antihypertensive medication use. 43 , 45 HBPM: home blood pressure monitoring; ABPM: ambulatory blood pressure monitoring.

**Tabela 7:** – PWV reference values categorized by age in a healthy population ^**226**^

Age (years)	Mean (+2 SD)	Median (p10- p90)
30	6.2 (4.7-7.6)	6.1 (5.3-7.1)
30-39	6.5 (3.8-9.2)	6.4 (5.2-8.0)
40-49	7.2 (4.6-9.8)	6.9 (5.9-8.6)
50-59	8.3 (4.5-12.1)	8.1 (6.3-10.0)
60-69	10.3 (5.5-15.0)	9.7 (7.9-13.1)
≥70	10.9 (5.5-16.3)	10.6 (8.0-14.6)

PWV: pulse wave velocity; SD: standard deviation; p10: upper 10th percentile; p90: lower 90th percentile.

**Table 8:** – PWV reference values categorized by age and blood pressure ^**226**^

Age (years)	Optimal BP	Normal BP	High-normal BP	Stage 1 hypertension	Stage 2/3 hypertension
**Mean PWV (+2 SD)**					
< 30	6.1 (4.6-7.5)	6.6 (4.9-8.2)	6.8 (5.1-8.5)	7.4 (4.6-10.1)	7.7 (4.4-11.0)
30-39	6.6 (4.4-8.9)	6.8 (4.2-9.4)	7.1 (4.5-9.7)	7.3 (4.0-10.7)	8.2 (3.3-13.0)
40-49	7.0 (4.5-9.6)	7.5 (5.1-10.0)	7.9 (5.2-10.7)	8.6 (5.1-12.0)	9.8 (3.8-15.7)
50-59	7.6 (4.8-10.5)	8.4 (5.1-11.7)	8.8 (4.8-12.8)	9.6 (4.9-14.3)	10.5 (4.1-16.8)
60-69	9.1 (5.2-12.9)	9.7 (5.7-13.6)	10.3 (5.5-15.1)	11.1 (6.1-16.2)	12.2 (5.7-18.6)
≥ 70	10.4 (5.2-15.6)	11.7 (6.0-17.5)	11.8 (5.7-17.9)	12.9 (6.9-18.9)	14.0 (7.4-20.6)
**Median PWV (p10- p90)\**					
< 30	6.0 (5.2-7.0)	6.4 (5.7-7.5)	6.7 (5.8-7.9)	7.2 (5.7-9.3)	7.6 (5.9-9.9)
30-39	6.5 (5.4-7.9)	6.7 (5.3-8.2)	7.0 (5.5-8.8)	7.2 (5.5-9.3)	7.6 (5.8-11.2)
40-49	6.8 (5.8-8.5)	7.4 (6.2-9.0)	7.7 (6.5-9.5)	8.1 (6.8-10.8)	9.2 (7.1-13.2)
50-59	7.5 (6.2-9.2)	8.1 (6.7-10.4)	8.4 (7.0-11.3)	9.2 (7.2-12.5)	9.7 (7.4-14.9)
60-69	8.7 (7.0-11.4)	9.3 (7.6-12.2)	9.8 (7.9-13.2)	10.7 (8.4-14.1)	12.0 (8.5-16.5)
≥ 70	10.1 (7.6-13.8)	11.1 (8.6-15.5)	11.2 (8.6-15.8)	12.7 (9.3-16.7)	13.5 (10.3-18.2)

PWV: pulse wave velocity; BP: blood pressure; SD: standard deviation; p10: upper 10th percentile; p90: lower 90th percentile.

**Table 9:** – VCBP reference values categorized by age, sex, and risk factors ^**227**^

Age (years)	Healthy population		Population with cardiovascular risk factors	
	Female	Male	Female	Male
< 20 (n = 1104)	97 (86, 91, 102, 109) n = 350	105 (95, 99, 109, 113) n = 290	99 (88, 93, 105, 120) n = 182	109 (96, 102, 117, 127) n = 282
20-29 (n = 4157)	95 (80, 88, 102, 110) n = 1411	103 (92, 97, 109, 115) n = 880	101 (88, 91, 110, 124) n = 888	110 (95, 102, 120, 130) n = 974
30-39 (n = 6386	98 (84, 90, 108, 119) n = 1860	103 (88, 95, 112, 120) n = 1259	111 (92, 100, 127, 141) n = 1373	114 (95, 103, 129, 144) n = 1889
40-49 (n = 9595)	102 (87, 93, 113, 123) n = 2318	106 (90, 97, 114, 123) n = 2068	116 (95, 104, 133, 146) n = 2196	118 (97, 106, 132, 144) n = 2995
50-59 (n = 11950)	110 (93, 100, 119, 127) n = 2002	110 (96, 102, 118, 126) n = 1997	120 (100, 109, 134, 148) n = 4251	123 (102, 111, 137, 150) n = 3646
60-69 (n = 7779)	114 (97, 105, 122, 129) n = 1057	114 (97, 105, 122, 128) n = 1410	128 (105, 115, 141, 154) n = 2656	128 (105, 115, 142, 155) n = 2629
> 70 (n = 4445)	118 (100, 109, 126, 131) n = 530	116 (99, 107, 124, 130) n = 747	138 (113, 126, 152, 164) n = 1567	135 (113, 124, 147, 160) n = 1592

Values described in the 50th percentiles (10th, 25th, 75th, and 90th).

**Table 10:** – BP reference values according to BP classification and cardiovascular risk factors ^**227**^

Blood pressure category\	Healthy population		Population with cardiovascular risk factors	
	Female	Male	Female	Male
Optimal (n = 17678) 108 (96, 102, 114, 117)	97 (84, 90, 104, 110) n = 6415	100 (88, 94, 106, 111) n = 4035	102 (89, 95, 108, 112) n = 4082	101 (90, 96, 107, 112) n = 3146
Normal (n = 9313) 123 (120, 121, 126, 128)	116 (104, 110, 121, 125) n = 1902	112 (102, 106, 117, 122) n = 2669	116 (107, 111, 120, 123) n = 2281	113 (103, 108, 118, 122) n = 2461
High-normal (n = 7148) 133 (128, 130, 136, 138)	126 (115, 120, 131, 135) n = 1212	122 (110, 115, 128, 132) n = 1947	125 (116, 120, 130, 133) n = 1861	123 (111, 116, 128,132) n = 2128
Stage 1 (n = 3288) 143 (130, 137, 150, 155)			137 (122, 129, 144, 150) n = 1276	133 (119, 126, 142, 148) n = 2012
Stage 2 (n = 1930) 161 (146, 154, 168, 174)			154 (128, 142, 161, 168) n = 798	148 (128, 138, 158, 165) n = 1132
Stage 3 (n = 701) 183 (162, 178, 193, 206)			173 (153, 164, 183, 194) n = 312	171 (143, 158, 183, 192) n = 389
ISH (n = 5255) 147 (141, 143, 155, 163)			140 (128, 134, 148, 156) n = 2507	137 (122, 129, 144, 152) n = 2748

Values described in the 50th percentiles (10th, 25th, 75th, and 90th). Blood pressure (BP) categories refer to brachial artery blood pressure.

**Table 11:** – CBP reference values according to sex and age in a healthy population and to cardiovascular risk factors 214

Age (years)	Healthy population		Population with cardiovascular risk factors	
	Female	Male	Female	Male
**Variable: Central systolic blood pressure**				
< 30	101 (90, 93, 113, 119)	113 (104, 109, 120, 123)	118 (102, 109, 127, 131)	123 (107, 114, 132, 144)
30-39	109 (96, 102, 117, 123)	114 (102,110,121, 127)	120 (102, 110, 130, 143)	125 (108, 116, 133, 141)
40-49	110 (99, 103, 117, 122)	116 (102,109, 122, 126)	121 (104, 112, 134, 146)	123 (108, 115, 131, 141)
50-59	110 (97, 104, 120, 124)	112 (100, 106, 118, 124)	124 (106, 114, 135, 146)	124 (105, 114, 134, 144)
60-69	114 (100, 105, 120, 125)	112 (96, 101, 120, 127)	127 (105, 115, 141, 154)	123 (103, 112, 136, 149)
≥ 70	113 (100, 103, 121, 126)	116 (94, 104, 125, 129)	131 (108, 118, 146, 165)	125 (102, 111, 140, 156)
**Variable: Central diastolic blood pressure**				
< 30	73 (60, 66, 77, 85)	76 (66, 71, 82, 87)	82 (68, 73, 90, 97)	83 (72, 77, 93, 100)
30-39	77 (67, 71, 83, 88)	80 (71, 75, 85, 88)	86 (71, 77, 95, 105)	88 (75, 80, 96, 103)
40-49	79 (67, 73, 84, 88)	81 (74, 77, 86, 89)	86 (71, 78, 94, 103)	90 (75, 82, 97, 104)
50-59	76 (64, 70, 82, 85)	82 (70, 77, 86, 88)	84 (71, 77, 92, 100)	88 (75, 80, 97, 103)
60-69	76 (66, 71, 81, 87)	80 (68, 72, 83, 87)	81 (67, 74, 90, 98)	85 (71, 77, 93, 101)
≥ 70	76 (60, 70, 79, 83)	79 (60, 70, 84, 90)	81 (66, 72, 89, 97)	82 (68, 74, 91, 98)
**Variable: Central pulse pressure**				
< 30	29 (23, 27, 37, 43)	36 (26, 32, 43, 53)	34 (24, 28, 41, 48)	38 (26, 31, 46, 52)
30-39	30 (22, 26, 37, 44)	35 (25, 29, 42, 50)	34 (24, 28, 38, 46)	36 (25, 31, 41, 48)
40-49	31 (22, 27, 36, 42)	32 (25, 28, 38, 45)	35 (25, 29, 43, 53)	33 (23, 28, 37, 46)
50-59	34 (25, 28, 42, 49)	30 (25, 27, 35, 42)	39 (28, 32, 47, 58)	34 (25, 28, 41, 49)
60-69	35 (28, 31, 43, 52)	31 (24, 28, 36, 49)	44 (30, 36, 55, 66)	37 (25, 31, 46, 58)
≥ 70	39 (28, 34, 45, 52)	37 (19, 27, 41, 51)	50 (33, 41, 63, 77)	42 (28, 34, 52, 66)

CBP: central blood pressure. Values described in the 50th percentiles (10th, 25th, 75th, and 90th).

**Table 12:** – PWV and AIx reference values according to sex and age in a healthy population and to cardiovascular risk factors ^214^

Age (years)	Healthy population		Population with cardiovascular risk factors	
	Female	Male	Female	Male
**Variable: PWV**				
< 30	4.9 (4.4, 4.5, 5.0, 5.3)	5.2 (4.9, 5.1, 5.4, 5.7)	5.3 (4.7, 5.0, 5.6, 6.0)	5.5 (5.0, 5.3, 5.8, 6.3)
30-39	5.4 (5.0, 5.2, 5.8, 6.1)	5.7 (5.3, 5.5, 5.9, 6.1)	5.8 (5.3, 5.5, 6.2, 6.7)	6.1 (5.5, 5.8, 6.4, 6.7)
40-49	6.4 (5.7, 6.0, 6.7, 6.9)	6.5 (5.9, 6.2, 6.8, 7.0)	6.8 (6.0, 6.4, 7.2, 7.7)	6.8 (6.2, 6.4, 7.1, 7.5)
50-59	7.5 (6.7, 7.0, 7.8, 8.2)	7.4 (6.9, 7.2, 7.9, 8.0)	7.9 (7.1, 7.5, 8.3, 8.8)	7.9 (7.1, 7.5, 8.3, 8.7)
60-69	8.9 (8.1, 8.5, 9.2, 9.4)	8.9 (8.2, 8.6, 9.1, 9.6)	9.3 (8.4, 8.8, 9.8, 10.4)	9.2 (8.4, 8.7, 9.7, 10.2)
≥ 70	11.3 (10.2, 10.4, 12.5, 13.2)	11.0 (10.1, 10.6, 11.6, 12.3)	11.8 (10.2, 10.8, 12.9, 14.0)	11.2 (9.9, 10.4, 12.1, 13.2)
**Variable: AIx**				
< 30	20 (11, 13, 27, 33)	16 (04, 10, 23, 27)	28 (11, 20, 34, 38)	16 (02, 08, 23, 30)
30-39	22 (12, 16, 28, 34)	14 (01, 07, 18, 24)	26 (11, 18, 32, 37)	15 (03, 09, 21, 27)
40-49	23 (09, 15, 29, 35)	15 (00, 06, 21, 25)	25 (10, 17, 34, 38)	15 (02, 08, 23, 30)
50-59	22 (07, 12, 33, 39)	12 (02, 04, 19, 22)	24 (08, 14, 33, 39)	15 (03, 07, 24, 32)
60-69	23 (09, 14, 34, 42)	17 (01, 05, 27, 43)	28 (11, 18, 37, 44)	17 (03, 09, 26, 34)
≥ 70	28 (11, 20, 39, 42)	22 (05, 10, 33, 41)	33 (17, 25, 42, 48)	22 (04, 12, 31, 41)

PWV: pulse wave velocity; AIx: augmentation index. Values described in the 50th percentiles (10th, 25th, 75th, and 90th).

**Chart 1:** – Korotkoff sounds

PHASES	SOUNDS
Phase I	Appearance of a first, weak sound, followed by repetitive tapping sounds corresponding to systolic BP
Phase II	Sounds become softer and longer, like an intermittent murmur
Phase III	Sounds become crisper
Phase IV	Sounds become muffled
Phase V	Sounds corresponding to systolic BP completely disappear

**Chart 2:** – Disadvantages of the auscultatory method compared with the oscillometric method


Rounding off of the last digit of BP values to 0 or 5
Incorrect positioning of the aneroid manometer in relation to the eye
Excessive stethoscope pressure causing artery deformation
Excessive cuff inflation causing pain
Rapid cuff deflation leading to underestimation of systolic BP and/or overestimation of diastolic BP
Incorrect identification of systolic and diastolic BP sounds
Possibility of aneroid manometer being out of calibration, even when the pointer is at zero
Does not allow unattended automated office BP measurement

BP: blood pressure.

**Chart 3:** – Unattended automated office BP measurement

Instructions
Perform BP measurement in a calm environment, preferably an empty room
Explain the importance of the procedure to the patient
Follow the patient preparation steps for BP measurement using automated monitors (Chart 4)
Ask everyone to leave the room
Instruct the patient to press the Start button to begin measurements when alone in the room
Instruct the patient to wait approximately 1 minute between each measurement
Instruct the patient to take 3 consecutive measurements
Leave an illustrative leaflet with instructions on the procedure in a visible place

BP: blood pressure.

**Chart 5:** – How to estimate systolic blood pressure

Estimation of systolic BP
Place the cuff (Chart 6) 2 to 3 cm above the antecubital fossa, positioning the center of the cuff bladder directly over the brachial artery
Palpate the radial artery
Close the bulb’s valve and inflate the cuff until the pulse disappears
Open the valve slowly to deflate the cuff
Identify systolic blood pressure by the palpatory method (pulse reappearance)

BP: blood pressure.

**Chart 6:** – How to measure arm circumference

Measurement of arm circumference
Bent the arm at the elbow at a 90-degree angle
Measure the distance between the acromion and the olecranon processes to determine the midpoint of the arm
Straighten the arm and measure the circumference at the midpoint
Choose the cuff according to arm circumference size. The width of the inflatable cuff bladder must cover 37%-50% of the arm circumference, and the length must cover 75%-100% (Chart 7).
Do not place the cuff over clothing.

**Chart 7:** – Cuff dimensions according to arm circumference

Circumference	Cuff category	Width of the inflatable cuff bladder	Length of the inflatable cuff bladder
≤ 6 cm	Infant	3 cm	6 cm
6-15 cm	Small child	5 cm	15 cm
16-21 cm	Child	8 cm	21 cm
22-26 cm	Small adult	10 cm	24 cm
27-34 cm	Adult	13 cm	30 cm
35-44 cm	Large adult	16 cm	38 cm
45-52 cm	Thigh	20 cm	42 cm

**Chart 16:** – Activities of the health professional in charge of fitting the ABPM monitor

On the ABPM day, the health professional in charge of fitting the monitor must follow the instructions described below:
***1.*** Provide detailed explanation of the procedure to patients and tell patients to follow their usual daily activities during the monitoring period.
***2.*** Tell patients to follow their doctor’s advice about medication use.
***3.*** Tell patients not to engage in physical activity during the monitoring period.
***4.*** Measure weight and height.
***5.*** Measure the arm circumference and select the appropriate cuff size (width and length).
***6.*** Before fitting the monitor, measure BP in the sitting position, after a 5-minute rest, in both arms, preferably simultaneously, using the auscultatory or oscillometric method.
***7.*** Apply the cuff to the nondominant arm if systolic BP difference between arms is < 10 mm Hg. If ≥ 10 mm Hg, apply the cuff to the arm with the higher BP values.
***8.*** Place the cuff 2 to 3 cm above the cubital fossa, following the specific device manufacturer’s instructions.
***9.*** Program the monitor following the patient’s information to define daytime and nighttime periods. Follow the guidelines provided under the item “HBPM protocol.”
***10.*** After fitting the monitor, compare the BP measurement obtained by ABPM with that obtained by the auscultatory or oscillometric method, ensuring that the difference is no greater than 5 mm Hg.
***11.*** Ensure that patients have clearly understood all instructions and are confident to contribute appropriately to the monitoring.
***12.*** Perform at least 2 trial measurements before releasing the patient.

ABPM: ambulatory blood pressure monitoring; BP: blood pressure.

**Chart 17:** – Activities of the health professional in charge of removing the ABPM monitor

*1.* Check the completion of the activity diary with patients, particularly regarding the time of drug intake, duration and quality of sleep, and reporting of “occasional events.”
***2.*** Perform at least 2 trial measurements.
***3.*** Subjectively analyze the quality of the activities performed by patients during the monitoring period, for example: whether patients followed their usual daily activities, whether they felt limited in their activities due to discomfort caused by cuff inflations, among others. These events must be considered when interpreting the results and issuing the report.
***4.*** Check the number of valid readings during daytime and nighttime measurements. If minimum requirement is not met, inform the patient that measurement should be repeated.
***5.*** Download the data from the monitor to the computer and enter information such as: weight, height, medications (with dose and number of doses), and the time of main activities (going to bed, rising, breakfast, lunch, dinner).

ABPM: ambulatory blood pressure monitoring.
